# A field‐deployable CRISPR-based biosensing platform for monitoring marine ecosystems

**DOI:** 10.1038/s41893-025-01752-0

**Published:** 2026-01-27

**Authors:** Nayoung Kim, Daniel S. Collins, Nina M. Donghia, Benjamin S. Miller, Hani M. Sallum, Silvi R. Lybbert, Elena Perini, James B. Niemi, James J. Collins, Peter Q. Nguyen

**Affiliations:** 1 Wyss Institute for Biologically Inspired Engineering, Harvard University, Boston, MA 02215, USA; 2 Institute for Medical Engineering and Science, Massachusetts Institute of Technology, Cambridge, MA 02139, USA; 3 Broad Institute of MIT and Harvard, Cambridge, MA 02139, USA; 4 Nicholas School of the Environment and Department of Biology, Duke University, NC 27710, USA; 5 Department of Medical Physics, University College London, London WC1E 6BT, UK; 6 Department of Biochemical Engineering, University College London, London WC1E 6BT, UK; 7 Faculty of Medicine, Imperial College London, London, UK; 8 Department of Biological Engineering, Massachusetts Institute of Technology, Cambridge, MA 02139, USA

## Abstract

Ocean ecosystems are undergoing accelerating disruption from human impacts such as climate change. Warming ocean temperatures drive pathogenic outbreaks, increase harmful algal blooms, and cause coral stress. These can have serious consequences for marine ecosystems, human health and the aquaculture industry, representing a critical One Health issue. Monitoring key marine species offers valuable insights, but current methods are resource-intensive, low-resolution, and unsuitable for frequent deployment. Here, we introduce a low-cost, field-deployable CRISPR biosensing platform for detecting marine organismal DNA and RNA. Harnessing the programmability of CRISPR diagnostics for environmental biosurveillance, we demonstrate versatility across three climate-linked indicators: *Vibrio* spp., *Pseudo nitzschia* spp., and heat-stressed corals. Portable 3D-printed processor and incubator devices enable direct processing of filter-captured samples with temperature control. Field-readiness is reinforced by lyophilized reagents, lateral-flow readouts, dropper-based handling, and a two-step multiplexed workflow, delivering results within one hour without laboratory instruments. Benchmarking with authentic pathogens and environmental seawater confirmed seawater tolerance and robust detection of 10^8^ CFU/filter *Vibrio* pathogens, equivalent to 10^2^ cp/µL for a 1L filtered sample. This decentralized platform reduces barriers to routine monitoring and can provide early warnings of ecosystem disturbances, whilst supporting One Health initiatives in the marine space.

As we navigate the Anthropocene epoch, understanding human impacts on ecosystems is crucial, with the ocean a key nexus connecting geochemical cycles, planetary climate, and foundational ecosystems^1^. Climate change disproportionately affects the ocean, which has absorbed approximately 90% of excess atmospheric heat over the past five decades^2^. This warming has disrupted marine communities, resulting in definable impacts on natural biota, the aquaculture industry, and human health. Monitoring key marine ‘barometer’ species can provide critical insights into the pulse of ocean health, reflecting both local biota and global long-term trends^3–6^. Such information is vital for informing climate change policy, supporting One Health collaborative principles, and driving effective remediation and stewardship solutions.

However, current biomonitoring surveillance methods are constrained by low resolution or high resource demands^7^. Satellite-based ocean imaging, for example, detects harmful algal blooms only after they have matured to sufficiently large sizes^8^. Automated robotic systems such as the 1000-lb Environmental Sample Processor (ESP) enable sophisticated real-time analyses and wireless data transmission while anchored in the ocean, but it is prohibitively expensive, restricting widespread deployment^9^. Bacterial cultures inoculated from ocean samples can be identified by biochemical profiles, but this process takes several days^10,11^. Alternatively, PCR-based methods or next generation sequencing (NGS) offer species identification through the selective amplification of organismal genes or transcripts^12,13^. Yet, both require specialized instrumentation, trained personnel, and long analysis times of up to multiple days^14^. Larger marine organisms continue to be monitored primarily by visual surveys, which are time-consuming, labor-intensive and limited in sampling depth and throughput^15^. Collectively, these limitations underscore the urgent need for advanced ocean health biosurveillance – inexpensive, laboratory-free approaches capable of rapidly identifying marine species on-site, allowing for the swift assessment of potential outbreaks of pathogenic or toxic marine organisms as well as routine monitoring of critically threatened species.

Breakthrough CRISPR–Cas enzyme platforms, represented by SHERLOCK (specific high-sensitivity enzymatic reporter unlocking)^16^ and DETECTR (DNA endonuclease-targeted CRISPR trans reporter)^17^ approaches, harness the unique properties of these enzymes for rapid and low-cost nucleic acid detection^18^. These ribonucleoproteins exhibit nonspecific nuclease activity triggered only upon specific recognition of a target nucleic acid sequence. The consequent “collateral” cleavage of short nucleic acid probes provides an intrinsic signal amplification strategy for detection. Combined with target amplification techniques such as recombinase polymerase amplification (RPA) and varying downstream readouts (e.g., fluorescence, lateral-flow), CRISPR-based molecular sensors routinely achieve attomolar sensitivity for a wide array of biological targets^19–23^. The exceptional programmability of these CRISPR-based biosensors enables rapid reconfiguration to diverse targets while preserving high specificity^24^. Most CRISPR-based diagnostics efforts have centered on medical diagnostics, with limited translation into practical on-site environmental biosurveillance^25^. To our knowledge there has not been an integrated system developed for field analysis of ocean samples^26,27^. Broader adoption for environmental samples has been hampered by the bottleneck of sample preparation, which often depends on harsh lysis protocols, extensive processing, and specialized laboratory infrastructure.

Here we describe a field-deployable, low-cost, and easy-to-use CRISPR-based nucleic acid biosensing platform for rapid marine species surveillance (Fig. 1a). Our on-site portable platform enables sensitive detection of DNA signatures for accurate species identification and biomarker RNA transcripts for probing organismal physiological states or stress responses. We demonstrate programmability and versatility across three representative cases shaped by climate change: pathogenic Vibrio bacteria linked to human health and aquaculture; harmful algal blooms (HABs) responsible for mass die-offs and human illness; and coral mRNA stress transcripts upregulated during early bleaching. A 3D-printed disposable processor paired with a low-cost, reusable 3D-printed incubator enclosing a rechargeable hand-warmer enables in-filter lysis and target amplification directly from captured samples. Amplified nucleic acids are subsequently detected by CRISPR-based biosensors through a simple colorimetric lateral-flow readout. All reagents were optimized for lyophilization, ensuring stability at room temperature and readiness for field use. The full workflow is completed within about one hour using hand-squeezing or drop-based handling and requires no laboratory infrastructure. Potential users span across the One Health collaborative spectrum - marine biologists, ecologists, citizen scientists, coastal communities, conservationists, aquaculture farmers, resource managers, rangers, and biosecurity officers. We benchmarked this field-deployable platform with environmental samples and authentic pathogens to demonstrate its practicality, enabling it to be implemented on research vessels, fishing boats, aquaculture farms, and coastal stations for the rapid biomonitoring of diverse marine organisms.

## Results

### CRISPR-based biosurveillance for marine ecosystems

**Figure 1:**
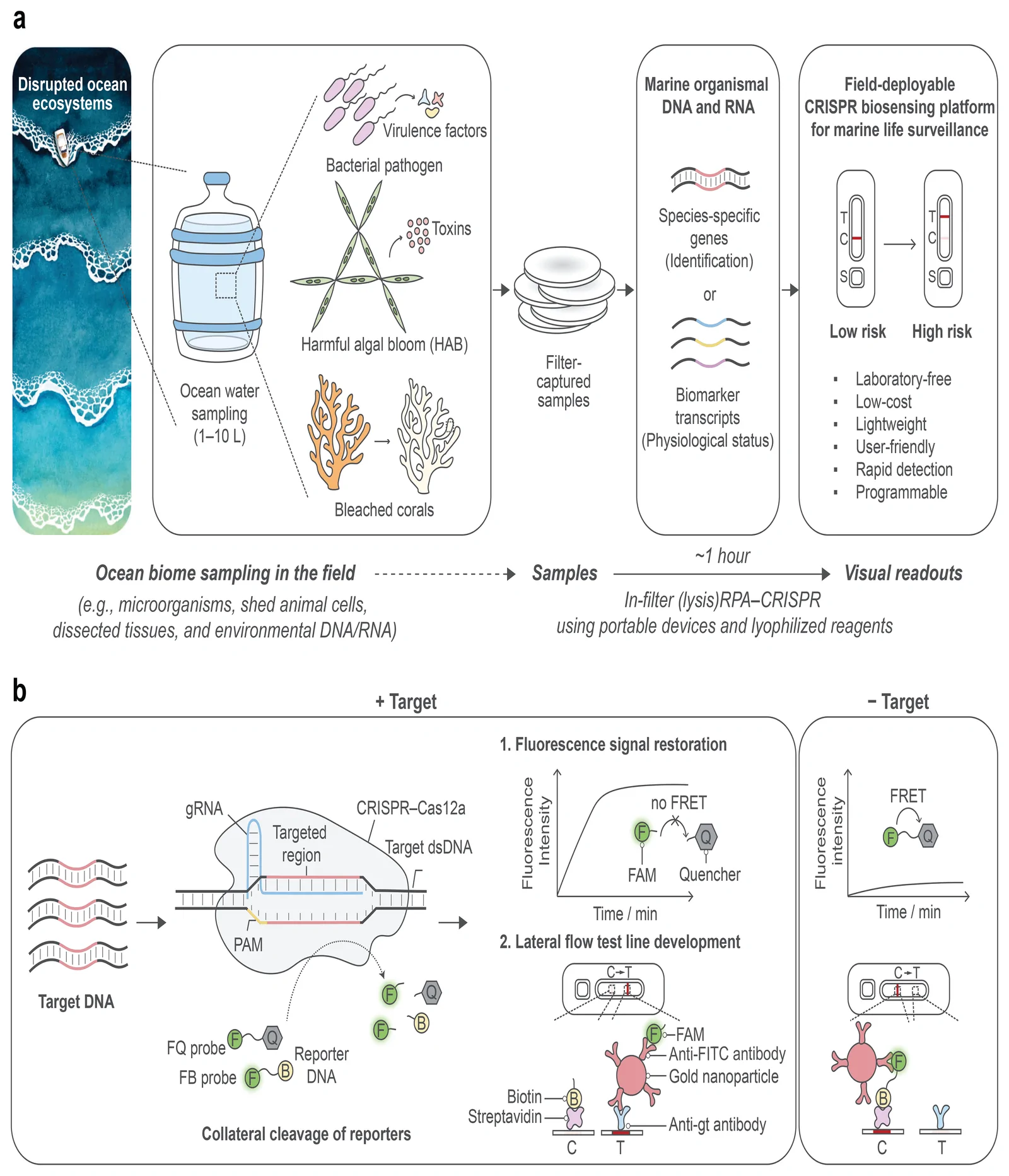
Field-deployable CRISPR biosensing platform for sustainable marine surveillance. (a) Climate change and anthropological pollution have disrupted ocean ecosystems and reshaped marine biomes, driving pathogen outbreaks, toxin-producing harmful algal blooms, and coral bleaching. Field-deployable CRISPR-based biosurveillance enables rapid, frequent, user-driven ocean health monitoring. This can be achieved by detecting gene signatures of barometer species and assessing physiological status through biomarker transcripts. These sensing modalities are integrated into a laboratory-free workflow with low-cost, lightweight 3D-printed devices capable of processing and detecting marine species on-site. (b) Working principle of type V CRISPR-Cas12a diagnostics. Cas12a is guided to dsDNA targets by complementary gRNAs. Target recognition activates nonspecific nuclease activity of Cas12a, which cleaves a fluorophore/quencher-labeled probes (FQ probes, shown in **1**) or FAM/biotin-labeled probes (FB probes, shown in **2**), producing fluorescence or colorimetric LFA readouts, respectively.

The working principle of CRISPR-based nucleic acid biosensing is shown in Fig. 1b, where the Cas12a from *Lachnospiraceae bacterium*^28^ is targeted to double-stranded DNA (dsDNA) targets by programmable guide CRISPR RNAs (gRNAs). Target sequence recognition requires a TTTV protospacer adjacent motif (PAM) site. Any sequence downstream of a PAM site can be targeted by Cas12a by reprogramming the gRNA. Target recognition triggers conformational changes that activate Cas12a’s indiscriminate ssDNA trans-cleavage activity. This activity is quantified using short fluorophore–quencher ssDNA probes (FQ probes), whose fluorescence is restored when Cas12a cleaves them. For field-deployable readouts, we use easy-to-read colorimetric lateral-flow assays (LFAs) with fluorescein–biotin ssDNA probes (FB probes) ^29,30^. Cas12a-mediated cleavage of FB probes produces distinct line patterns on LFA strips, enabling visual detection within 10 minutes. Strips are imaged with a smartphone for quantitative analysis, as detailed in the Methods.

### CRISPR biosensors for rapid *Vibrio* detection

**Figure 2:**
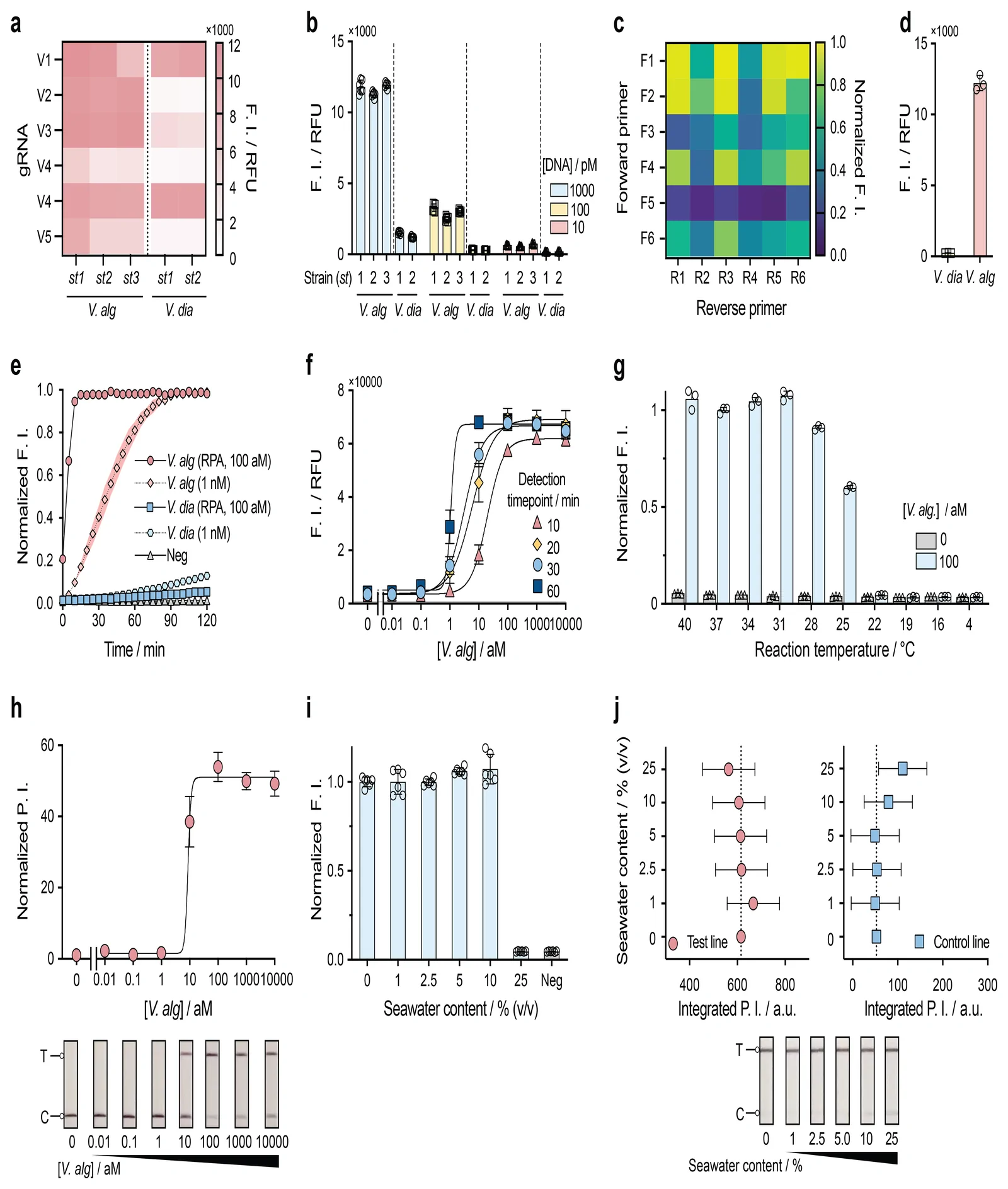
CRISPR-based biosensors for rapid discrimination of pathogenic *Vibrio alginolyticus*. (a) Screening of gRNA designs (V1–V5) targeting *Vibrio alginolyticus* (*V. alg*) thermolabile hemolysin (*tlh*) virulence gene. gRNAs were designed with perfect complementarity to *tlh* regions of three different *V. alg strains* (st), while containing mismatches to homologous regions of *Vibrio diabolicus* (*V. dia*) strains. Heat map shows mean fluorescence intensity (F.I.) from Cas12a reactions with FQ probes, *n* = 6. (b) Species-specific activation of Cas12a guided by *V. alg*-specific gRNA-V2 across a range of target gene concentrations, *n* = 6. (c) Screening of RPA forward (F) and reverse (R) primer pairs. Each RPA amplicon was added to a CRISPR reaction (RPA–CRISPR) with FQ probes, and the primer pair that restored the highest fluorescence intensity (F1–R3) was selected, *n* = 3. Mean intensities at 10 min were normalized to the maximum over 2 h for each primer pair. (d) Comparison of restored fluorescence from optimized *V. alg* biosensor for *tlh* genes (100 aM) of *V. dia* and *V. alg*, demonstrating high specificity, *n* = 3. (e) Performance of *V. alg*. biosensor with RPA-amplified *tlh* gene (100 aM) or non-amplified *tlh* genes (1 nM). Kinetic profiles were normalized to the maximum fluorescence intensity of the RPA-amplified *V. alg tlh* gene, *n* = 3 (RPA-amplified), *n* = 6 (non-amplified). (f) Fluorescence-based dose-response curves for *V. alg* biosensor detecting *tlh* genes at varying Cas12a reaction times, *n* = 6. (g) Performance of *V. alg* biosensor at non-optimal reaction temperatures, *n* = 3. The same temperature was applied to both RPA and CRISPR reactions, and fluorescence intensities were normalized to performance at 37°C. (h) LFA output dose-response curves for *V. alg* biosensor, *n* = 3. Images of LFA strips were captured using a smartphone and processed as described in Methods (Bottom). Test-line pixel intensities (P. I.) were extracted, integrated, and normalized to the negative sample, *n* = 3 (Top). (i) Tolerance of *V. alg* biosensor reactions to seawater content, *n* = 6. Varying volumes of synthetic seawater were included upstream of RPA to simulate residual seawater in samples after filtration, and performance was assessed by FQ probe cleavage. Fluorescence intensities were normalized to 0% (v/v) seawater content. (j) Tolerance of LFA strips to seawater content. Varying volumes of synthetic seawater were included after the completion of RPA–CRISPR, followed by LFA. Images of LFA strips were captured using a smartphone (Bottom) and processed as described in Methods. Test- and control-line pixel intensities (P. I.) were extracted and integrated (Top), *n* = 3. Two-sided one-way ANOVA yielded F statistics of F(5,12) = 1.52 and F(5,12) = 3.61 for test and control lines, respectively, corresponding to p-values of 0.256 and 0.032. Two-sided Dunnett’s post-hoc test revealed no significant differences in the test line from the 0% (v/v) when adding seawater up to 25% (v/v) (all adjusted p > 0.5). The control line showed a significant difference only at 25% (v/v) (p = 0.030, all others p > 0.49). Plotted are the mean signals at different concentrations of seawater, with error bars representing 95% confidence intervals. (a–i) Data represent mean ± s.d. (error bars or lightly shaded region) from independent reactions (*n*), unless otherwise stated.

We generated a gRNA candidate library targeting the *tlh* gene, a critical virulence factor in pathogenic *Vibrio spp*. and host cytotoxicity biomarker^31,32^. We selected *Vibrio alginolyticus* (*V. alg*) as the target species due to its importance as both a medically significant pathogen and a major threat to the aquaculture industry^33,34^. *Vibrio diabolicus* (*V. dia*), the phylogenetically closest species to members of the Harveyi clades (e.g., *V. alg*), was our nontarget negative control^35,36^. The designed gRNA candidates were fully complementary to the target *tlh* gene (*V. alg*) and carried mismatches in the homologous region of nontarget species (*V. dia*) (Supplementary Table S1). Although Cas12a systems are regarded as specific for their programmed targets, for many gRNAs we observed residual activity toward *V. dia* despite the mismatches (Fig. 2a), consistent with prior reports that single-nucleotide resolution is difficult to achieve in practice and highlighting the need for empirical gRNA screening^37,38^. From these screens we selected a final gRNA (V2) for the Cas12a *V. alg* biosensor which produced maximal target signal and minimal off-target activation by *V. dia* (Fig. 2a). The specificity of V2 gRNA was validated under extended incubation, across multiple *V. alg*. and *V. dia*. strains, and over a range of target concentrations up to 1 nM (Fig. 2b and Supplementary Fig. S1).

To boost diagnostic sensitivity and specificity, we incorporated an isothermal RPA target amplification step before the Cas12a detection. We screened 36 pairwise combinations of designed RPA forward (F) and reverse (R) primers. The resulting RPA amplicons were transferred to a CRISPR reaction, and primer pair performance was judged by Cas12a activity (reporter cleavage/fluorescence) after 15 minutes (Fig. 2c). Primer pair F1+R3 demonstrated the strongest activity and was selected for the biosensor. We confirmed the universality of the selected V2 gRNA and RPA primers by analyzing 55 publicly available *V. alg* genomes (Supplementary Fig. S2). The gRNA protospacer and both RPA primer sites were perfectly conserved across all 55 *V. alg* strains, with one exception of a *Vibrio* strain with a point mutation outside the core primer binding region, unlikely to affect amplification). In contrast, all 11 available *V. dia* genomes contained 6 protospacer region mismatches and 5-6 RPA primer hybridization region mismatches.

Combining RPA and CRISPR reactions in a two-step workflow resulted in a *V. alg* biosensor capable of accurate target species discrimination within 40 min (Fig. 2d). Kinetic measurements of Cas reactions confirmed a marked increase in the first-order reaction rate for RPA-enhanced detection of 100 aM (60.2 copies/µL) target, compared to direct detection of non-amplified 1 nM (6.02 × 10^8^ copies/µL) target. Thus, despite a seven-order-of-magnitude lower input target concentration, RPA amplification enables Cas12a-mediated reporter cleavage to reach signal saturation within 10 min (Fig. 2e). Our RPA primers were designed to maximize diagnostic specificity through selective amplification of only desired target species sequences (Supplementary Table S1). Background signals from 100 aM of RPA-amplified non-target *V. dia tlh* genes were lower compared to 1 nM of non-amplified *V. dia* genes, corroborating the specificity of the selected RPA primers. We determined analytical sensitivity using a standard statistical method applied to the biosensor’s dose–response fluorescence curves^39,40^, as detailed in the Methods section. Our results showed that the LOD could be improved with longer reaction times, from 3.39 aM (2.04 copies/µL) at 10 min, 1.18 aM (0.711 copies/µL) at 20 min, 0.662 aM (0.399 copies/µL) at 30 min, 0.761 aM (0.458 copies/µL) at 60 min, to 0.140 aM (0.0843 copies/µL) at 120 min of CRISPR reaction timepoints (Fig. 2f). We selected 30 min as our standard CRISPR reaction time as it robustly produced a sensitive detection signal. We compared biosensor performance under ambient conditions (25°C) to optimal temperatures (39°C for RPA, 37°C for Cas12a). The biosensor maintained ~60% of the optimal signal at 25°C, validating its robustness at lower, ambient temperatures (Fig. 2g). We further optimized the biosensors by exploring the addition of reducing agent to enhance the kinetics (Supplementary Notes and Fig. S3), and probe concentration dynamics for optimal signal production (Supplementary Notes and Fig. S4). For practical field use of our biosensors, we implemented lateral flow readouts by optimizing FB probe concentrations (Supplementary Notes and Figs. S5-6) and developing a custom smartphone-based image analysis pipeline (Supplementary Notes, Methods, and Fig. S3). Image analysis of smartphone-captured LFA strips yielded a detection limit of 6.88 aM (4.14 copies/µL), approximately 10.4-fold higher than that obtained using conventional fluorescence readouts (Fig. 2h).

To assess compatibility with real-world sample matrices, we simulated residual seawater carryover to samples/filters by spiking varying amounts of artificial seawater into the RPA reaction mixtures. (Fig. 2i). There was negligible loss of RPA-Cas12a activity at up to 10% seawater (v/v), but concentrations exceeding 25% (v/v) were detrimental to biosensor performance. We also tested the robustness of the LFA strips to seawater by spiking varying amounts of artificial seawater into completed RPA–CRISPR reactions and assessing LFA development. No statistically significant changes in test-line intensities were observed up to 25% (v/v) artificial seawater compared with a 0% (v/v) spiked-positive control (Fig. 2j). At 25% (v/v), the control line showed a modest but statistically significant reduction in intensity, whereas test-line development remained unaffected. These results confirm that LFA readouts are robust in salt-rich environments.

CRISPR biosensors are modular and easily retargeted to almost any marine organism by gRNA protospacer redesign. Building on our marine pathogen sensors, we adapted the system to detect toxin-producing diatoms that drive HABs. Rising ocean temperatures and anthropogenic eutrophication have increased HAB distribution and frequency worldwide^41^, and their neurotoxins cause major die-offs in marine wildlife and can seriously sicken humans. We focused on *Pseudo-nitzschia australis* (*P. aus*) and *Pseudo-nitzschia seriata* (*P. ser*) – phylogenetically and morphologically similar domoic acid (DA) toxin producing diatoms^42,43^. Low DA-producing *Pseudo-nitzschia pungens* (*P. pun*) and non-DA-producing *Pseudo-nitzschia americana* (*P. ame*) diatoms were selected as control species^44–46^. We targeted internal transcribed spacer (ITS) genes as species-specific barcodes, a common approach for resolving relationships among congeneric taxa^47^.

**Figure 3:**
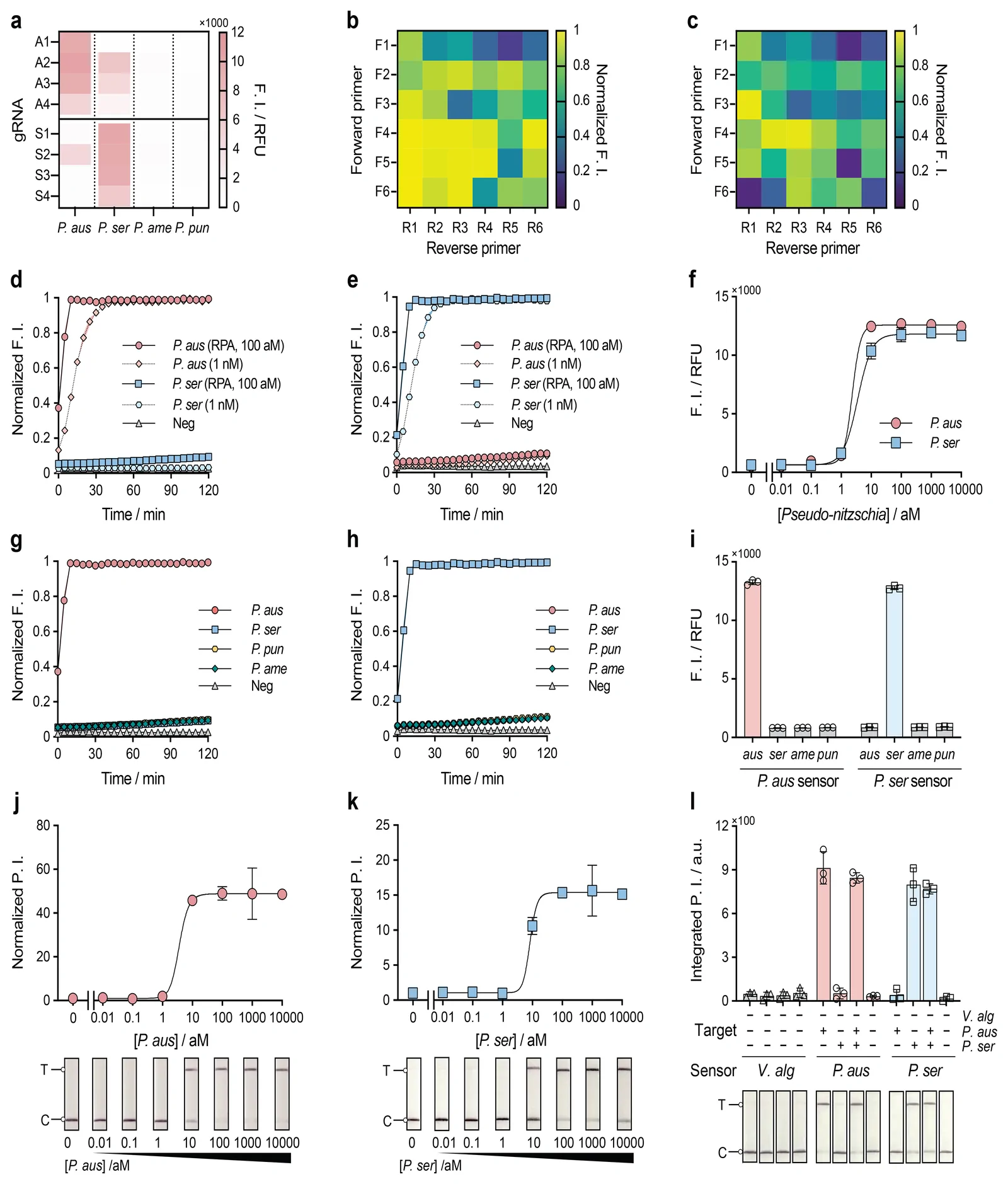
CRISPR-based biosensors for early warning of domoic acid-producing *Pseudo-nitzschia spp*. (a) Screening of two libraries of gRNAs, A1–A4 and S1–S4, targeting internal transcribed spacer (ITS) genes of *P. australis* (*aus*) and *P. seriata* (*ser*), respectively. Heat map shows mean fluorescence intensity (F.I.) from Cas12a reactions screened with FQ probes and gRNAs across species, *n* = 3. Low and non-domoic acid-producing *P. americana* (*ame*) and *P. pungens* (*pun*) served as negative controls. gRNAs producing the strongest species-specific FQ probe cleavage (A1, S1) were selected. (b–c) Screening of RPA forward (F) and reverse (R) primer pairs. Heat map shows mean fluorescence intensities at 10 min, normalized to the maximum over 2 h for each primer pair. Optimal pairs yielding the highest fluorescence output from FQ probes were selected for (b) *P. aus* (F3–R1) and (c) *P. ser* (F4–R3) biosensor, *n* = 3. (d–e) Cas12a-driven fluorescence restoration of FQ probes with RPA-amplified targets (100 aM) or non-amplified targets (1 nM), using (d) *P. aus* biosensor and (e) *P. ser* biosensor, *n* = 3. Fluorescence intensities were normalized to the maximum of the corresponding RPA-amplified targets. (f) Fluorescence dose-response curves for ITS gene detection of *P. aus* and *P. ser, n* = 3. (g–i) Species-specific HAB detection with selected gRNA and RPA primers. Shown are restored fluorescence from FQ probes with 100 aM of ITS genes of the four different HAB species using (g) *P. aus* and (h) *P. ser* biosensors. Fluorescence intensities were normalized to the maximum of the corresponding RPA-amplified targets. (i) Mean fluorescence intensities at 30 min, summarizing diagnostic specificity, *n = 3*. (j–k) LFA-based dose-response curves for ITS detection of (j) *P. aus* and (k) *P. ser*. Images of LFA strips were captured using a smartphone and processed as described in Methods (Bottom). Test-line pixel intensities (P. I.) were extracted, integrated, and normalized to the negative sample, *n* = 3. (l) Parallelized Cas12a detection of amplicons from one-pot multiplexed RPA of ITS genes of *P. aus* and *P. ser* (0 aM or 100 aM) mixed in combinatorial combinations. RPA reactions contained the optimal primer pairs for *P. aus* and *P. ser* for simultaneous amplification. LFA-based detection of distributed amplicons using optimized *V. alg, P. aus*, and *P. ser* gRNAs demonstrates parallelized biosensor operation. Images of LFA strips were captured using a smartphone (Bottom) and processed as described in Methods. Test-line pixel intensities (P. I.) were extracted and integrated (Top), *n* = 3. (a–l) Data represent mean ± s.d. (error bars or lightly shaded region) from independent reactions (*n*), unless otherwise stated.

We screened ITS-targeting gRNAs for species-specific Cas12a activation and selected the top performers, A1 (*P. aus*) and S1 (*P. ser*) (Fig. 3a). We screened 36 RPA primer pairs, evaluating amplicon quality by the fluorescence restored from Cas12a-cleaved FQ probes (Fig. 3b and Fig. 3c), designing gRNAs and RPA primers for selective amplification and target-specific detection (Supplementary Table S1). Cas12a cleavage using RPA demonstrated far superior sensitivity relative to unamplified direct detection, which required targets at 10^7^ greater concentration (Fig. 3d and Fig. 3e). Analytical sensitivities of 0.675 aM (0.406 copies/µL) and 0.905 aM (0.545 copies/µL) were achieved for *P. aus* and *P. ser* biosensors, respectively, based on fluorescence measurements (Fig. 3f). Diagnostic specificity was confirmed, as control *P. pun* and *P. ame* targets did not trigger non-specific reporter cleavage or detectable signals (Fig. 3g–3i). We established LFA-based assays for HAB detection following the *Vibrio* biosensor framework, with higher probe loading yielding improved signal-to-noise (Supplementary Fig. S7). Analytical LODs from dose-response curves of smartphone-analyzed LFA strips were estimated at 1.15 aM (0.693 copies/µL) and 4.12 aM (2.48 copies/µL) (Fig. 3j and Fig. 3k).

We assessed the orthogonality of RPA and Cas12a to support parallel multi-species detection, minimizing the need for repeated sampling and filtration in field settings. A two-pot RPA–Cas12a strategy addresses this challenge by decoupling amplification from detection, introducing modularity into the workflow. We implemented multiplexed RPA using two primer pairs to amplify targets (*P. aus* and *P. ser*) in parallel with combined inputs. Amplicon aliquots were then distributed into separate Cas12a–LFAs with distinct target-specific gRNAs. Results show multiple targets can be co-amplified in one RPA reaction and selectively detected via orthogonal Cas12a assays (Figure 3l). Field applicability was enhanced by a 3D-printed cassette supporting up to four parallel LFA strips (Supplementary Fig. S8). These results demonstrate feasible multi-target detection from a single sample for field-deployable biosurveillance with minimal sample prep.

### CRISPR-based biosensors for monitoring coral heat-stress

To broaden our platform beyond species detection, we investigated CRISPR-based biosensors for monitoring marine organisms’ physiological states. Changes such as heat stress, pathogen infection, or nutrient shifts alter cellular transcription, and CRISPR detection of RNA—either from sampled cells or environmental RNA (eRNA)—can serve as a versatile tool to assess these physiological responses. We focused on coral bleaching, a hallmark of severe coral stress^48,49^. *Porites astreoides*, a widely distributed Caribbean coral^50^, was chosen as our benchmark species due to its recognized suitability as a non-endangered bioindicator^51,52^. Differential regulation of various biomarker transcripts has been observed in *P. astreoides* under thermal stress^53,54^. Heat stress protein 16 (HSP16) was selected as our mRNA target, as it is strongly upregulated in heat stressed corals. We used eukaryotic initiation factor 3, subunit H (EIF3H) as expression reference control.

**Figure 4:**
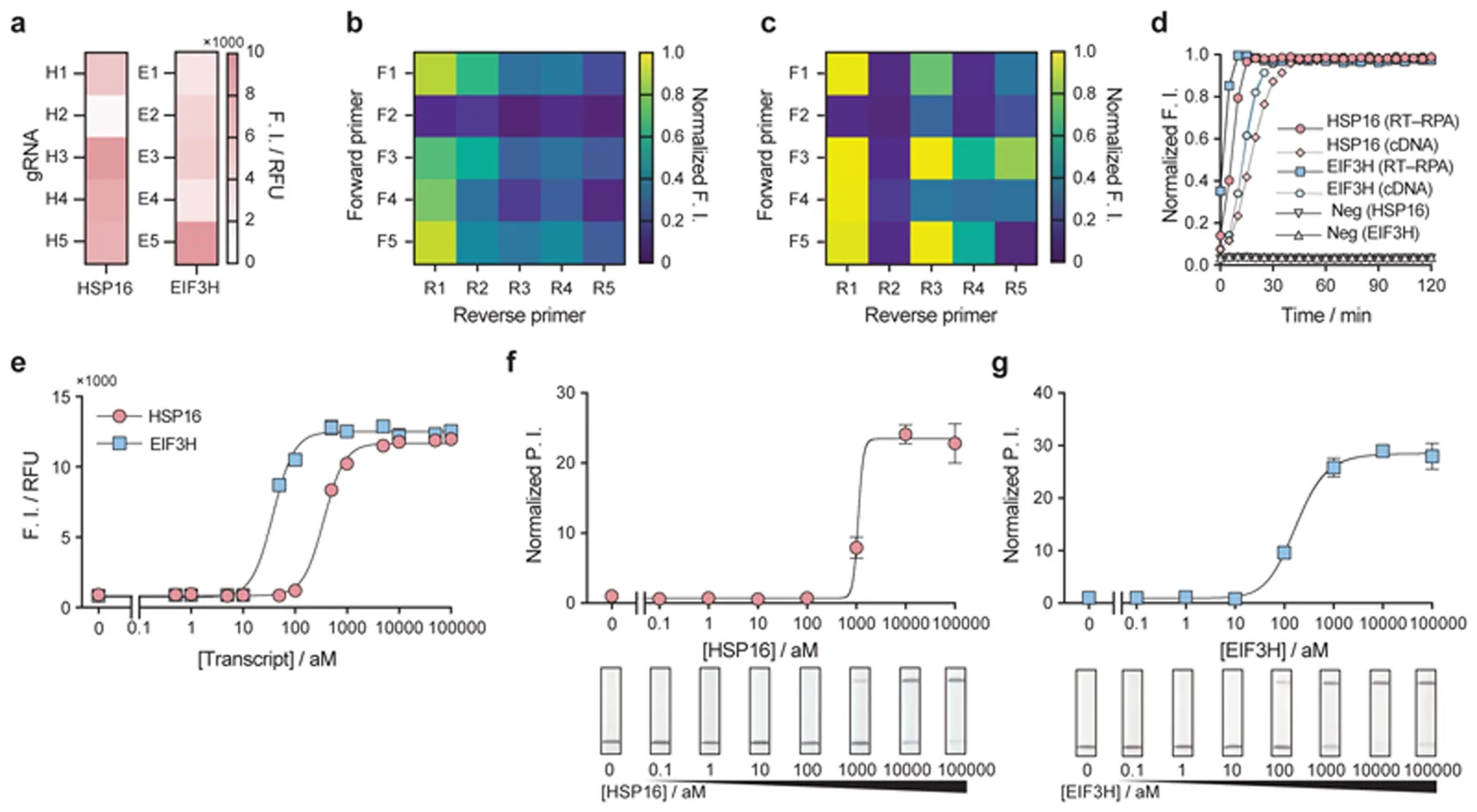
CRISPR-based biosensor for detecting heat-stress coral biomarkers in *Porites astreoides*. (a) Comparison of Cas12a cleavage activities guided by two gRNA (H1–H5 and E1–E5), designed to target cDNA of heat stress protein 16 (HSP16) and eukaryotic translation initiation factor 3 subunit H (EIF3H), respectively. Heat map shows mean fluorescence intensities (F.I.) from the cleavage of FQ probes, *n* = 3. gRNAs triggering the highest FQ cleavage (H3, E5) were selected. (b–c) Screening of forward (F) and reverse (R) primer pairs for one-pot RT–RPA. Each RT–RPA amplicon was added to Cas12a reactions, and primer pairs restoring the highest fluorescence intensity from FQ probes were selected for (b) HSP16 biosensor (F5–R1) and (c) EIF3H biosensor (F3–R1), *n* = 3. Mean intensities at 10 min were normalized to the maximum fluorescence intensity over 2 h for each primer pair. (d) Performance of HSP16 and EIF3H biosensors with RT–RPA-amplified transcripts (10 fM) or synthetic cDNA gene blocks (1 nM). Fluorescence intensities were normalized to maximum intensity of the corresponding RT–RPA-amplified targets. (e) Fluorescence dose-response curves for detection of HSP16 and EIF3H transcripts, *n* = 3. (f–g) LFA-based dose-response curves for detection of (f) HSP16 and (g) EIF3H transcripts. Images of LFA strips were captured using a smartphone and processed as described in Methods (Bottom). Test-line pixel intensities (P. I.) were extracted, integrated, and normalized to the negative sample, *n* = 3 (Top). (a–g) Data represent mean ± s.d. (error bars or lightly shaded region) from independent reactions (*n*), unless otherwise stated.

Five gRNAs targeting HSP16 or EIF3H cDNA were tested, and the one producing the highest FQ probe fluorescence was selected for further study. (Fig. 4a). For these RNA-targeting biosensors, we combined reverse transcription with RPA (RT–RPA) to generate dsDNA from RNA, with the reverse primer serving dual roles as RPA and RT primers; excess reverse primer decreased RT–RPA efficiency (Supplementary Fig. S9). We designed five forward and five reverse primers per target, tested their dsDNA amplicons in Cas12a reactions, and selected the pair yielding the highest fluorescence (Fig. 4b and Fig. 4c). CRISPR reactions using RT–RPA amplicons from 10 fM (6.02 × 10^3^ copies/µL) RNA target inputs exhibited increased kinetics compared to reactions with 1 nM (6.02 × 10^8^ copies/µL) unamplified cDNA templates, confirming successful reverse transcription, cDNA amplification, and CRISPR-based detection of RNA (Fig. 4d). Analytical LODs were determined as in dsDNA gene detection, based on dose-responses from FQ probes in a microplate reader and FB probes (Fig. 4e) on lateral-flow strips (Fig. 4f and Fig. 4g). Estimated LODs from fluorescence measurements were 101 aM (60.8 copies/µL) for HSP16 and 11.3 aM (6.80 copies/µL) for EIF3H transcripts, whereas calculated LODs from lateral flow strips were 880 aM (530 copies/µL) for HSP16 and 63.2 aM (38.1 copies/µL) for EIF3H transcripts.

### Field-deployable platform for in-filter sample processing and CRISPR-based biosensing

**Figure 5:**
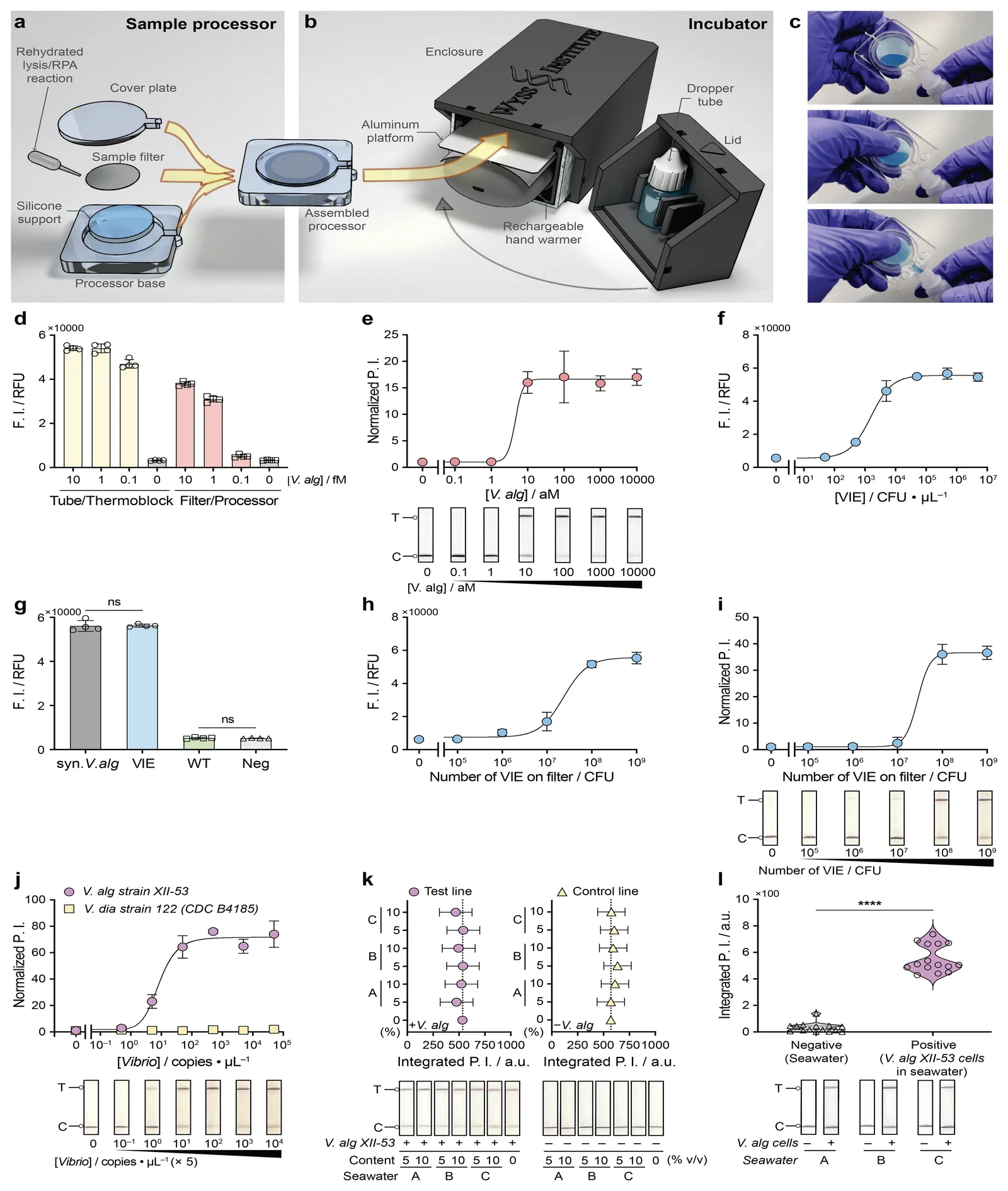
Field-deployable platform with low-cost, portable devices for instrument-free processing and detection of marine species. (a–c) Platform design for a field-deployable biosurveillance system, highlighting device setup and operation: (a) Processor performing in-filter combined lysis and RPA-mediated target amplification. The filter is sandwiched between a cover plate and compressible silicone support. (b) Assembled processor stack inserted into a 3D-printed reusable incubator with a rechargeable hand warmer. The incubator lid holds dropper tubes for subsequent Cas12a detection reactions. Each dropper contains pre-formulated, lyophilized Cas12a reactions rehydrated upon use. (c) Demonstrated collection of the RPA reaction post-incubation by compressing the processor stack to expel fluid, with dye for visualization. (d) Comparison of detection efficiency between laboratory and portable device setups. RPA–CRISPR reactions of *V. alg* biosensor with synthetic DNA targets (laboratory setup) and on filters using our processor-incubator devices (devices), *n* = 4. (e) LFA dose-response curve of *V. alg* biosensor using kit-purified gDNA from *E. coli* (VIE) surrogate cells, *n* = 3. (f) Fluorescence of *V. alg* biosensor for lysis and detection of VIE cells, *n* = 9. VIE cultures were pelleted and resuspended before addition to the lab-based lysis–RPA reaction. (g) *V. alg* biosensors with FQ probe output for detection of synthetic target (830 fM), VIE cells (5 × 10^5^ CFU/μL, equivalent to 830 fM), wild-type *E. coli* BW2113 cells (5 × 10^5^ CFU/μL), and negative control (no cells), *n* = 4. ns (not significant, *p* > 0.05) by ordinary one-way analysis of variance (ANOVA). (h) Fluorescence detection of filter-captured VIE cells using the processor-incubator devices, *n* = 12. Cultures were filtered onto 25 mm 0.8 µm membrane filters, processed with devices, and amplicons collected. Restored fluorescence intensity from FQ probes is shown. (i) LFA detection of VIE cells using the full field-deployable pipeline, *n* = 3. (j) LFA- detection (lab-based) of kit-purified gDNA *V. alg* strain XII-53 vs *V. dia* strain 122 demonstrates biosensor specificity, *n* = 3. (k) Tolerance of LFA strips to natural seawater, *n* = 3. Seawater collected from three geographically distinct sites (Samples A, B, C) were added at 5% or 10% (v/v) to RPA reactions with kit-purified gDNA from *V. alg* strain XII-53 (0 copies/μL negative control; 500 copies/μL (830 aM) positive control), followed by Cas12a detection with LFA readouts. Images of LFA strips were processed as described in Methods. Test- and control-line pixel intensities (P. I.) were extracted and integrated (Top), *n* = 3. Two-sided one-way ANOVA yielded F(6,14) = 0.629 and F(6,14) = 0.565 for test-line (positive sample) and control-line (negative sample) intensities, corresponding to *p*-values of 0.705 and 0.751, respectively. Two-sided Dunnett’s post-hoc test showed no significant differences in both line intensities relative to 0% (v/v) seawater, for both positive and negative target conditions, regardless of sample source or seawater content up to 10% (v/v) (all adjusted p > 0.5). Plotted are mean signals at different concentrations of seawater, with error bars representing 95% CI. (l) Quantified, purified cultures of *V. alg* strain XII-53 (10^8^ CFU) were spiked into three natural seawater samples (A, B, C) and filtered onto 25 mm 0.8 μm membranes. Samples were processed with devices, amplicons collected, and detection performed with lyophilized Cas12a reagents in droppers followed by LFA readouts, *n = 5* independent reactions per seawater sample; total *n = 15* independent reactions. Welch’s unpaired, two-sided t-test showed a highly significant difference between groups, t(17.20) = 18.46, corresponding to a *p*-value of 8.9 × 10^−13^ (****p < 0.0001). Diagnostic performance showed 100% sensitivity (95% CI: 83.2–100%, Wilson method) and 100% specificity (95% CI: 83.2–100%, Wilson method), evaluated using a predefined digital positivity criterion (Methods). (e, i, j) Smartphone images of LFA strips processed as described in Methods (Bottom). Test-line pixel intensities (P. I.) were extracted and normalized to the negative sample, *n* = 3 (Top). (d–l) Data represent mean ± s.d. (error bars) from independent reactions (*n*), unless otherwise stated.

Conventional ocean microbial sampling requires collecting liters of water, filtering the samples, and transportation for lab-based nucleic acid analysis, a process taking hours to days and requiring costly equipment. To enable rapid in-field CRISPR analysis, we developed a portable low-cost (Supplementary Notes, Supplementary Table S2) platform that performs sample lysis and amplification, CRISPR detection, and visual lateral flow readout. For the first step, we engineered a manual disposable processing device for in-filter lysis and RPA amplification of marine samples in a single 30-minute step. (Figure 5a and Supplementary Fig. S10). The device uses a silicone support pad upon which the filter is placed. A lyophilized RPA pellet is rehydrated in a lysis buffer (Supplementary Fig. S11), applied directly to the filter, and a cover plate is placed on top to form a seal as the reaction proceeds. Reaction activity is maximized by an inexpensive 3D-printed insulated incubator enclosure warmed by an off-the-shelf battery-powered hand warmer (Fig. 5b and Supplementary Fig. S12). Each incubator holds two devices, which are removed after a 30-min RPA incubation; amplified nucleic acids are recovered by squeezing the device to compress the silicone support and expel fluid from the filter (Fig. 5c). Subsequent CRISPR-based detection proceeds by rehydrating a lyophilized pellet containing Cas12a/gRNA and probes, followed by mixing 1:1 with the collected RPA in a dropper tube. All fluid volumes are optimized for droppers, allowing simple in-field operation by users. The CRISPR reaction is then placed in the incubator for 30 min for target detection and probe cleavage. Finally, the completed reaction is diluted with buffer and applied dropwise to an LFA strip for visual output development. The full sample processing/preamplification and detection steps are completed within approximately one hour (Supplementary Fig. S13 and Supplementary Fig. S14).

Processing device and incubator performance was evaluated by in-filter amplification of synthetic dsDNA targets using the *V. alg* biosensor (Fig. 5d). While less sensitive than our lab-based assays, the in-filter device enabled effective amplification and consistent detection of dsDNA down to 100 aM (60.2 copies/µL). We engineered a *V. alg tlh* gene-integrated *E. coli* (VIE) as a non-hazardous surrogate to benchmark our portable platform (Supplementary Fig. S15). Purified VIE genomic DNA gave an LFA LOD of 3.14 aM (1.89 copies/µL), consistent with dsDNA target results (Fig. 5e). This confirms VIE as a valid surrogate and demonstrates the biosensor’s high specificity amid complex genomic DNA (Fig. 2h). Next, RPA–CRISPR was performed using VIE cells in lysis–RPA buffers, successfully demonstrating CFU-dependent detection of VIE cells (Fig. 5f, Supplementary Fig. S16 and Supplementary Fig. S17). The analytical LOD was determined to be 3.01 ⨯ 10^2^ CFU per microliter. Similar control experiments using the *V. alg* biosensors against wild-type *E. coli* cells exhibited negligible signal (Fig. 5g).

Performance of our portable device platform for lysing living cell samples and performing in-filter RPA amplification was evaluated using defined amounts of VIE cells applied to 25 mm filters (0.8 μm pore size). Recovered RPA amplicons applied to CRISPR reactions demonstrated that our device enabled effective in-filter single-step lysis and amplification of target genes from VIE cells, with an LOD of 5.00 ⨯ 10^6^ CFU using FQ probes (Fig. 5h). Our full portable pipeline was tested, combining in-filter lysis and RPA of VIE cells, collection of the RPA by manual device compression, subsequent CRISPR detection in the portable incubator with lyophilized reagents, a visual LFA readout step, and smartphone-based imaging and analysis. Since upright dipstick assays are impractical in the field, we optimized probe concentration (Supplementary Fig. S18) and sample dilution (Supplementary Fig. S19) for horizontal LFA use. The portable CRISPR platform detected filter-captured nucleic acids in under an hour without lab equipment, with an LOD of 1.61 × 10^7^ CFU (Fig. 5i) —matching bacterial levels found in ~1 L of seawater during a Vibrio outbreak^55,56^.

### Platform performance of target pathogens in seawater

We extended validation beyond laboratory surrogates and challenged the platform with authentic targets. Pathogenic *V. alg* strain XII-53 and control *V. dia* strain 122 (CDC B4185) were obtained for analysis and the presence of *tlh* verified (Supplementary Figs. S20, S21). Laboratory biosensor tests using extracted genomic DNA showed the similar specificity and sensitivity to the synthetic targets (Supplementary Notes, Figure 5j, Supplementary Figs. S22, S23). We next tested seawater collected from three distinct ocean sites to capture environmental variability in chemistry and biological activity (Supplementary Fig. S24 and Supplementary Table S3). Unfiltered natural seawater was spiked up to 10% (v/v) into RPA reactions with *V. alg* gDNA and tested in our laboratory workflow. Test- and control-line intensities did not differ significantly compared to seawater-free controls regardless of seawater source, demonstrating robust performance to diverse natural matrices (Fig. 5k and Supplementary Fig. S25).

We tested the full field-deployable pipeline by spiking live *V. alg* cells into natural seawater from all three sites, using minimal contamination control. Robust detection at 10^8^ CFU was achieved across all samples (Fig. 5l), comparable to VIE surrogates (Fig. 5i), demonstrating no detectable loss of sensitivity in natural marine matrices. No test line developed in unspiked seawater samples (Fig. 5l). Finally, we benchmarked target release from *V. alg* cells using qPCR with live bacterial input, excluding seawater because its inhibitors disrupt standard qPCR and residual volumes after filtration are not quantifiable for comparison. While qPCR requires denaturation at 95 °C for lysis and nucleic acid release^57^, our portable system achieves in-filter isothermal lysis at 37 °C directly from seawater-filtered samples. By avoiding extreme heating or thermal cycling, our approach removes the need for costly instruments, enabling portable, routine field use (Supplementary Table S4). Thus, qPCR served as a benchmark for target release rather than a direct whole-cell comparator. Using a standard curve from gDNA (Supplementary Fig. S23), qPCR analysis of *V. alg* cultures showed that ~3.4% of target copies were released relative to CFU counts, indicating that the 10^8^ CFU input used in our platform demonstration corresponds to ~3.4 × 106 detectable target copies as measured by qPCR lysis conditions, equivalent to ~28 fM in a 200 µL RPA reaction (Supplementary Fig. S26). These results validate the platform’s performance across ecologically relevant concentrations and highlight its practical advantages over conventional methods, underscoring its value as a rapid, accessible, field-deployable biosurveillance system.

## Discussion

Nucleic acid profiling offers precise, high-resolution assessment of marine species’ presence, abundance, and physiological states. CRISPR–Cas biosensors provide programmable, highly specific, rapid, isothermal detection with versatile readouts^18,58^. Sample preparation often requires harsh lysis, mechanical disruption, or heating and column purification—time-intensive steps dependent on lab infrastructure, hindering on-site marine monitoring. In this work, we addressed this challenge by developing a low-cost, field-deployable CRISPR-based environmental biosurveillance platform for detecting both marine organismal genes and biomarker transcripts. CRISPR biosensors were designed for three representative cases: (1) pathogenic *Vibrio* spp., (2) domoic acid-producing diatoms, and (3) coral heat-stress transcripts. The biosensors were systematically screened for optimal performance and integrated into a field-deployable in-filter lysis and RPA amplification platform. The platform further incorporated lyophilized reagents for stable storage and transport at room temperature, hand-squeezing or drop-based handling for simple on-site operation, and lateral flow readouts for user-friendly visualization. The combined total reaction time for our workflow from sample acquisition to final readout is ~40 min. Collectively, this work establishes a practical pipeline for low-cost, portable CRISPR-based biosensors that integrate sample preparation with rapid, on-site nucleic acid detection without reliance on laboratory infrastructure.

We benchmarked our in-field ocean biomonitoring platform using multiple metrics. Artificial seawater and VIE cells, an engineered *E. coli* surrogate carrying a target sequence, were first used as a biosafe demonstration. The system achieved a sensitivity with an LOD of ~10^7^ CFU VIE cells per filter, which falls within the expected cell population range encountered in 1–10 L of seawater during an HAB or *Vibrio* bacterial outbreak^55,56,59,60^. Validation was extended beyond simulated conditions using natural seawater samples collected from three geographically distinct sites and authentic pathogens. Performance was not compromised when natural seawater was spiked up to 10% (v/v) in RPA reactions, consistent with results obtained using artificial seawater. Robust detection of 10^8^ CFU of live *V. alg* bacteria was achieved when spiked into unfiltered natural seawater across all sites, comparable to the VIE surrogates result and with no apparent loss of detection sensitivity in authentic marine matrices. Unspiked seawater samples produced no detectable signal. These findings show that our platform performs robustly under realistic field conditions with minimal contamination controls and no added purification, highlighting its tolerance to environmental heterogeneity, non-target species, organic matter, and other potential inhibitors (Fig. 5l).

We anticipate that this biosurveillance platform can be extended to diverse marine organisms, where rapid, in-field detection of abundance and physiological shifts would enable timely ecosystem warnings. The current system supports in-filter processing of microorganisms (e.g., bacterial pathogens, diatoms) and shed animal cells (e.g., corals^61,62^). Although authentic diatom and coral samples were not tested here, their cell sizes should allow efficient capture by filtration, and the platform’s modular workflow would enable their analysis with modest adaptations (Supplementary Notes). The platform is equally well suited for environmental DNA/RNA, which has already proven valuable for ecosystem monitoring and biodiversity surveys^63–67^. Nucleic acids adsorbed to cellulose filters during seawater filtration can be directly processed, as we demonstrated with DNA-spiked filters (Fig. 5d). Contamination risks are minimized by single-use processors, sealed vessels, and dropper-based handling. We selected a two-step format over a one-pot approach to maximize efficiency and decouple amplification from detection. This allows a single RPA reaction to support multiple detections, reducing repeated sampling and cross-contamination (Fig. 3l).

Climate change and human pollution are reshaping ocean biomes, underscoring the need for new ecotechnologies to assess their impact^27^. We presented here an on-site, laboratory-free, rapid, and low-cost nucleic acid sensing platform as an alternative to time- and resource-intensive current marine surveillance methods. Although testing ocean samples from active HAB events, *Vibrio* outbreaks, or bleached corals was beyond the scope of this study, the platform proved feasible with environmental and pathogen samples, supporting broader deployment. Continued collaborative efforts will extend the spectrum of detectable organisms and adapt workflows for specific sample processing requirements. Our platform highlights the strength of conventional LFA for rapid and intuitive binary detection. Enhancing platform sensitivity would enable early detection of low-abundance targets. Sensitivity must align with the environmental relevance of each organism, as action thresholds are based on pathogen, sampling location, and season^68^. The current binary-output platform would need target-specific optimization to generate signal only above these baseline levels. Integrating quantitative LFA approaches could enhance resolution and offers a promising direction for future development^69–72^. While this work emphasized high-frequency, field-deployable monitoring of key “barometric” species central to ocean health biosurveillance, the modular design of the platform provides a framework for multiplexed diversity profiling and higher-throughput monitoring^73–75^. While streamlined, our workflow could be improved by reducing user steps and automating precise manual actions, enabling a fully decentralized, on-site process, empowering a wide range of One Health users— ecologists, marine biologists, citizen scientists, conservationists, coastal communities, aquaculture farmers, resource managers, park rangers, and biosecurity officers. A broader usability study evaluating the reliability of end-user operation and the accuracy of result interpretation represents an important next step. Establishing such accessibility would enable large-scale, frequent, user-driven data collection, generating finer-resolution spatiotemporal insights into marine populations and their physiological states. These insights would in turn support early warnings of ecosystem disruptions and emerging threats to public health and industry. Looking forward, we anticipate that the platform described here will help unify One Health collaborative networks by providing a robust foundation for advancing proactive monitoring and sustainable stewardship of ocean health.

## Methods

### CRISPR gRNA and RNA transcript preparation

CRISPR gRNAs or RNA transcripts were either purchased from IDT (used without additional purification) or prepared by performing in vitro transcription. For in vitro transcription, ssDNA templates containing an upstream T7 promoter binding sequence (500 nM) were primer-extended to dsDNA template using NEB Q5 High-Fidelity Master Mix (1×) and a T7 promoter forward primer (500 nM) with a thermal ramp-down from 98°C to 25°C (0.1 °C/s). The dsDNA templates were purified using the Zymo OCC kit, according to the manufacturer’s instructions. In vitro transcription of dsDNA template was then performed using the NEB HiScribe kit, according to the manufacturer’s instructions. Residual dsDNA templates and primers in the reaction mixture (30 μL) were digested by adding DNase I (0.2 U/μL) and thermolabile Exonuclease I (0.4 U/μL) at 37°C for 2 h (final volume 50 μL). EDTA was added to a final concentration of 10 mM, and the mixture was incubated at room temperature (RT) for 10 min to ensure complete chelation of bivalent cations. DNase I and thermolabile Exonuclease I were then heat-inactivated at 80°C for 10 min, followed by RNA purification with the Zymo RCC kit, according to the manufacturer’s instructions. If necessary, to further remove undigested dsDNA template, purified RNA (10 μg) was redigested with DNase I (0.08 U/μL) in DNase I reaction buffer (10 mM Tris-HCl, 2.5 mM MgCl_2_, 0.5 mM CaCl_2_, pH 7.6) at 37°C for 10 min (final volume 100 μL). EDTA was added to 10 mM, and the reaction was incubated at RT for 10 min. Subsequently, DNaseI was heat-inactivated at 75 °C for 10 min, and RNA was purified again using the Zymo RCC kit. Concentration and purity of RNA and DNA were assessed with NanoDrop™ 2000/2000c Spectrophotometers or NanoDrop™ OneC Microvolume UV-Vis Spectrophotometer (Thermo Fisher Scientific, MA, USA).

### CRISPR gRNA and RPA primer screening

CRISPR-based biosensor reactions for gRNA screening were prepared in a final volume of 25 μL containing Cas12a (200 nM), gRNA (300 nM), DTT (5 mM), and ssDNA fluorescence-quenched (FQ) probe (5′ FAM–TTATT–Iowa Black® FQ-3′; 40 nM) and target dsDNA (1 nM) in 1× NEBuffer r2.1 reaction buffer (10 mM Tris-HCl, 50 mM NaCl, 10 mM MgCl_2_, 100 µg/ml Recombinant Albumin, pH 7.9). Fluorescence kinetics were measured at 37°C for 6 h using a BioTek NEO HTS plate reader (BioTek Instruments, VT, USA) with readings every 5 min (excitation: 495 nm; emission: 525 nm). Fluorescence intensities at 120 min were compared, and the gRNA yielding the highest fluorescence intensity was selected for further studies.

RPA primer screening was performed according to manufacturer’s instructions with modifications. A lyophilized RPA pellet was rehydrated with 29.5 μL of Rehydration buffer, supplemented with forward and reverse primer (960 nM each), target dsDNA (100 aM), and MgOAc (14 mM) in a final volume of 50 μL. Reactions were then incubated at 37°C for 30 min in a thermocycler. CRISPR reactions (total volume 25 μL) were prepared by adding 10 μL of RPA reaction mixture, Cas12a (200 nM), gRNA (300 nM), DTT (5 mM), and FQ probe (40 nM) in 1× NEBuffer r2.1 buffer (10 mM Tris-HCl, 50 mM NaCl, 10 mM MgCl_2_,100 µg/ml Recombinant Albumin, pH 7.9). Fluorescence kinetics were measured at 37°C for 2 h using a BioTek NEO HTS plate reader (BioTek Instruments, VT, USA) with readings every 2.5 min (excitation: 495 nm; emission: 525 nm). Typically, fluorescence intensities at 10 min (unless otherwise stated) were compared, and the primer set yielding the highest fluorescence intensity was selected for further studies.

For RT-RPA primer screening, reactions (final volume 50 μL) contained one lyophilized RPA pellet rehydrated with 29.5 μL of rehydration buffer, forward and reverse primer (960 nM each), target RNA (10 fM), MgOAc (14 mM), reverse transcriptase (5 U/μL), RNase inhibitor (1 U/μL) and RNase H (0.25 U/μL). Reactions were incubated at 37°C for 30 min in a thermocycler. CRISPR reactions (final volume 25 μL) were prepared as above, using 10 μL of RPA reaction mixture, Cas12a (200 nM), gRNA (300 nM), DTT (5 mM), and FQ probe (40 nM) in 1× NEBuffer r2.1 buffer (10 mM Tris-HCl, 50 mM NaCl 10 mM, MgCl_2_ 100 µg/ml, Recombinant Albumin, pH 7.9). Fluorescence kinetics were measured at 37°C for 2 h using a BioTek NEO HTS plate reader (BioTek Instruments, VT, USA) with readings every 2.5 min (excitation: 495 nm; emission: 525 nm). Fluorescence intensities at 10 min (unless otherwise stated) were compared, and the primer set that gave the highest fluorescence intensity was selected for further studies.

### RPA–CRISPR reactions with fluorescence and LFA outputs

In a typical RPA–CRISPR biosensor reaction, a lyophilized RPA pellet was rehydrated with 29.5 μL of Rehydration buffer, supplemented with forward and reverse primer (960 nM each), target dsDNA at varying concentrations, MgOAc (14 mM) in a final reaction volume of 50 μL. For RT–RPA, reaction mixture (50 μL) contained one lyophilized RPA pellet rehydrated with 29.5 μL of Rehydration Buffer, forward and reverse primer (960 nM each), target RNA (10 fM), MgOAc (14 mM), reverse transcriptase (5 U/μL), RNase inhibitor (1 U/μL) and RNase H (0.25 U/μL). All RPA and RT–RPA reaction mixtures were incubated at 37°C for 30 min in a thermocycler.

For fluorescence-based CRISPR detection, reactions were prepared in a final volume of 25 μL, containing 10 μL of RPA reaction mixture, Cas12a (200 nM), gRNA (300 nM), DTT (5 mM), and FAM/Iowa Black® FQ-labelled probes (FQ probe; 5′-FAM–TTATT–Iowa Black® FQ-3′; 40 nM) in 1× NEBuffer r2.1 reaction buffer (10 mM Tris-HCl, 50 mM NaCl, 10 mM MgCl_2_, 100 µg/ml recombinant albumin, pH 7.9). Fluorescence kinetics were monitored at 37°C for 2 h using a BioTek NEO HTS plate reader (BioTek Instruments, VT, USA) with readings every 2.5 min (excitation: 495 nm; emission: 525 nm) or using a CLARIOstar Plus Microplate Reader (BMG Labtech, Germany) with readings every 2.5 min (excitation: 483 nm; emission: 530 nm).

For lateral-flow readouts, CRISPR reactions were prepared in a final volume of 25 μL, containing 10 μL of RPA reaction mixture, Cas12a (200 nM), gRNA (300 nM), DTT (5 mM), and FAM/biotin-labeled probe (FB probes; 5′-FAM–TTATT–biotin-3′; 200 nM for dipstick format or 30 nM for flat format) in 1× NEBuffer r2.1 buffer (10 mM Tris-HCl, 50 mM NaCl, 10 mM MgCl_2_, 100 µg/ml recombinant albumin, pH 7.9). Reactions were incubated at 37°C for 30 min in a thermocycler. Lateral flow assays were optimized and performed according to the manufacturer’s instructions (Milenia Biotec). For dipstick assays, 7 μL of RPA–CRISPR reaction mixtures was directly dropped onto the sample application area of a lateral flow strip, incubated at RT for 5 min, and placed upright in 100 μL Milenia chase buffer for 10 min. For flat-format assays, 50 μL of RPA–CRISPR reaction mixture was directly applied to the sample application area of a lateral flow strip and incubated at RT for 10 min. After incubation, test-line development was visually assessed and images were captured using a smartphone (iPhone 14Pro).

### One-pot multiplexed RPA reaction and parallelized detection

For multiplexed RPA, one lyophilized RPA pellet was rehydrated with 29.5 μl rehydration buffer and supplemented with two sets of forward and reverse primers (480 nM each), a mixture of two dsDNA targets in combinatorial combinations, and MgOAc (14 mM) in a final reaction volume of 50 μl. Reactions were incubated at 37 °C for 30 min in a thermocycler. Following amplification, 10 μl of each amplicon was individually added to target-specific Cas12a reactions containing the corresponding gRNA, as described above. Cas12a assays were performed in parallel with LFAs. Non-target biosensors (e.g., *V. alg* biosensor challenged with *P. aus* or *P. ser* one-pot amplicons) served as negative controls. A custom 3D-printed cassette was designed to accommodate multiple LFA strips, enabling parallelized operation.

### Fabrication of processor and incubator devices

The sample processing device was constructed from laser-cut, acrylic plastic (0.093-inch thick; McMaster), which was aligned and laminated using solvent cement (IPS Weld-on). The silicone support for the processor was created from two-part platinum-catalyzed silicone elastomer (Ecoflex 00-30), poured into acrylic molds to a depth where the center of the elastomer had a measured height of 2 mm. The sides of the support adhered to the sides of the mold, forming a concave surface. The incubator enclosure was 3D-printed using FDM printing and PLA filament (Bambu Labs). The heat source for the enclosure was a rechargeable battery-powered 5200mAh hand warmer (Unigear). An insulating sleeve, which slides into the enclosure, was fabricated from reflective bubble insulation to prevent heat loss. To maximize heat transfer between the processor device and the hand warmer, support interface aluminum sheets were cut and assembled using thermally conductive adhesive (Gennel). All 3D modeling files are provided as supplemental files and also uploaded to the NIH 3D Print Exchange portal (https://3d.nih.gov/) under models 3DPX-022943 and 3DPX-022944.

### VIE culturing and processing for lysis/RPA–CRISPR reactions

VIE cells were inoculated in LB media containing Kanamycin (20 μg/ml) and grown overnight at 30°C. Genomic DNA was extracted using the DNeasy Blood & Tissue Kits (Qiagen, Germany) according to the manufacturer’s protocol. For the VIE cell detection assays, potentially autolyzed cells and released DNA due to overgrowth were removed by filtering the culture medium through a bottle-top vacuum filter (0.22 μm) under mild vacuum pressure. The retained VIE cells were then purified by centrifugation (4°C; 2500 rcf; 3 min) and resuspended in fresh LB medium. *E. coli* strain *BW25113* cells (wild-type) were cultured overnight in LB medium without antibiotics and purified using the same protocol as VIE cells.

Optical density (OD) at 600 nm was measured using either a NanoDrop™ OneC Microvolume UV-Vis Spectrophotometer (Thermo Scientific, USA) or a CLARIOstar Plus Microplate Reader (BMG Labtech, Germany) with path-length correction. Calibration curves relating OD_600_ to colony-forming units (CFUs) were generated using standard plating methods. Briefly, overnight cultures were serially diluted and plated on LB agar containing kanamycin (20 μg ml^−1^). Colonies were enumerated from plates with 30–350 colonies.

To perform lysis/RPA reactions of VIE cells in tubes using a thermoblock, defined number of VIE cells were added to the lysis/RPA reaction containing a lyophilized RPA pellet rehydrated with 29.5 μL of Twist Dx Rehydration buffer, forward and reverse primer (960 nM each), MgOAc (14 mM), 1% Triton X-100, and 1% NP-40 in final volume of 50 μL. The fluorescence-based CRISPR reactions were performed as described above. Fluorescence kinetics were monitored at 37°C for 2 h on a CLARIOstar Plus Microplate Reader (BMG Labtech, Germany), with readings taken every 2.5 min (excitation: 483 nm; emission: 530 nm).

### Culture and preparation of authentic *Vibrio* strains

*V. alg* strain *XII-53* (ATCC® 17749™) and *V. dia* strain *122* [CDC B4185] (ATCC® 33840™) were obtained from ATCC and cultured according to ATCC-recommended protocols. Both strains were inoculated in Marine Broth 2216 (BD Difco™ Gibco) and grown at 30 °C for up to 10 h to prevent entry into stationary phase. Marine agar plates were prepared by supplementing Marine Broth 2216 with 1.5% agar.

Calibration curves relating OD_600_ to CFUs were generated from 10 h cultures by serial dilution and plating on Marine agar. Colonies were counted from plates containing 30–350 colonies. OD_600_ measurements were performed using a VWR V-1200 spectrometer.

Colony PCR was performed to confirm the presence of the *tlh* gene in *V. alg* XII-53 using Q5® Hot Start High-Fidelity 2X Master Mix and gene-specific primers (Supplementary Table 1). Amplicons were verified by Sanger sequencing. Genomic DNA was extracted using the DNeasy Blood & Tissue Kit (Qiagen, Germany) following the manufacturer’s protocol. For *V. alg* detection assays, autolyzed cells and free DNA released during overgrowth were removed by centrifugation (4 °C, 2500 rcf, 3 min), and the resulting cell pellets were resuspended in fresh Marine Broth.

### Quantitative PCR (qPCR)

qPCR was performed using the Luna® Universal qPCR Master Mix (New England Biolabs, USA) on a CFX Opus Real-Time PCR System (Bio-Rad, CA, USA). Reaction mixtures contained 1× master mix, 250 nM forward and reverse qPCR primers, and varying copy numbers of genomic DNA (gDNA) extracted from *V. alg* XII-53. Primers were designed to amplify the same region targeted by the RPA amplicons. gDNA copy numbers were estimated from the known genome size of *V. alg* XII-53 and measured DNA concentrations. Amplification plots were generated from serial dilutions over 45 cycles, and standard curves were constructed by plotting the log-transformed input copy number against quantification cycle (Cq) values.

For qPCR on live *V. alg* XII-53 cells, purified and quantified cultures were prepared using OD_600_ºº__ calibration curves. Serial dilutions were made with respect to CFU μl^−1^, and defined numbers of *V. alg* cells (2 μl) were added into 20 μl reaction mixtures.

### Device-based In-filter Lysis/RPA–CRISPR reactions

To perform lysis/RPA–CRISPR reactions on filter-captured samples using the processor and incubator devices (Methods 4.5), defined numbers of cells in 200 μl medium were applied to a 25 mm diameter mixed cellulose ester (MCE) membrane filter (0.8 μm pore size), and residual medium was removed using a bottle-top vacuum filter under mild vacuum. For *live V. alg* XII-53 cell experiments in natural seawater, 10^8^ purified and quantified cells (as determined by OD_600_–CFU calibration) were spiked into three distinct natural seawater samples (see Supplementary Table 3 for sample metadata).

The filters containing captured cells were placed onto the silicone support, followed by the addition of 200 uL lysis/RPA reaction, comprising four lyophilized RPA pellets rehydrated with 118 μL of Twist Dx rehydration Buffer, forward and reverse primers (960 nM each), MgOAc (14 mM), 1 % Triton X-100, and 1% NP-40. For testing of dsDNA on the filter, 50 μL of varying concentration of DNA was directly dropped onto the disc filter placed on the silicon support of the processor. The assembled processor was then placed on the aluminum platform and inserted into the incubator, where it was incubated for 30 min at 37°C using the electronic handwarmer set to 120°F (prewarmed for 5 min; Supplementary Information). Amplicons were collected by compressing the cover plate. Fluorescence-based CRISPR reactions were then performed as described in Methods 4.3. Fluorescence kinetics were monitored at 37°C for 2 h using a CLARIOstar Plus Microplate Reader (BMG Labtech, Germany) with readings every 2.5 min (excitation: 483 nm; emission: 530 nm).

For full platform testing, lyophilized CRISPR biosensor reactions were prepared in a 50 uL volume in a dropper tube, containing NEBuffer r2.1 (1×) buffer (10 mM Tris-HCl, 50 mM NaCl, 10 mM MgCl_2_, 100 µg/ml recombinant albumin, pH 7.9), 5 mM DTT, 600 nM Cas12a, 900 nM gRNA, and 600 nM FB probes, 5% trehalose, 5% pullulan, and 10% mannitol. Reactions were snap-frozen in liquid nitrogen and lyophilized in a Labconco FreeZone 4.5L freeze dryer (Labconco, MO, USA) for at least 24 hrs. To obtain lateral flow readouts, lyophilized pellets were rehydrated with 50 μL (one droplet using the dropper tube) of nuclease-free water and mixed with 50 μL (one droplet using the dropper tube) of collected RPA amplicon from the processor. The dropper tube was then placed into the holder of the incubator and incubated for 30 min at 37°C using the electronic handwarmer set to 120°F (prewarmed for 5 min). Following incubation, 900 μL Milenia chase buffer was added to dilute the reaction. A 50 μL volume (one drop using the dropper tube) of diluted reaction was then applied directly onto the sample application area of a lateral flow strip and run in a horizontal position at RT for 10 min. After incubation, test-line development was visually assessed by eye, and an image was captured using a smartphone (iPhone 14Pro).

## Supplementary Material

Supplemetary Information

## Data Availability

All data supporting the findings of this study are provided in the main text and/or the supplementary materials. Raw data and additional materials are available from the corresponding author upon reasonable request.

## Editor Summary

Human impacts on marine ecosystems are increasing the likelihood of pathogenic outbreaks, harmful algal blooms and coral stress. Authors here develop a CRISPR biomonitoring tool that can help detect key marine species important to public health, the aquaculture sector and marine ecosystems.
