# Supplementary material for: A field‐deployable CRISPR-based biosensing platform for monitoring marine ecosystems
Source: Nat Sustain. Author manuscript; Available in PMC 2026 Aug 24. (PMC7619389; doi:10.1038/s41893-025-01752-0)
Supplement: Supplementary file 1 [file EMS217701-supplement-Supplemetary_Information.pdf]

Table of Contents

Supplementary Notes....2
Supplementary Methods 3
Supplementary Figure S1. Cas12a-triggered activity measured by FQ probes. 5
Supplementary Figure S2. Genome sequence alignment for *V. alg* and *V. dia*. 6
Supplementary Figure S3. Effect of DTT supplementation on Cas12a reactions. 7
Supplementary Figure S4. Numerical modeling of Cas12a kinetics....8
Supplementary Figure S5. Screening of optimal LFA FB probe concentration. 9
Supplementary Figure S6. LFA-based detection of *V.alg* tlh gene....10
Supplementary Figure S7. LFA-based detection of HAB species. 11
Supplementary Figure S8. 3D-printed disposable cassette for parallelized detection....12
Supplementary Figure S9. RT–RPA amplification of *Porites astreoides* HSP16 transcript. 13
Supplementary Figure S10. Processor device operation and fabrication....14
Supplementary Figure S11. RPA–CRISPR performance with varying lysis buffer compositions....15
Supplementary Figure S12. Portable incubator operation and fabrication. 16
Supplementary Figure S13. Sample processing workflow using the field-deployable platform....18
Supplementary Figure S14. Benchmarking of device performance. 20
Supplementary Figure S15. *V. alg* gene-integrated *E. coli* (VIE) strain harboring the tlh gene. 21
Supplementary Figure S16. VIE standard curves. 22
Supplementary Figure S17. Comparison of RPA–CRISPR and Lysis/RPA–CRISPR for VIE detection. 23
Supplementary Figure S18. Screening of optimal FB probe concentration in a flat format. 24
Supplementary Figure S19. Comparison of LFA outputs with varying buffer compositions. 25
Supplementary Figure S20. Standard curves of authentic *V. alg* strain XII-53 cell CFUs. 26
Supplementary Figure S21. Confirmation of the tlh gene in authentic *V. alg* strain XII-53....27
Supplementary Figure S22. Detection of kit-purified gDNA from authentic *V. dia* strain 122 using the *V. alg* biosensor (continued from Figure 5j)....28
Supplementary Figure S23. Quantitative PCR (qPCR) validation of gDNA from *V. alg* strain XII-53....29
Supplementary Figure S24. Map of natural seawater collection sites....30
Supplementary Figure S25. Tolerance of LFA strips to natural seawater. 31
Supplementary Figure S26. qPCR benchmarking of target release from live authentic *V. alg* strain XII-53 cells. 32
Supplementary Table S1. Summary of nucleic acid sequences used in this study....33
Supplementary Table S2. Summary of the estimated costs per sample using the proposed sensor....38
Supplementary Table S3. Metadata for natural seawater samples used in this study. 39
Supplementary Table S4. Technology comparison of nucleic acid detection methods for ocean samples....40
References....41

Supplementary Notes

1. **DMSO Optimization.** Our Cas12a reactions were supplemented with DTT, which accelerated the reaction kinetics and reduced the time to detectable output by ~16 minutes (**Supplementary Fig. S3a**). Although others have noted the potential for reducing agents to degrade Black Hole Quencher dyes[1], we employed Iowa Black as the quencher, which does not appear to suffer from this issue. Even at extended incubations with 5 mM DTT in Cas12a buffer, significant baseline drift was not observed (**Supplementary Fig. S3b**).
2. **Cas12a Probe Concentration Optimization.** Relatively low probe concentrations were used in our Cas12a reactions, yielding sufficient signal outputs in our plate reader setup while conserving reagents. We also conducted numerical modeling of Cas12a reaction kinetics, following the framework previously reported[2]. The analysis indicated that the fraction of cleaved reporter remains constant in the low probe regime. Accordingly, the absolute amount of cleaved product increases proportionally with probe concentration, but the overall increase is modest given the small baseline concentrations. At high reporter concentrations (>10^7), the activated enzyme becomes saturated, and the rate of probe cleavage cannot increase; under these conditions, the proportion of cleaved probe decreases, reflecting redundancy and diminished efficiency (**Supplementary Fig. S4**).
3. **FB Probe Application to LFA Strip Optimization.** Low FB probe concentrations produced incomplete binding to the streptavidin on the control line, allowing excess nanoparticle conjugates to reach the test line and generate false background signals in the absence of target. Excess probes, in contrast, outnumber the available molecular binding sites. We observed decreased control-line intensities and increased test-line intensities under target-negative conditions (**Supplementary Fig. S5**), which is conceptually similar to the 'high-dose hook-effect' in sandwich lateral flow immunoassays[3]. To address this issue, we optimized probe amounts for biosensor performance by testing two different probe amounts (0.14 pmol and 1.4 pmol per strip) within the screened range under target-negative conditions and performed RPA–CRISPR reactions at varying concentrations of the target *V. alg tlh* gene. Higher probe loading (1.4 pmol) provided sharper test lines and improved signal-to-noise ratios without compromising the biosensor sensitivity compared to lower probe loading (**Supplementary Fig. S6**). Higher probe loading produced significantly cleaner test line backgrounds in the absence of target, likely by more effectively retaining residual nanoparticle conjugates at the control line, which otherwise causes faint background signals on commercial LFA strips[4].
4. **Smart-phone Based Digital Signal Analysis.** Despite the minimal background achieved under optimized conditions, naked-eye visual interpretation of LFA outputs remains inherently subjective. To mitigate this, we conservatively defined a positive result under naked-eye inspection as a test-line intensity equal to or greater than the control line after 10 min of lateral flow. Such challenges to LFA detection have been widely recognized, with digital readouts increasingly emphasized as a means to reduce subjectivity and improve field applicability[5,6]. We developed a custom smartphone-based image analysis pipeline (**Supplementary Methods**) that quantifies line intensity. A positive result was defined when the upper 95% confidence interval of the blank was exceeded by the 95% confidence interval of the test sample.
5. **Biosensor Challenge using Authentic Vibrio genomic DNA.** We established CFU calibration curves (**Supplementary Fig. S20**), confirmed the tlh gene by Sanger sequencing (**Supplementary Fig. S21**) and analyzed genomic DNA using the V. alg biosensor with an LFA readout (**Figure 5j**). The analytical LOD was 5.64 aM (3.40 copies/μL), showing high specificity in complex genomic backgrounds and matching results using synthetic dsDNA and VIE gDNA targets. As expected, V. dia gDNA produced no detectable signal, validating assay selectivity (**Fig. 5j** and **Supplementary Fig. S22**). We used quantitative PCR (qPCR) to accurately quantify the

target concentrations; our developed qPCR could detect down to 10 copies per reaction (**Supplementary Fig. S23**). The biosensor LOD for authentic V. alg pathogen gDNA using our sensors was only 6.80-fold higher than qPCR, confirming exceptional assay performance while offering a rapid, simple lab-free workflow with LFA outputs.

6. **Processing of Diatoms and Coral Samples.** More resilient organisms such as diatoms with silicaceous cell walls may require additional disruption steps, for example, using portable bead beaters or sonicators[7-9]. On-site processing could also be achieved for coral biopsy material, if desired, through simple methods such as low-cost homogenizers, syringe–needle shearing, or mortar-and-pestle approaches[10]. Other planktonic cells, detached animal cells, or dissected tissues can likewise be captured by filtration and are expected to remain compatible with minimal modification.
7. **Device Costs.** The processing devices are disposable and with the required RPA and CRISPR lyophilized reagents, the cost per filter sample is only $25.08 (**Supplementary Table S2**). The warmer incubator is reusable, with a total cost of $26.75. The platform costs are expected to decrease with optimized manufacturing and scaling, lowering barriers for the adoption of this ecotechnology for routine use.

Supplementary Methods

Materials

Synthetic gene fragments (gBlocks™ Gene Fragments or gBlocks™ HiFi Gene Fragments), functionalized/non-functionalized DNA oligonucleotides (DNA oligonucleotides or Ultramer™ DNA), and CRISPR-Cas12a guide RNA (gRNA; Alt-R™ L.b. Cas12a gRNA) were purchased from Integrated DNA Technologies (IDT; IA, US) and used without additional purification (**Supplementary Table S1**). EnGen® Lba Cas12a enzymes, RNase-free DNase I, Thermolabile Exonuclease I, Q5® High-Fidelity Master Mix, HiScribe® T7 Quick High Yield RNA Synthesis Kit (HiScribe), Luna® Universal qPCR Master Mix were obtained from New England Biolabs (NEB; MA, USA). Oligo Clean & Concentrator (OCC) and RNA Clean & Concentrator-25 (RCC) kits were purchased from Zymo Research (CA, USA). PrimeScript™ Reverse Transcriptase was obtained from Takara Bio USA (CA, USA) and Protector RNase Inhibitor was purchased from Roche (Switzerland). Invitrogen™ Ambion™ Rnase H (from *E. coli*), dithiothreitol (DTT; 1M), and UltraPure™ 0.5M EDTA (pH 8.0) were obtained from Thermo Fisher Scientific (MA, USA). TwistAmp® Basic recombinase polymerase amplification (RPA) kits were purchased from TwistDX (UK) and Milenia® HybriDetect universal lateral flow dipsticks were purchased from Milenia Biotech GmbH (Germany). Heavy metal-free synthetic seawater was obtained from Ricca Chemical (TX, USA), and formulated ocean sea salt (Instant Ocean Sea Salt) was purchased from Instant Ocean (VA, USA). Marine Broth 2216 (BD Difco™ Gibco) was purchased from VWR (PA, USA). DNeasy Blood & Tissue Kits were purchased from Qiagen (Germany). A Corning® 50 mL tube top vacuum filter system (0.22 µm Pore 13.6cm² cellulose acetate membrane) was purchased from Corning (NY, USA), and MF-Millipore™ mixed cellulose ester (MCE) membrane filters (25 mm diameter disc, 0.8 µm pore size, hydrophilic) were obtained from Milipore Sigma (MA, USA). Thermo Scientific™ Nalgene™ dropper bottles (4 mL capacity, Nalgene™ LDPE Dispensing Tips, 50 uL) were purchased from Thermo Fisher Scientific (MA, USA). *V. alg* strain *XII-53* (ATCC® 17749™) and *V. dia* strain *122* [CDC B4185] (ATCC® 33840™) were purchased from American Type Culture Collection (ATCC, VA, USA). UltraPure™ RNase/DNase-free distilled water (nuclease-free water, Thermo Fisher Scientific, MA, USA) was used for the preparation of all aqueous solution. Corning® Axygen® PCR Tube Strips (Corning, NY, USA), Eppendorf RNA/DNA LoBind® and Protein LoBind® microcentrifuge tubes (Eppendorf US, MA, USA) were used to maximize the sample recovery. For data analysis and curve fitting, GraphPad Prism version 10.3.0 was used.

Genome sequence alignment for *V. alg* and *V. dia*.

As of July 18, 2025, the NCBI RefSeq database contained 55 *V. alg* genomes and 11 *V. dia* genomes, including all strains referenced in this study. All genomes were assembled to the chromosome level, error-checked, and annotated by RefSeq. The *V. alg* gRNA2 (V2) 24-nt protospacer region, the forward RPA primer binding site (*V. alg* F1), and the reverse RPA primer binding site (*V. alg* R3) were assessed across all *V. alg* strains for exact sequence matches. These regions were then compared against *V. dia* genomes to determine the number and genomic locations of mismatches relative to the conserved *V. alg* sequence.

Image analysis and LOD estimation

Images of test strips were captured using a smartphone under uniform lighting and analyzed in Matlab 2023a. Gold nanoparticles have a peak absorption at ~525nm, so the image green channel was used for analysis. For each strip, a region of interest of 16 pixels across the width of the test line (x direction) and 20 pixels along the strip (y direction) was used. When necessary, the y-axis range was expanded to include the whole test line. Averaging across the width of the strip gave an intensity profile, which was normalized to the white region, and numerically integrated to obtain the total signal. The intra-sample variance (the variance across the width of the test line) is much smaller than the inter-sample variance (between test samples), so only the latter was considered.

To estimate the detection limit for each assay, the resulting signals were fitted to a four-parameter logistic regression, as described in Holstein et al. (2015) and Miller et al. (2022), using an open source Matlab application[11-13]. Matlab version R2025a was used. Briefly, the test-line signals are normalized for fitting by dividing by the mean of the negative control signals. A log transform is then applied to the concentrations, and the detection limit is defined as the concentration at which the lower 95% confidence limit of the distribution of a sample at the detection limit intersects with the upper 95% confidence limit of the negative controls (both one-tailed, using the inverse t distribution). For the decision criterion, a positive result was defined when the upper 95% confidence interval of the blank was exceeded by the 95% confidence interval of the test sample. For solution-based assays, the same process was followed, but the fluorescence intensity values were used instead of pixel intensity values.

Although the exponential equation derived in Ramachandran et al. (2021) describes some CRISPR reactions, here the Michaelis–Menten conditions are not satisfied (probe concentration is not much smaller than K_M), so there is no analytical solution to the kinetic equations[2]. This leads to systematic fitting errors when using the exponential. We therefore opted for an empirical 4-parameter logistic regression fit, as we are estimating detection limits, rather than measuring kinetic constants.

Cloning of *V. alg tlh* target-integrated *E. coli* (VIE) strain

A synthetic DNA fragment containing regions 1899 to 2400 bp of the *V. alg tlh* gene was cloned into the pOSIP-KO-186 clonetegration plasmid between the BamHI and SpeI sites using HiFi cloning (NEB). The clonetegration protocol was followed as outlined by St-Pierre et al. – briefly, the plasmid was transformed into *E. coli* strain BW25113 and grown at 37°C to induce integrase expression[14]. Successful integrants were selected on Luria-Bertani (LB) media containing Kanamycin (50 µg/mL) and genomic integration into the *tRNIleY* gene at the phage att186 site was verified by PCR.

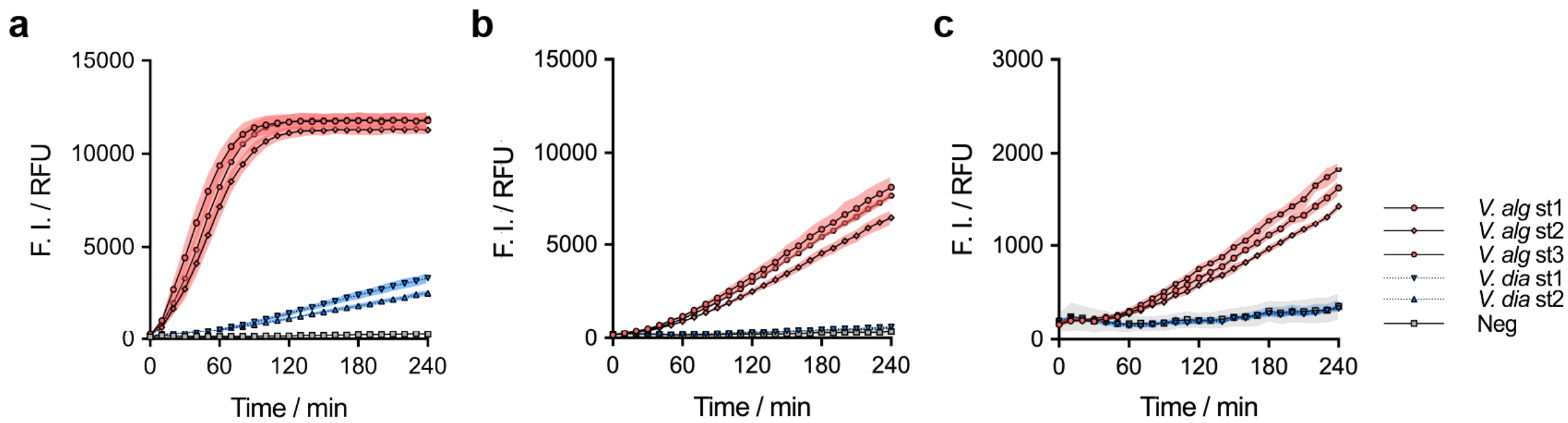

Supplementary Figure S1. Cas12a-triggered activity measured by FQ probes. (a) 1 nM, (b) 100 pM, (c) 10 pM of synthetic *tlh* genes from *V. alg* and *V. dia*. Data represent mean ± s.d. (lightly shaded region) from independent experiments (*n* = 6).

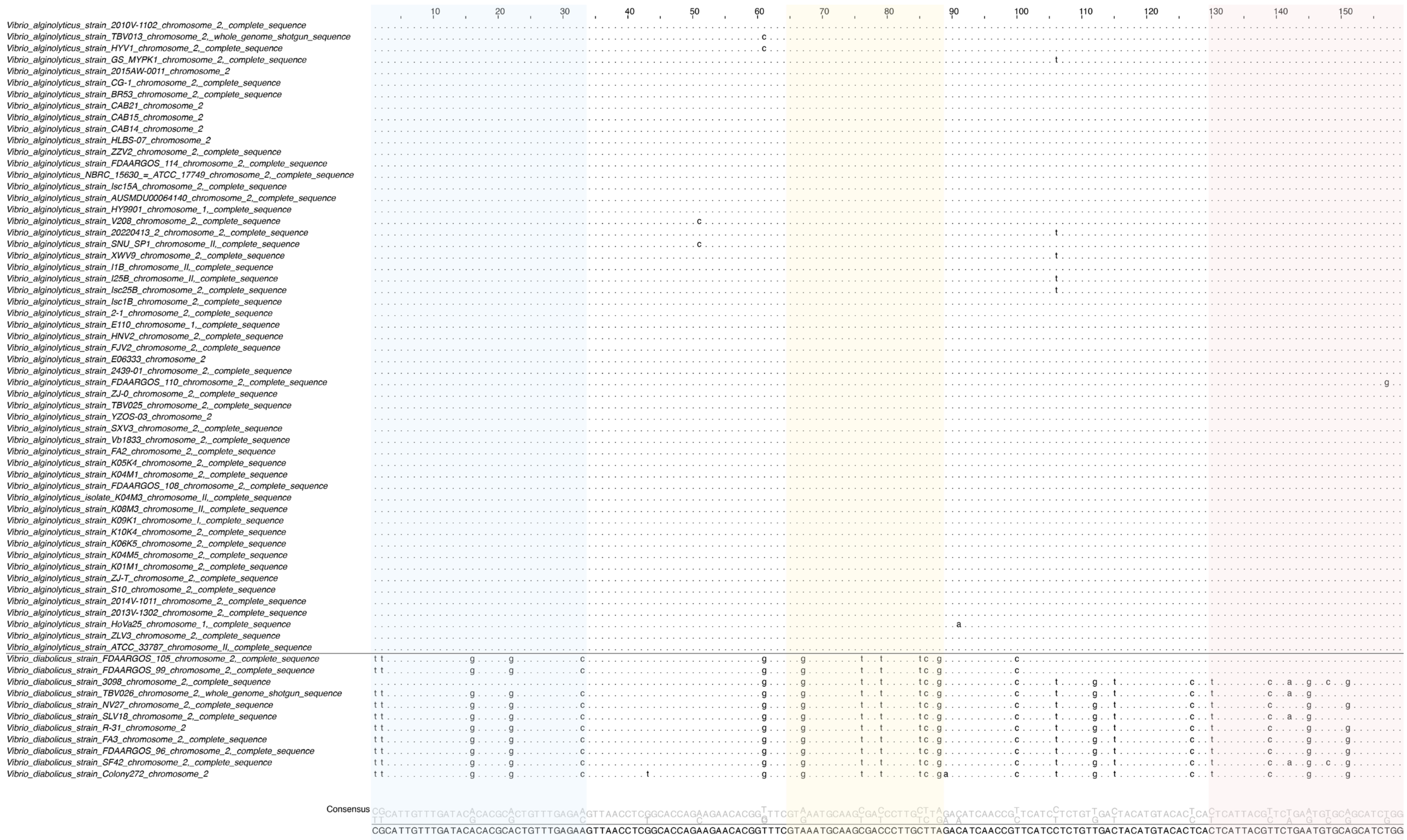

Supplementary Figure S2. Genome sequence alignment for *V. alg* and *V. dia*. 55 *V. alg* genomes and 11 *V. dia* genomes from NCBI were assembled to the chromosome level, error-checked, and annotated by RefSeq. Light blue shading indicates the forward RPA primer *V. alg* F1 binding region, light yellow shading the 24-nt protospacer targeted by the crRNA, and light red shading the reverse RPA primer *V. alg* R3 binding region. Annotated nucleotides mark mismatches. The *V. alg* crRNA2 24-nt protospacer and the forward primer F1 site are exact matches across all 55 *V. alg* strains, including ATCC 17749 (Strain XII-53), which was used as the authentic pathogen in our benchmark experiments. The reverse primer R3 site is an exact match in 54 of 55 strains, including ATCC 17749, with a single T>G substitution at the 5' end in strain FDAARGOS_110 (5′ GGTCTA → 5′ GGGCTA). ATCC 33787 contains a 40-kb gap in chromosome II that disrupts the tlh coding region after amino acid 306 (…QEEIETIRAK__) and the first two nucleotides of codon 307. Because our assay targets the C-terminal region of tlh, the region remains intact downstream of this gap.

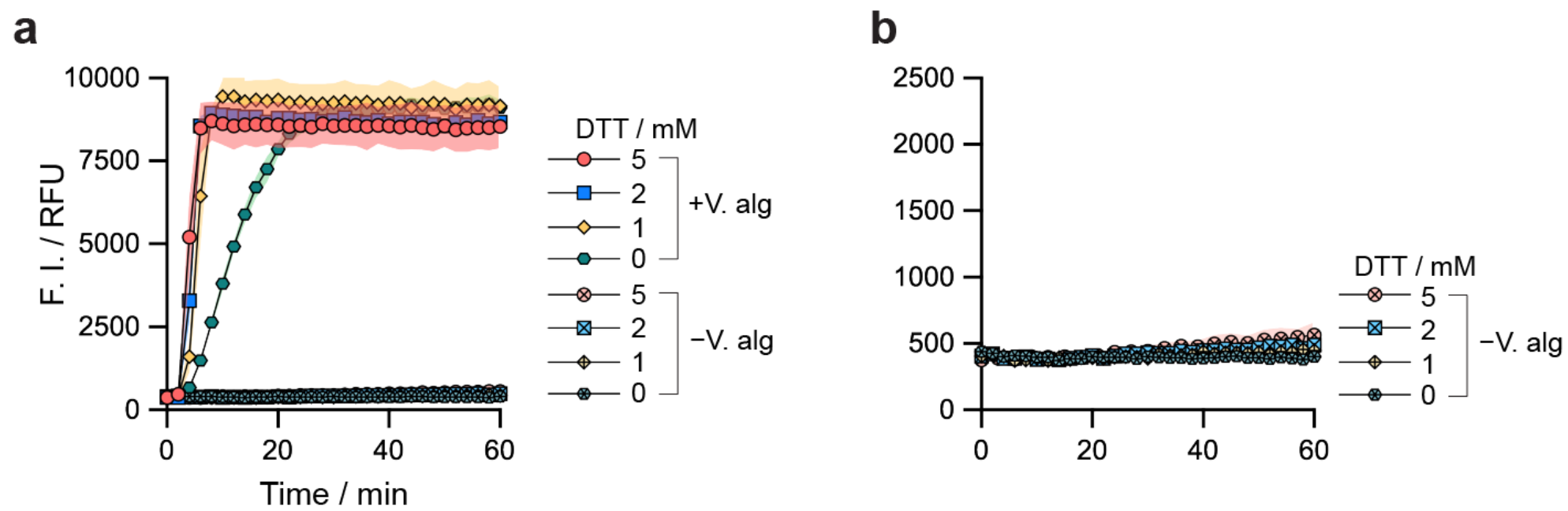

Supplementary Figure S3. Effect of DTT supplementation on Cas12a reactions. (a) Cas12a-driven fluorescence restoration of FQ probes with 0 nM or 30 nM *V. alg tlh* gene, supplemented with varying DTT concentrations (0–5 mM), showing enhanced kinetics. (b) Expanded profiles in the absence of target, confirming no significant baseline drift from DTT supplementation. Data are mean ± s.d. (shaded region) from independent experiments (*n* = 3).

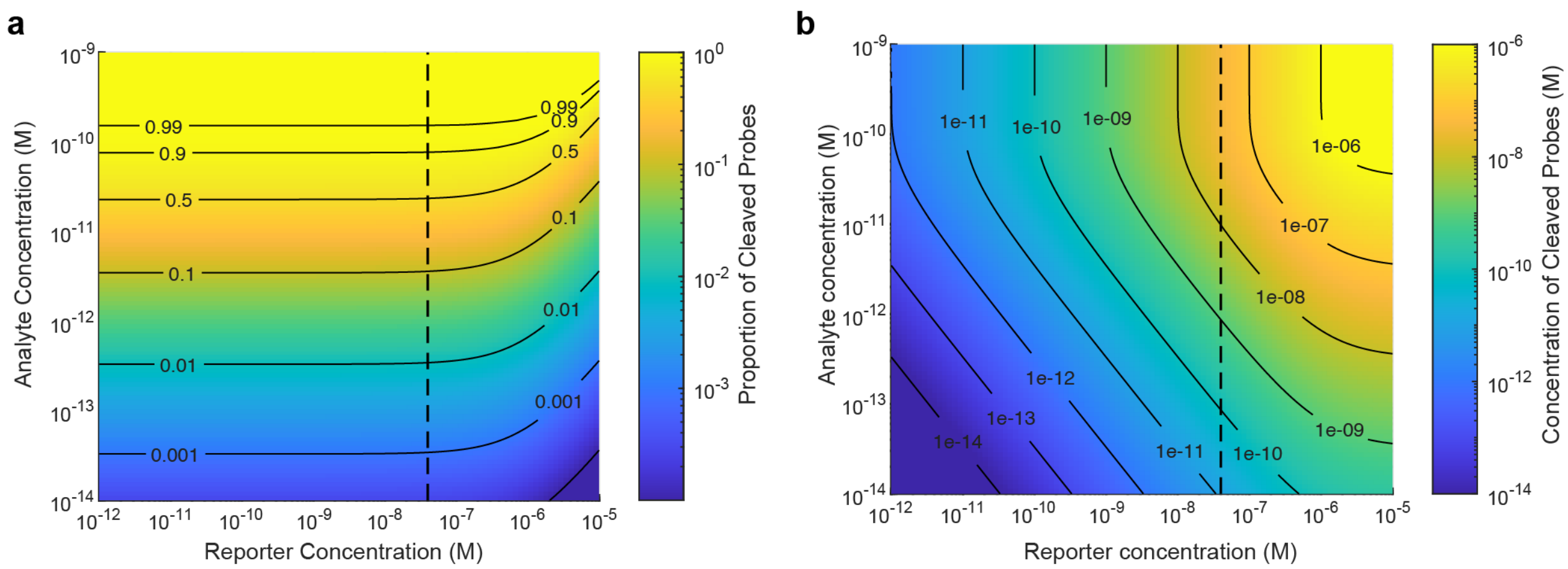

Supplementary Figure S4. Numerical modeling of Cas12a kinetics. Contour plots of (a) proportion of probes cleaved and (b) concentration of cleaved probes across a range of reporter and analyte concentrations. Dashed lines indicate a reporter concentration of 40 nM, as used in this work. Analysis followed the framework of Ramachandran et al. (2021), with results independently replicated in this study.

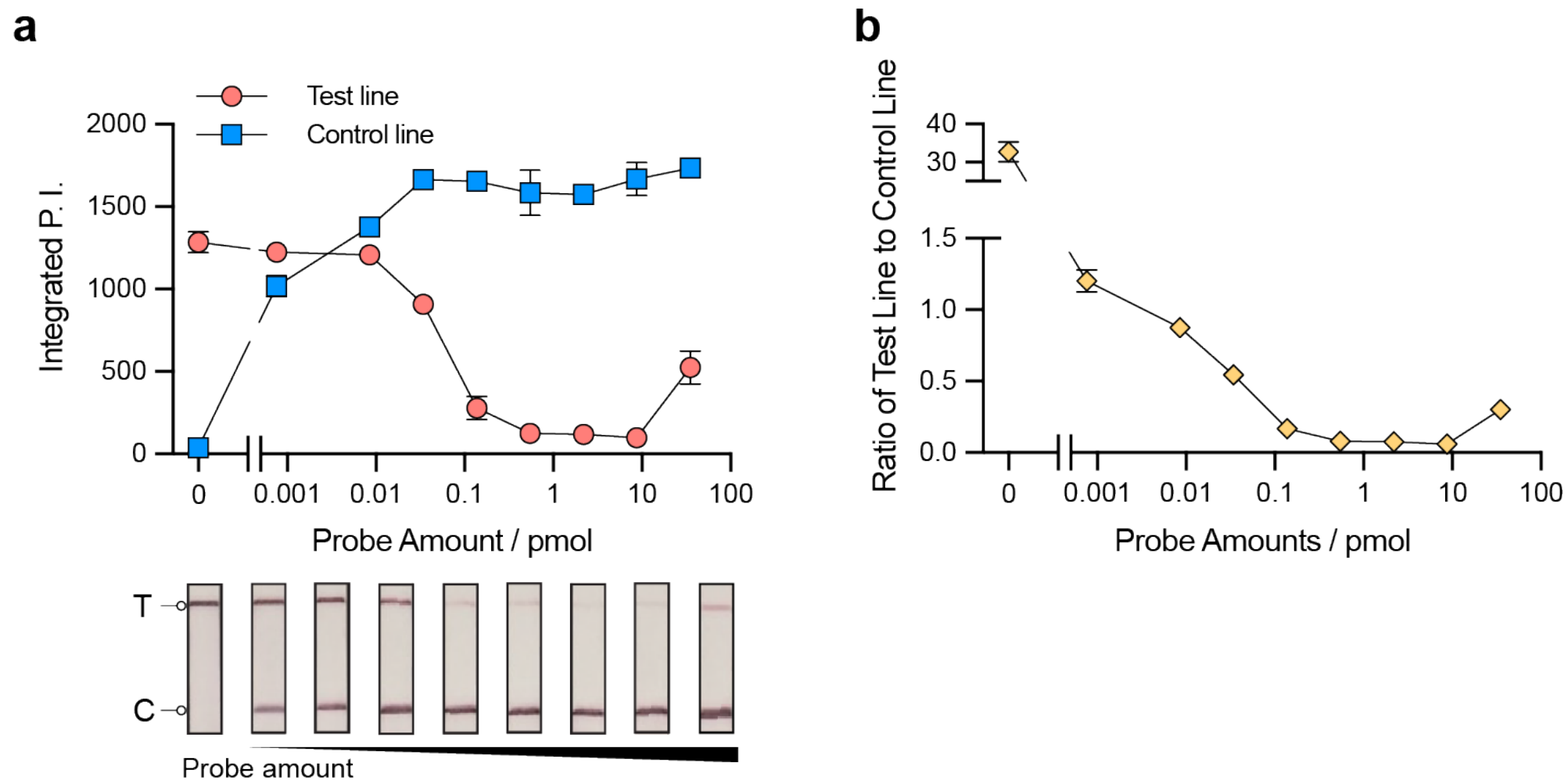

Supplementary Figure S5. Screening of optimal LFA FB probe concentration. (a) Test and control line development with varying probe amounts, using a 7 µL sample volume, n = 2 independent LFA experiments. Images were captured using a smartphone and processed as described in Methods. Error bars represent the combined sample standard deviation (Methods). (b) Ratio of test line to control line mean pixel intensities.

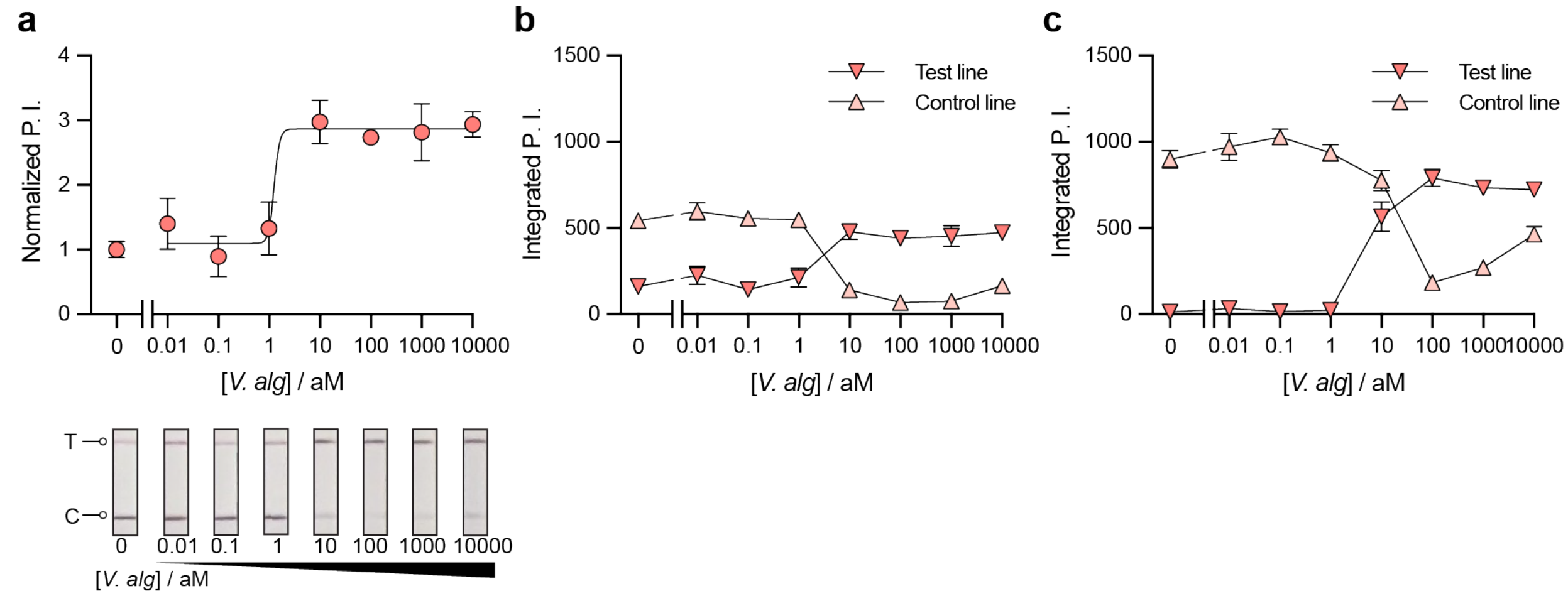

Supplementary Figure S6. LFA-based detection of V.alg tlh gene. (a) Dose-response curves for detection with lower probe amounts (0.14 pmol per strip). Images of LFA strips were captured with a smartphone (bottom) and processed as described in Methods. Test-line pixel intensities (P. I.) were extracted and normalized to the negative sample (top), *n* = 3 independent experiments. Error bars represent the combined sample standard deviation (Methods). (b–c) LFA results at varying *V. alg tlh* concentrations using (b) 0.14 pmol and (c) 1.4 pmol of FB probe per strip.

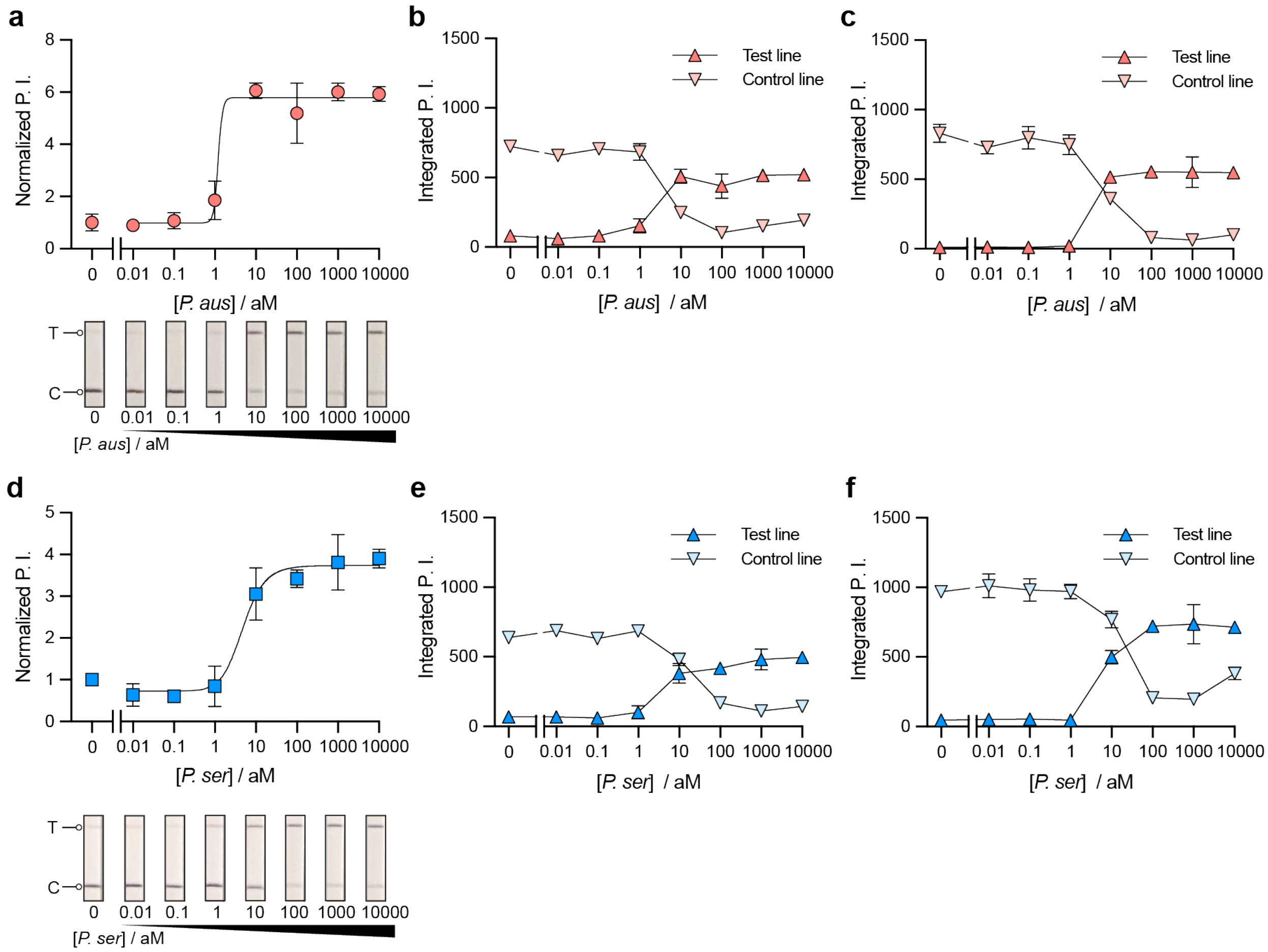

Supplementary Figure S7. LFA-based detection of HAB species. (a) Dose-response curves for detection of *P. aus* ITS gene using lower FB probe amounts (0.14 pmol per strip). (b–c) LFA results at varying *P. aus* ITS gene concentrations with (b) 0.14 pmol and (c) 1.4 pmol FB probe per strip. (d) Dose-response curves for detecting *P. ser* ITS gene using lower probe amounts (0.14 pmol per strip). (e–f) LFA results at varying *P. ser* ITS gene concentrations using (b) 0.14 pmol and (c) 1.4 pmol probes per strip. (a–f) Images of LFA strips were captured using a smartphone and processed as described in Methods. In (a, d), images of test and control lines are shown (bottom). For all data, test-line pixel intensities (P. I.) were extracted and normalized to the negative sample (top), *n* = 3 independent experiments. Error bars represent the combined sample standard deviation, as described in Methods.

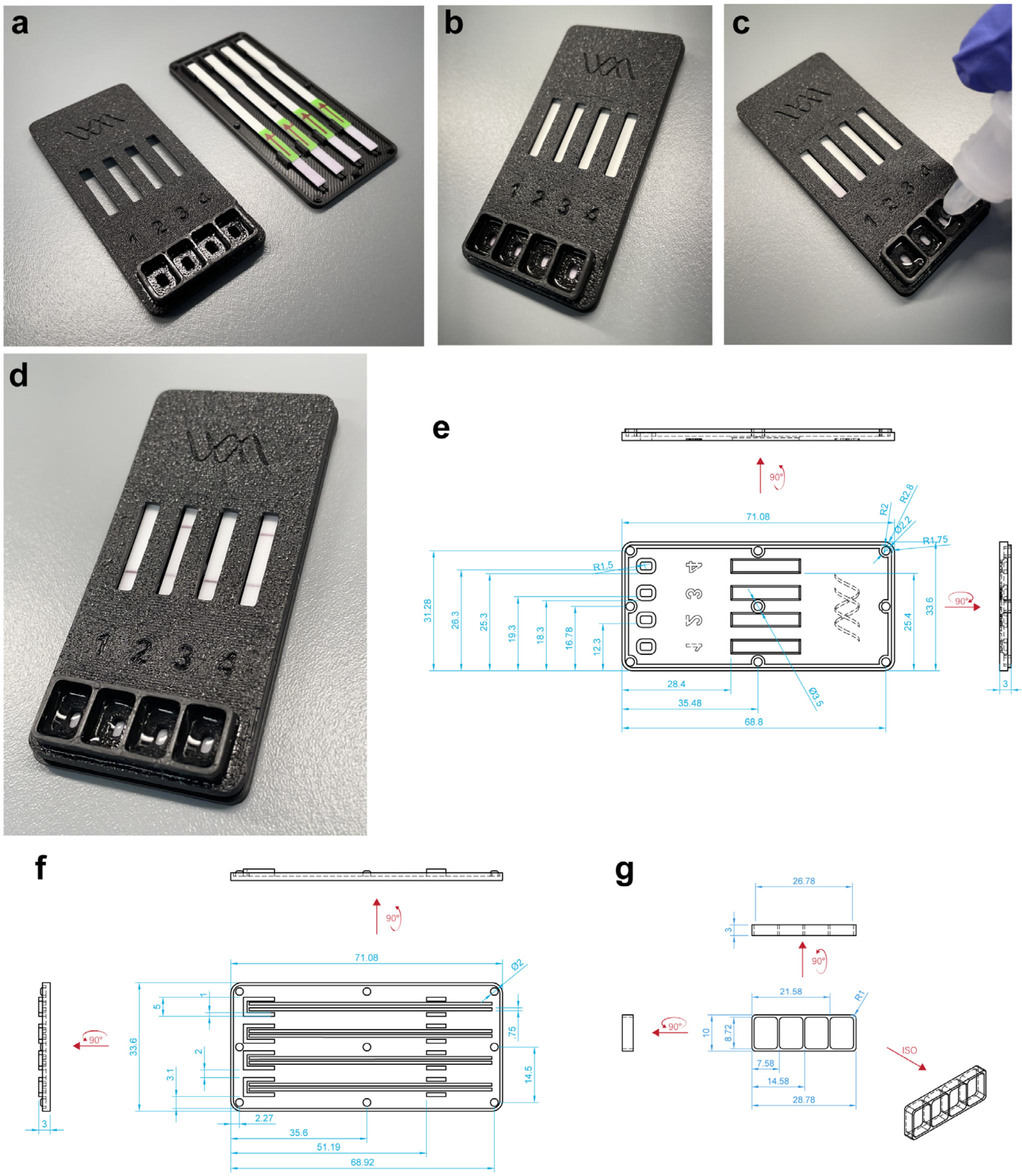

Supplementary Figure S8. 3D-printed disposable cassette for parallelized detection. The cassette holds four LFA strips with individual sample application areas, enabling simultaneous parallel development of four LFAs. One-pot multiplexed RPA amplicons can be distributed into target-specific Cas12a reactions run in parallel, followed by application to the cassette. (a) Unassembled LFA cassette. Well inserts are bonded to the top of the cassette using photopolymer resin. (b) Finished cassette, assembled by simple snap fittings. (c) Loading of wells. (d) Example of parallelized output. Mock reactions from the field procedure of our platform (see Methods) was used for each well. For strips 1 and 3, no Target was added. For strips 2 and 4, 1 nM of final target (*V.alg* tlh amplicon) was added into a mock RPA-lyophilized Cas12a *V.alg* reaction. (e) Schematic of the top half of the LFA cassette for 3D printing. (f) Schematic of the bottom half of the LFA cassette for 3D printing. (g) Schematic of the well inserts of the LFA cassette for 3D printing.

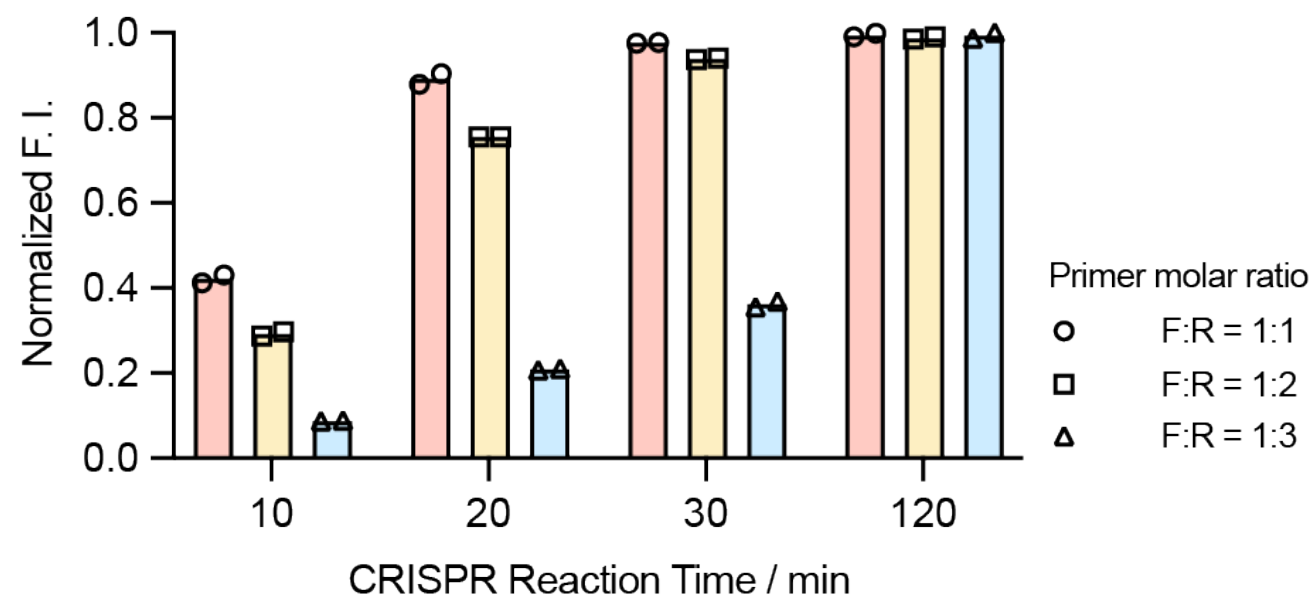

Supplementary Figure S9. RT–RPA amplification of Porites astreoides HSP16 transcript. Amplification efficiency was assessed by Cas12a reactions with FQ probes at different timepoints, across varying molar ratios of forward (F) and reverse (R) primers, *n* = 2 independent experiments.

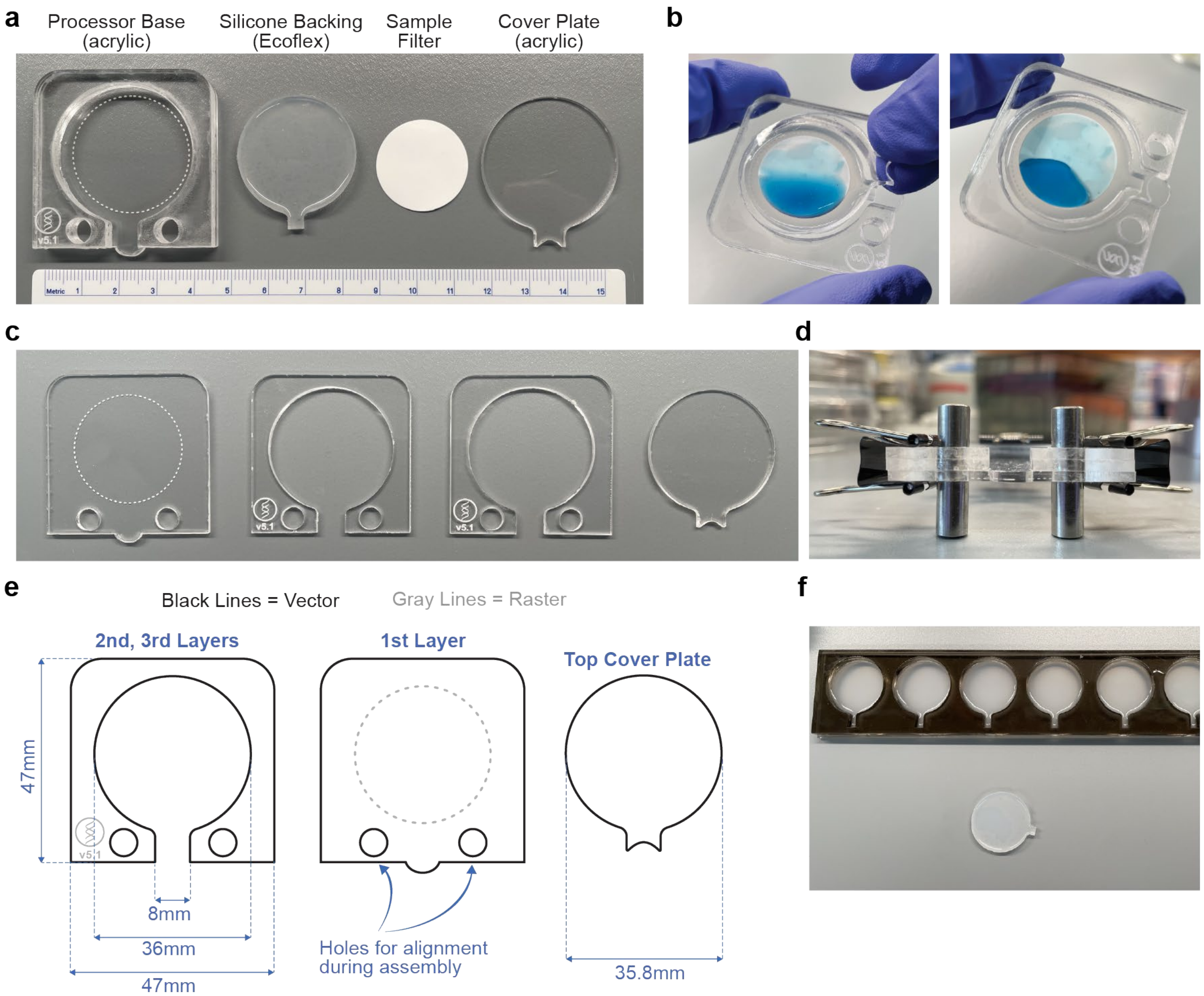

Supplementary Figure S10. Processor device operation and fabrication. (a) The processor consists of four parts, stacked on top of each other. Rehydrated RPA reaction is applied to the sample filter before placing the cover plate on top. (b) Left, placement of the cover plate over the processor stack. Right, fully assembled processor stack ready for incubation. Blue-dyed water used here for visualization. (c) Fabrication of the processor base from three stacked laser-cut acrylic layers. The cover plate is shown on the right. Acrylic thickness was 0.09375 inches. (d) Stacked plates being solvent welded together. Metal rods are for alignment purposes. (e) Dimensions of processor base layers and cover plate. (f) Silicone backing pads fabricated by pouring Ecoflex rubber silicone into an acrylic mold to a center depth of 2 mm. Raised edges are naturally formed by adhesion to the mold walls.

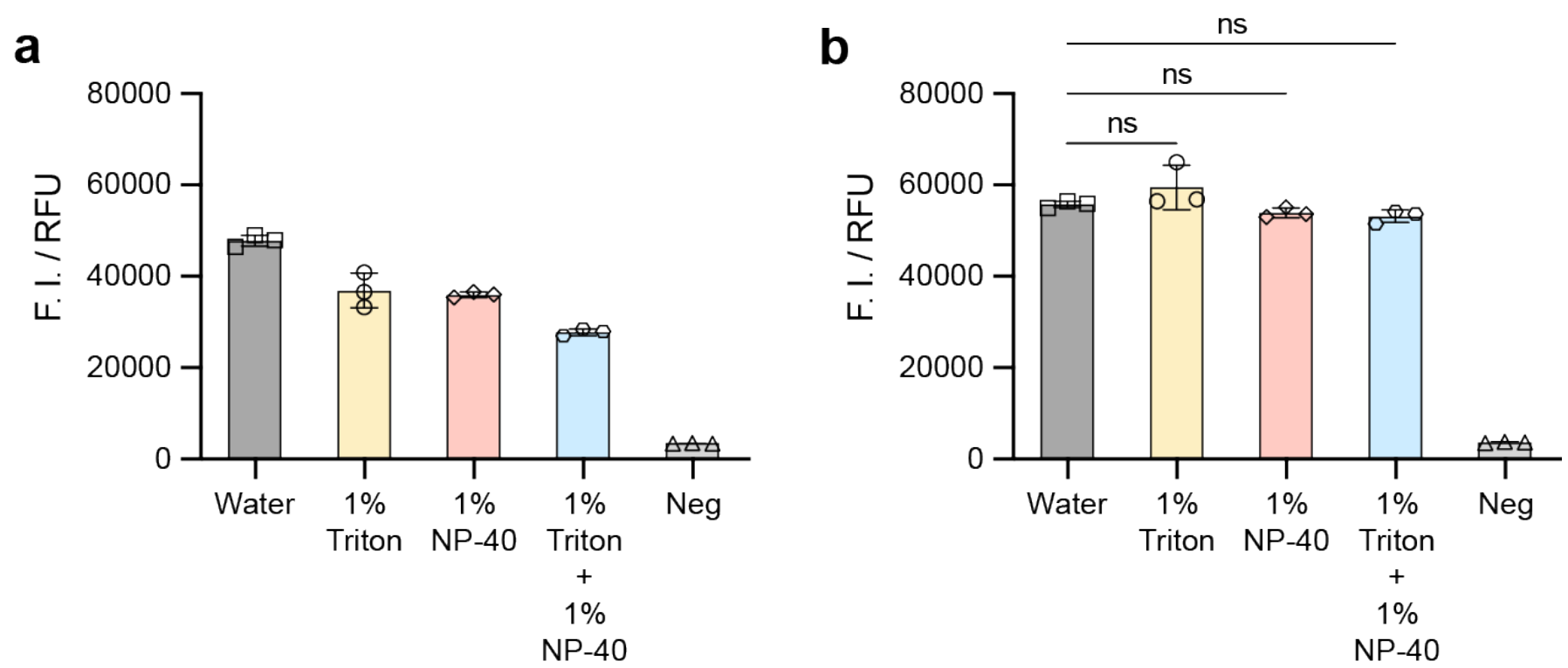

Supplementary Figure S11. RPA–CRISPR performance with varying lysis buffer compositions. Fluorescence intensities at (a) 10 min and (b) 30 min time points, *n* = 3 independent experiments. No significant reduction in performance was observed between RPA–CRISPR and Lysis/RPA–CRISPR after 30 min. n.s. (not significant, $p > 0.05$) based on ordinary one-way analysis of variance (ANOVA).

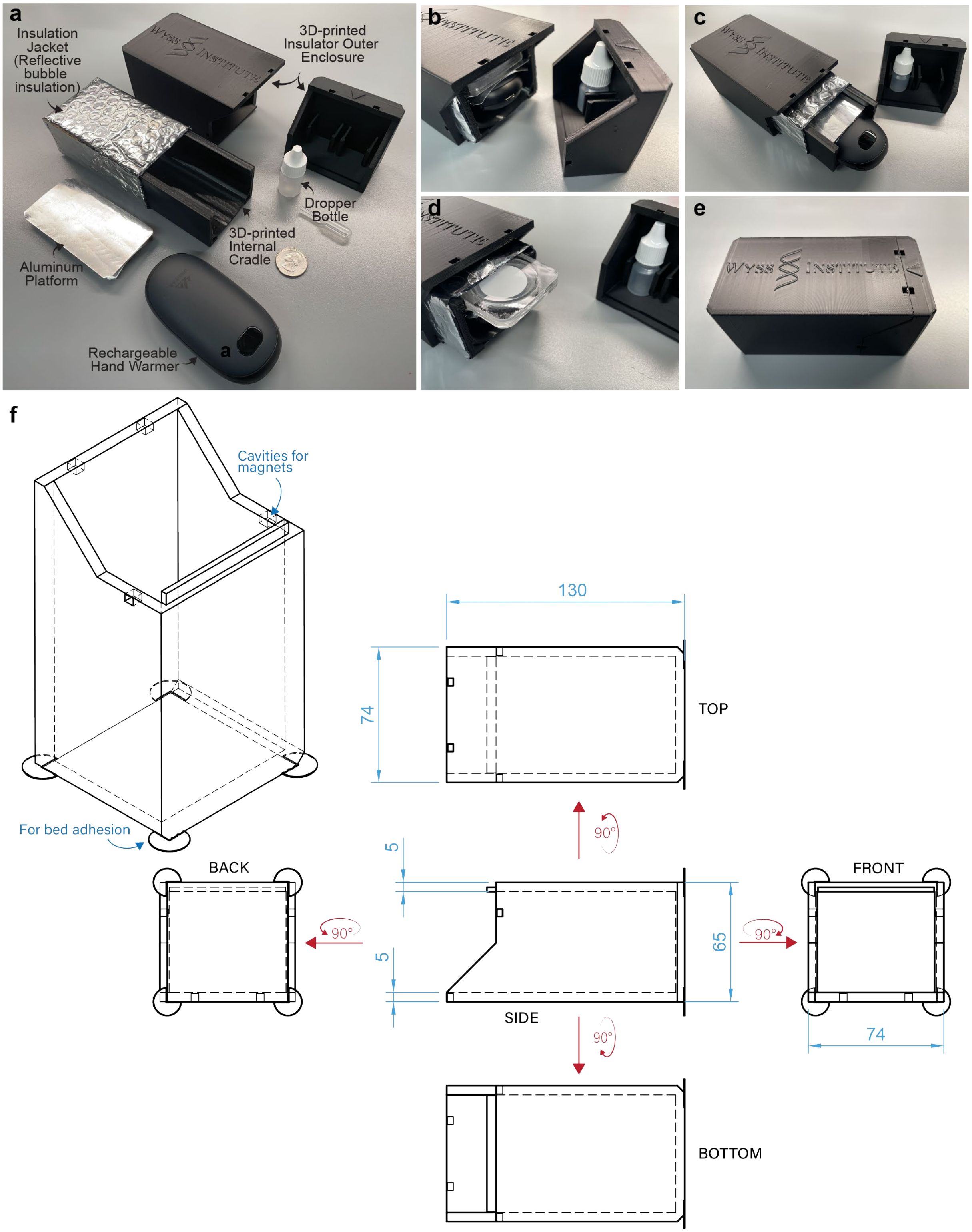

Supplementary Figure S12. Portable incubator operation and fabrication. (a) Disassembled incubator parts. (b) Assembled incubator. (c) Insertion of processor stack into the incubator compartment. (d) Incubator lid with two slots for dropper bottles, in which the RPA-Cas12a reactions will be incubated. (e) Closed incubator. (f) Schematic of the insulator outer enclosure body. Dimensions are given in millimeters. Corner pads shown in the model are for printer bed adhesion and removed after printing.

g

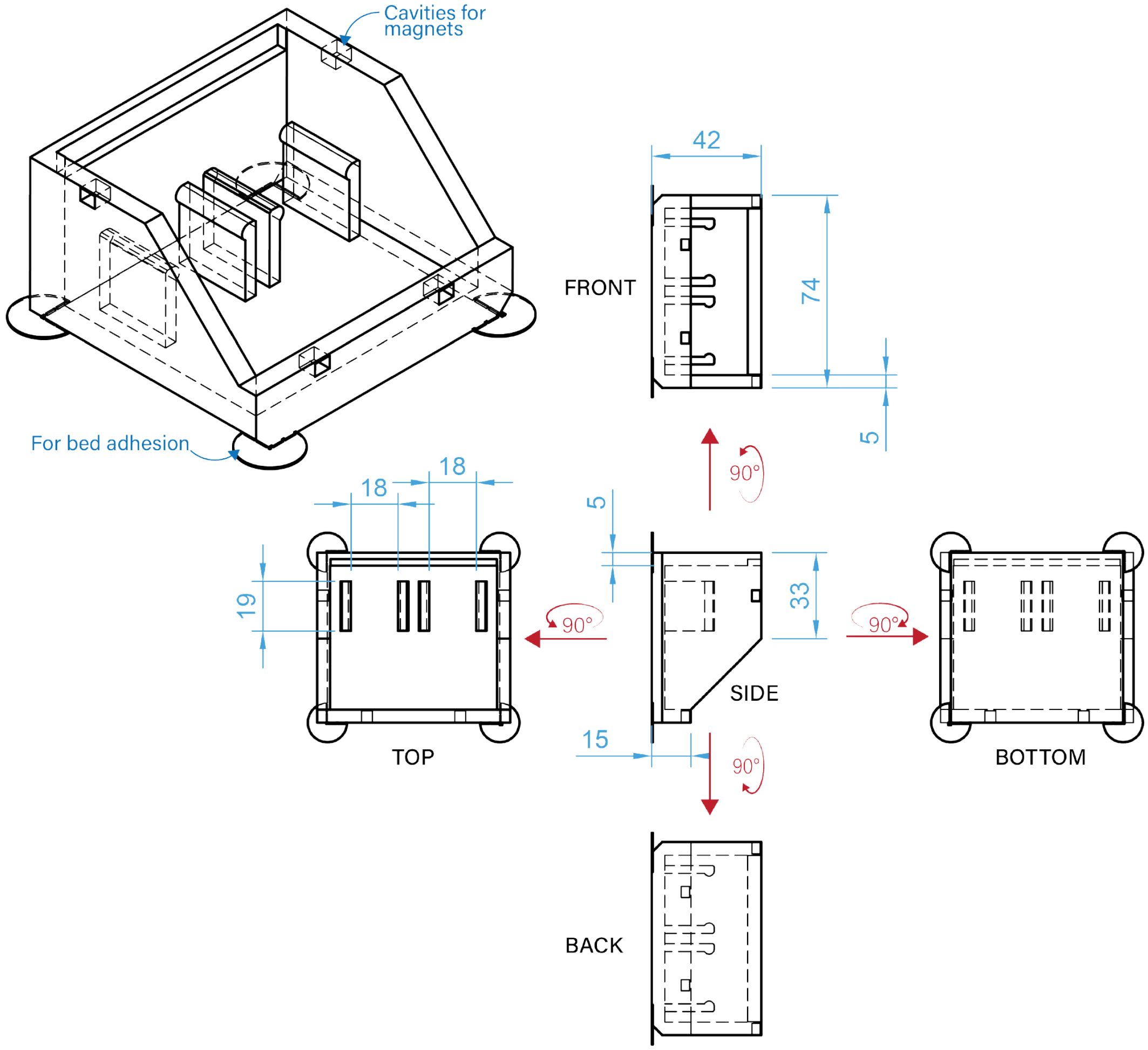

h

3D Printer	Bambu Labs X1C
Filament	Bambu Labs Basic Black PLA (10101)
Nozzle	0.4 mm

Supplementary Figure S12 (continued). Portable incubator operation and fabrication. (g) Schematic of the insulator outer enclosure lid. (h) 3D printer parameters for the incubator fabrication. Dimensions are given in millimeters. Corner pads shown in the model are for printer bed adhesion and removed after printing.

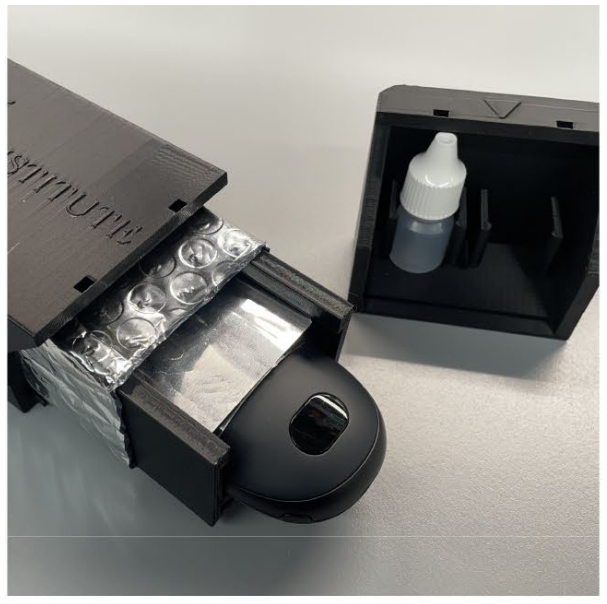

Step 1 - Turn on hand warmer, close incubator to prewarm while preparing the processor.

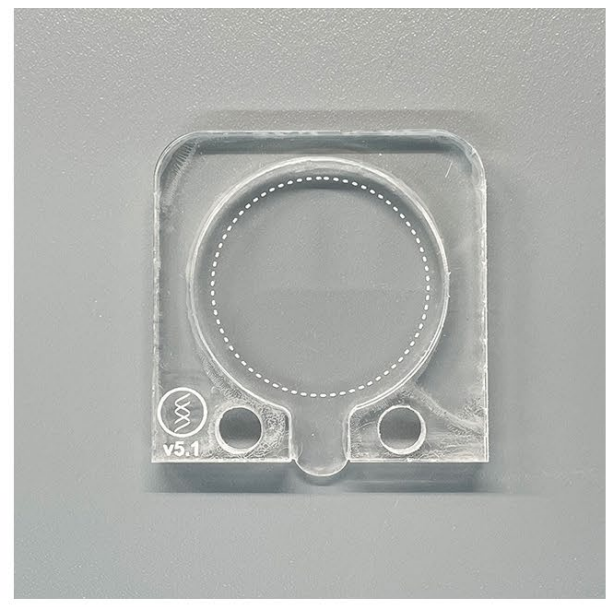

Step 2 - Place processor backing plate on a flat surface.

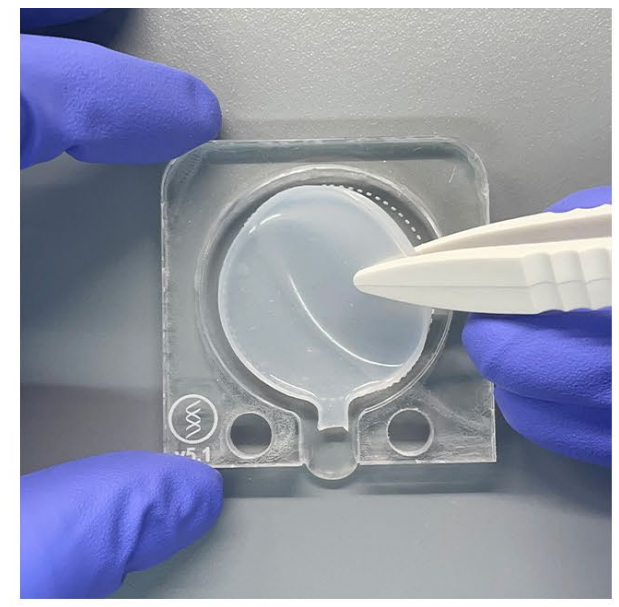

Step 3 - Using forceps, place silicone support into device, following the dotted outline.

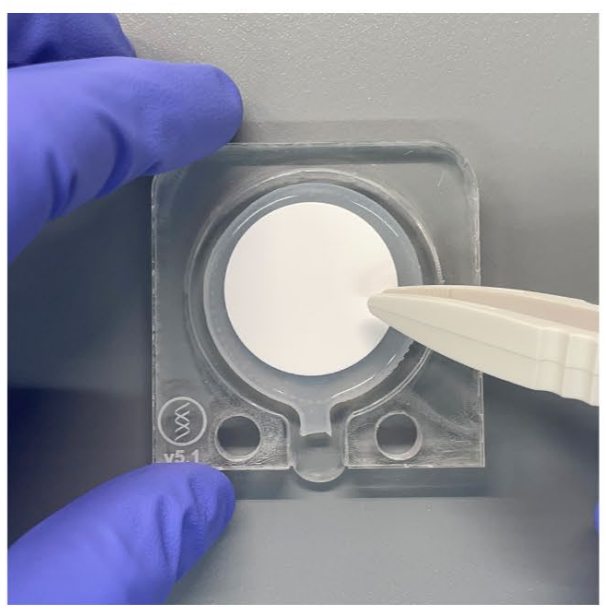

Step 4 - Using forceps, place filter with sample on the center of the silicone support.

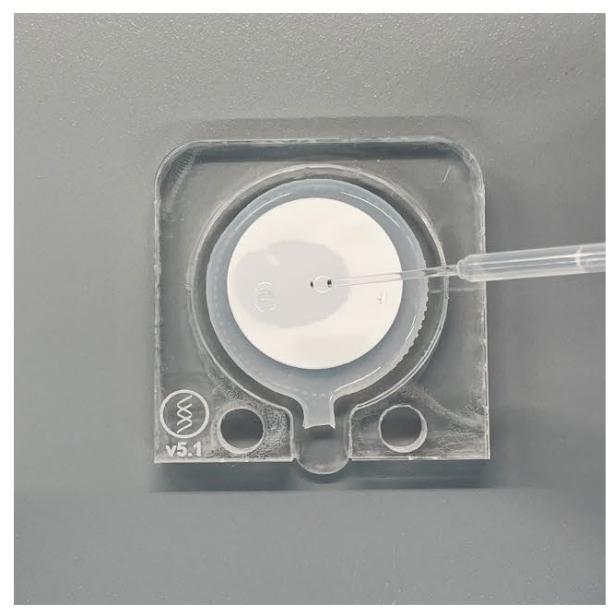

Step 5 - Using dropper, add all of the rehydrated RPA reaction to the filter.

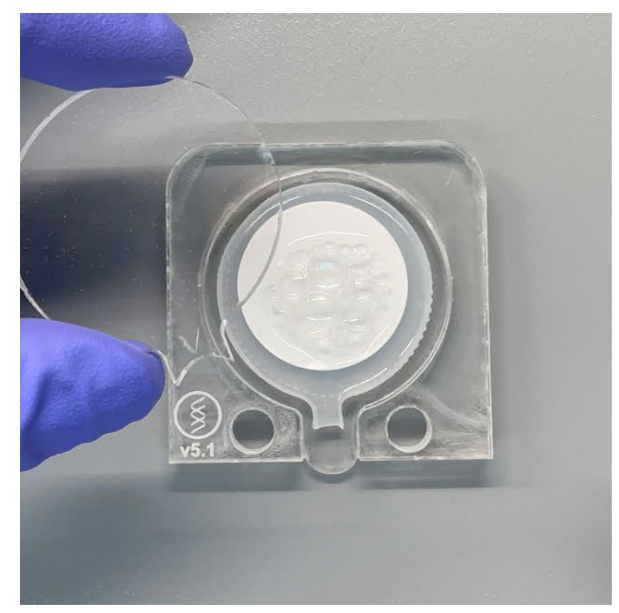

Step 6 - Place processor cover plate over the filter.

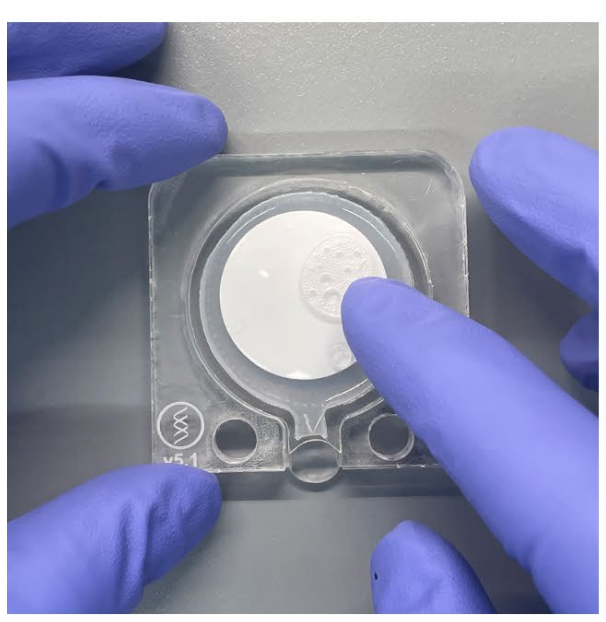

Step 7 - Gently ensure that the cover plate is securely in place over the filter + support. **Do not press down.**

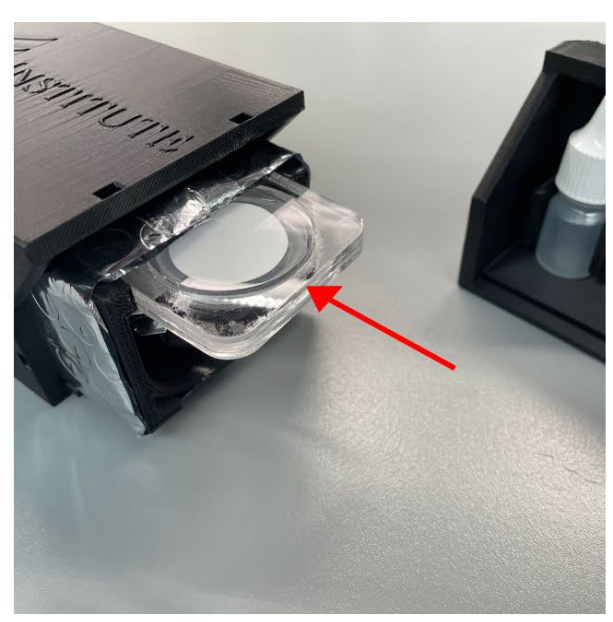

Step 8 - Carefully place device in incubator, secure the lid, and incubate for 30 minutes.

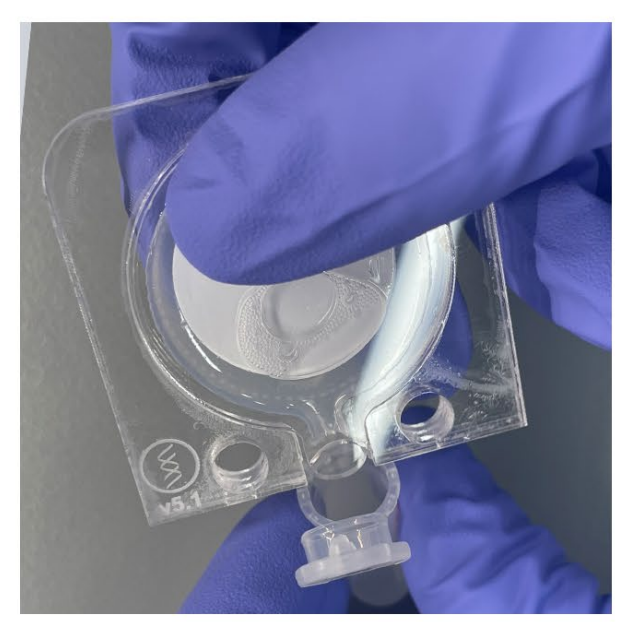

Step 9 - When complete, remove processor from incubator and align output tab with open tube.

Supplementary Figure S13. Sample processing workflow using the field-deployable platform. The workflow illustrates stepwise operation: assembling the processor, performing in-filter lysis/RPA in the incubator, collecting amplicons, performing Cas12a reactions with rehydrated reagents in dropper bottles, and developing LFA strips.

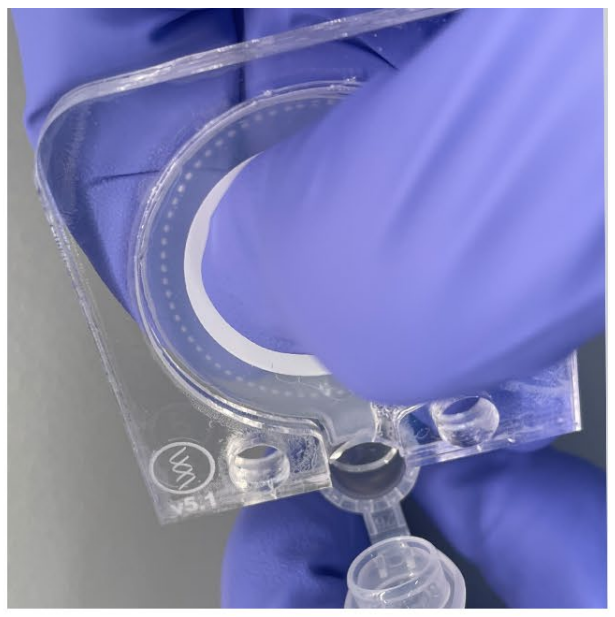

Step 10 - Firmly press down on the cover plate, starting at the top of the device, to eject the RPA reaction into the tube.

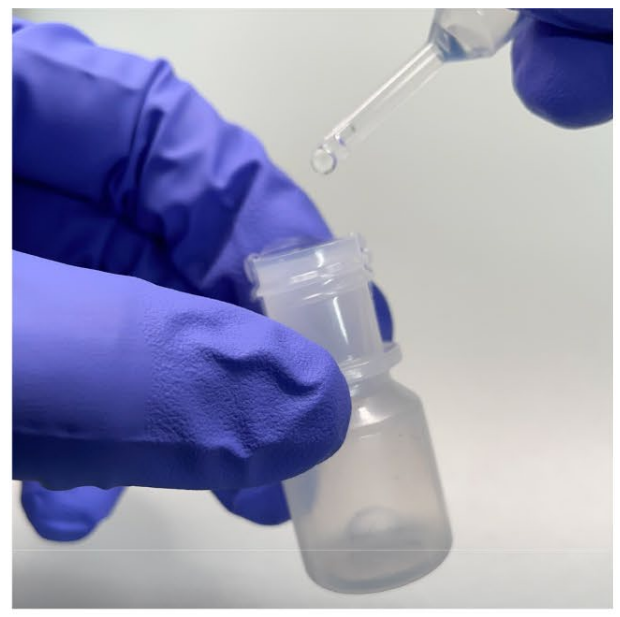

Step 11 - Resuspend freeze-dried Cas12a-RNP pellet in dropper tube with one drop of rehydration buffer.

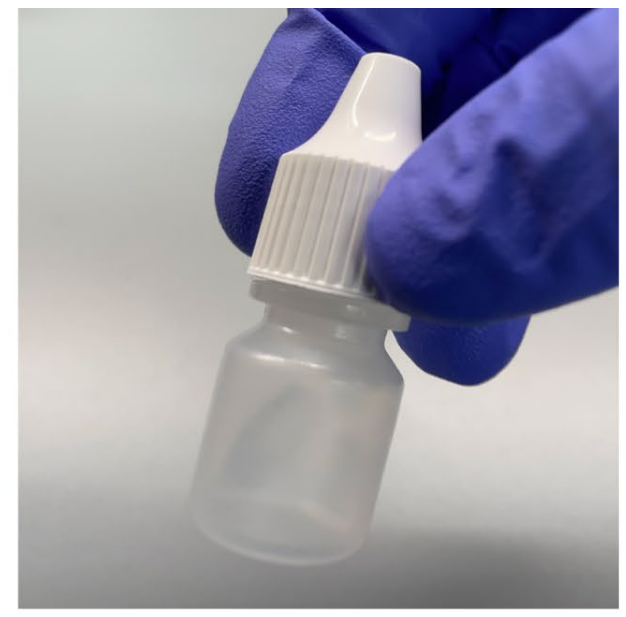

Step 12 - Then add one drop of the RPA reaction into dropper tube. Cap and swirl to mix.

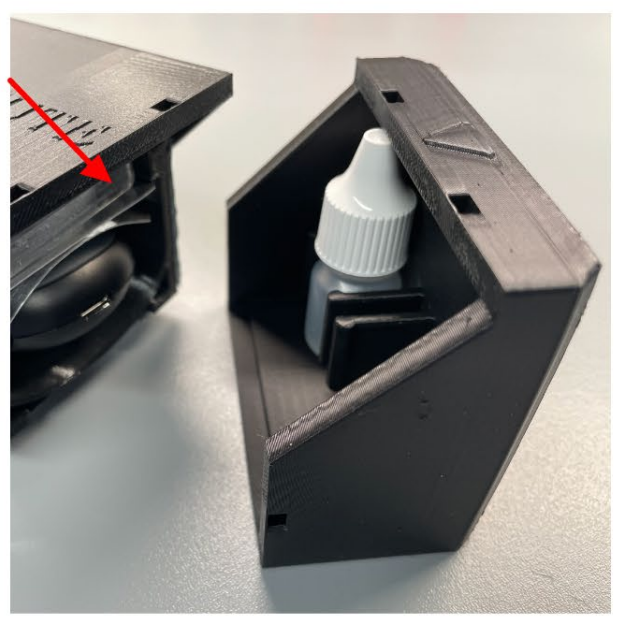

Step 13 - Place the tube in the slot in the incubator lid. Secure lid and incubate for 30 minutes.

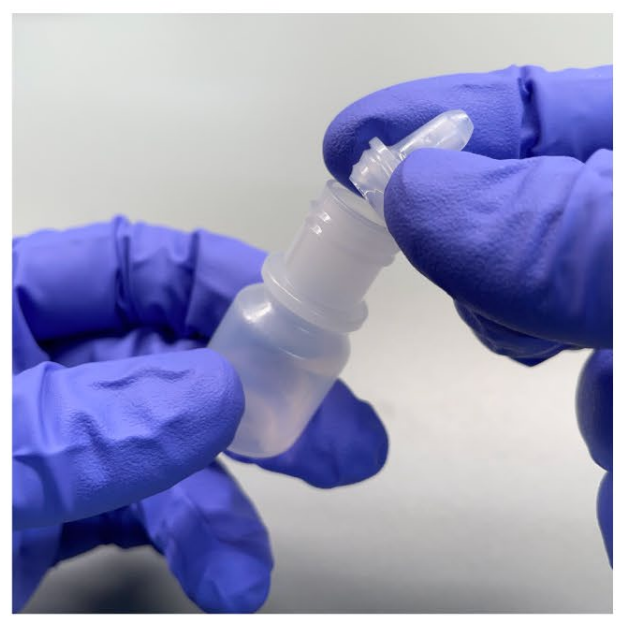

Step 14 - When complete, take out dropper and add all of the LFA dilution buffer to the tube. Snap on dropper tip to tube.

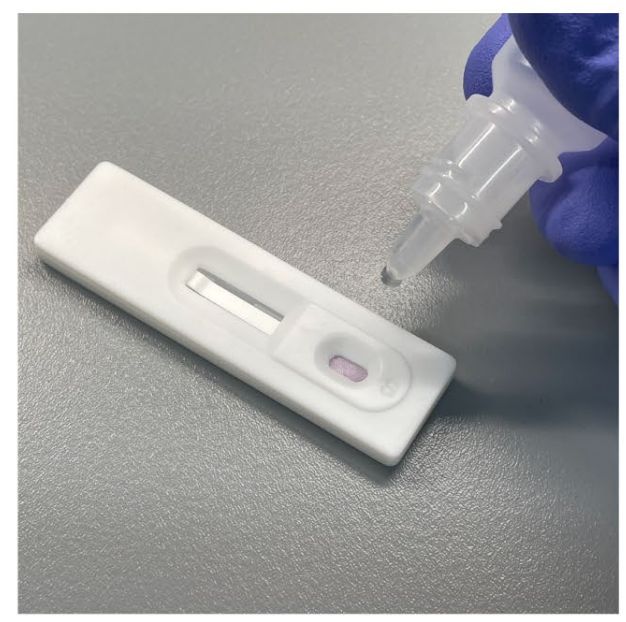

Step 15 - Shake the dropper tube to mix thoroughly, then apply one drop to the LFA strip.

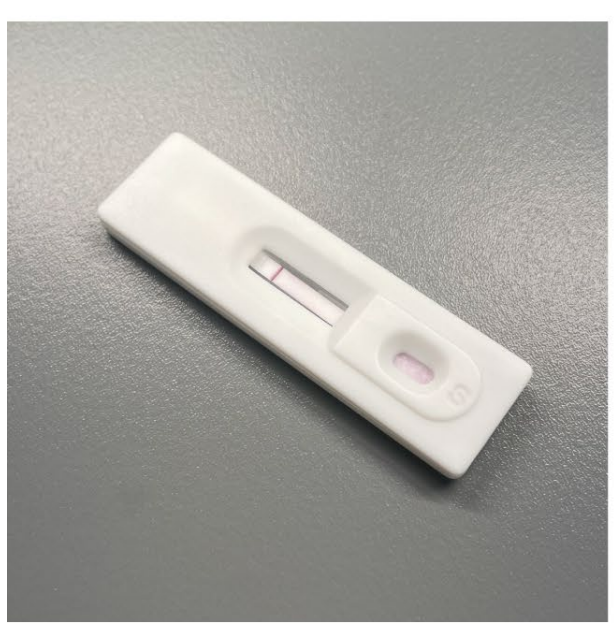

Step 16 - When the LFA strip is fully developed, take picture of indicator bands for analysis.

Supplementary Figure S13 (continued). Full workflow of sample processing using the field-deployable platform. The workflow illustrates stepwise operation: assembling the processor, performing in-filter lysis/RPA in the incubator, collecting amplicons, performing Cas12a reactions with rehydrated reagents in dropper bottles, and developing LFA strips.

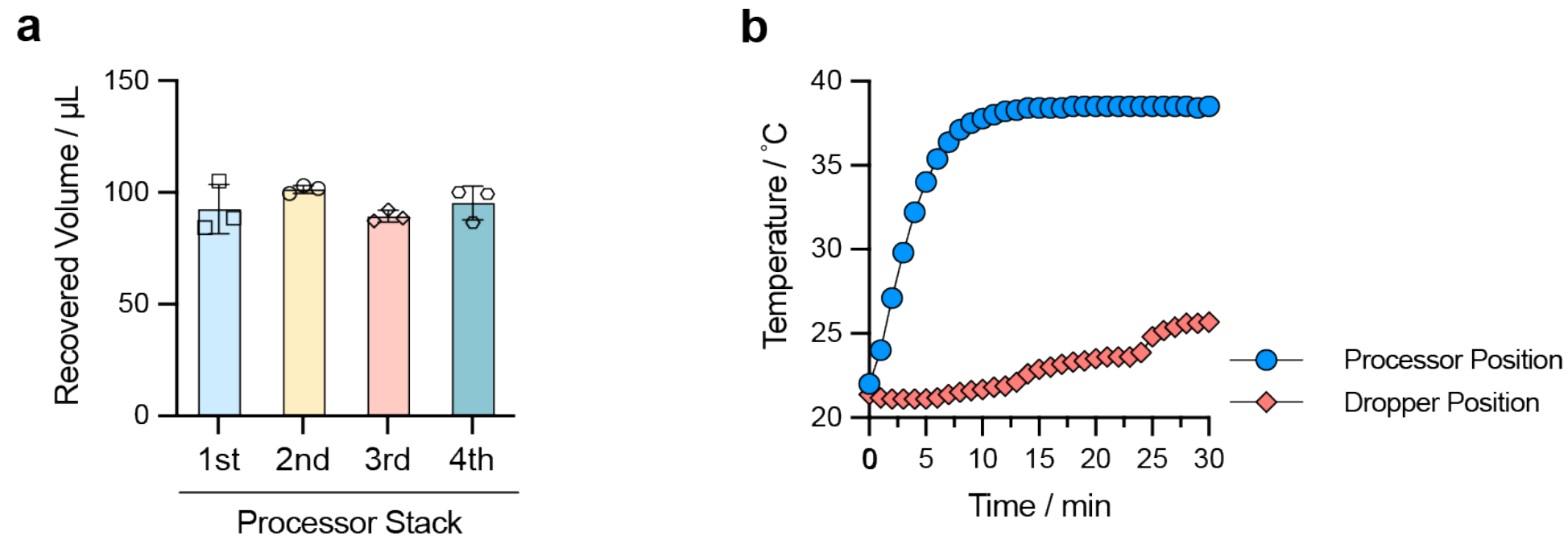

Supplementary Figure S14. Benchmarking of device performance. (a) Consistency of volume recovery from the processor stacks. Four different devices were tested for expelled fluid recovery, using an initial applied volume of 200 µL. New silicone backing pads were used for each measurement. Data points are n = 3 independent measurements. (b) Temperature stability of the portable incubator. Temperatures were measured with a probe at positions corresponding to the processor stack and the dropper bottle. The handheld warmer was set to a temperature of 49ºC/120ºF.

a

gaattcgagctcggtacccggggatccAAGCTGGCGAAGAACTACAACCCTGCTAACACATTGTTTACGTTAGAGTTTGGTTTAAATGATTTCA
TGAACTACAACCGTAGCGTGCCGGAAGTAAAAGCGGATTACGCTGAAGCTCTGATTCGTTTAACAGACGCTGGCGCGAAGAACTTCATGTTGAT
GACACTGCCTGATGCAACCAAAGCACCACAGTTCAAATACTCAACACAGGAAGAAATTGAAACGATTCGTGCGAAAGTATTGAAAATGAACGAG
TTCATCAAAGCACAAGCGATGTACTACAAAGCCCAAGGCTACAACATCGCATTGTTTGATACACACGCACTGTTTGAGAAGTTAACCTCGGCAC
CAGAAGAACACGGTTTCGTAAATGCAAGCGACCCTTGCTTAGACATCAACCGTTCATCCTCTGTTGACTACATGTACACTCACTCATTACGTTC
TGAATGTGCAGCATCTGGCGCAGATAAGTTCGTGTTCTGGGATGTGACTCACCCAACtactagtagcggccgctgcagg

b

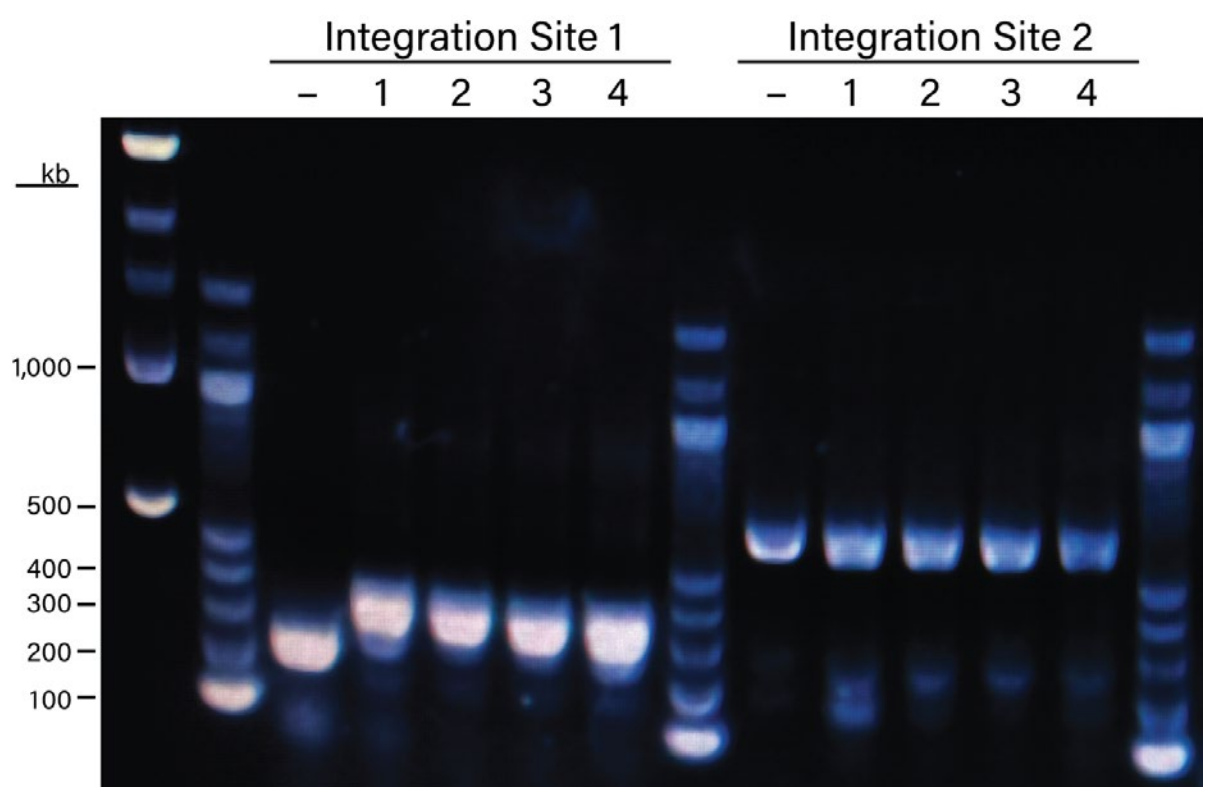

Site 1 (No integration = 241 bp; Single integration = 328, 389 bp);
Site 2 (No integration = 601 bp; Single integration = 429, 648 bp)

Supplementary Figure S15. V. alg gene-integrated E. coli (VIE) strain harboring the tlh gene. (a) *tlh* gene fragment used to construct the clone integration plasmid for genomic insertion. Capitalized highlighted nucleotides indicate the *tlh* gene; lowercase nucleotides show insertion regions. (b) PCR confirmation of genomic integration of *tlh* fragment. Four clones (1–4) were screened at two potential integration sites by PCR. The parental *E. coli* BW25113 strain was used as a negative control (–). Band patterns indicative of successful genomic integration are indicated. Integrant #1 was selected for all experiments in this study.

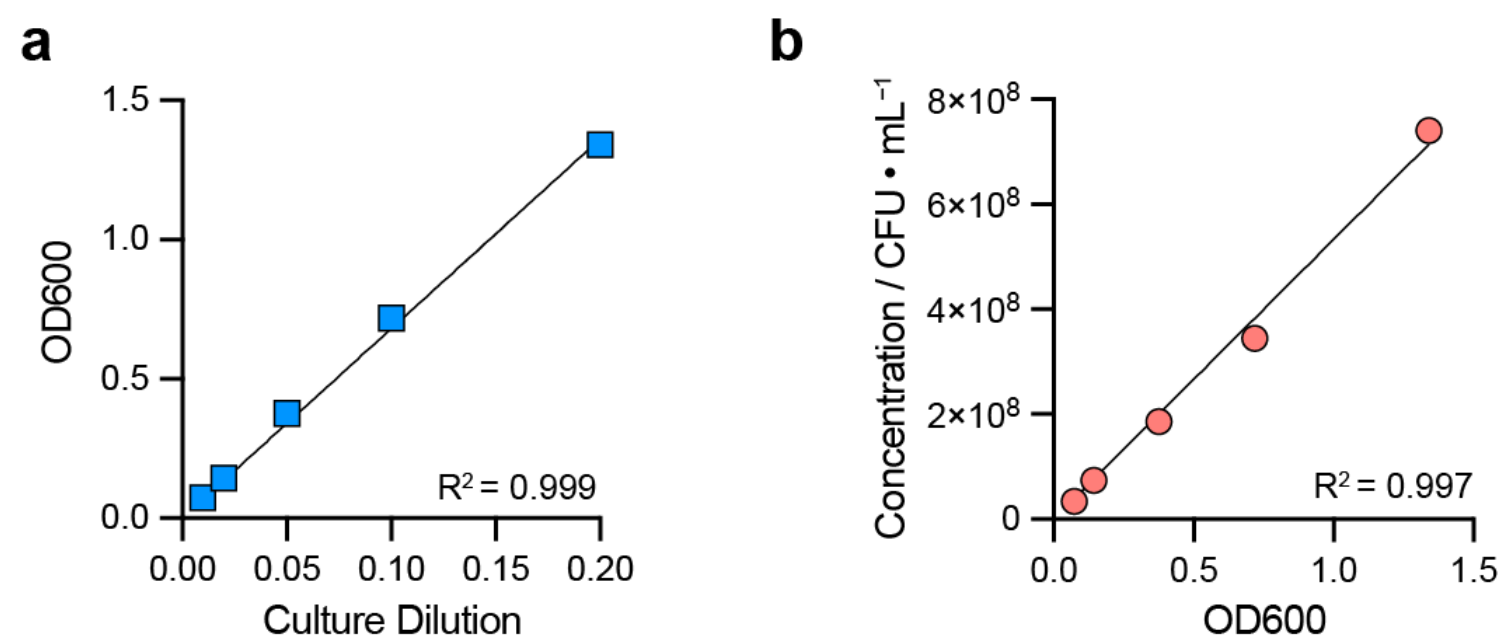

Supplementary Figure S16. VIE standard curves. (a) OD600 vs. Culture dilution factor, (b) Correlation between VIE cell colony-forming units (CFUs) and optical density (OD) at 600 nm. Correlation curves were established and used to quantify CFU concentrations from cultures.

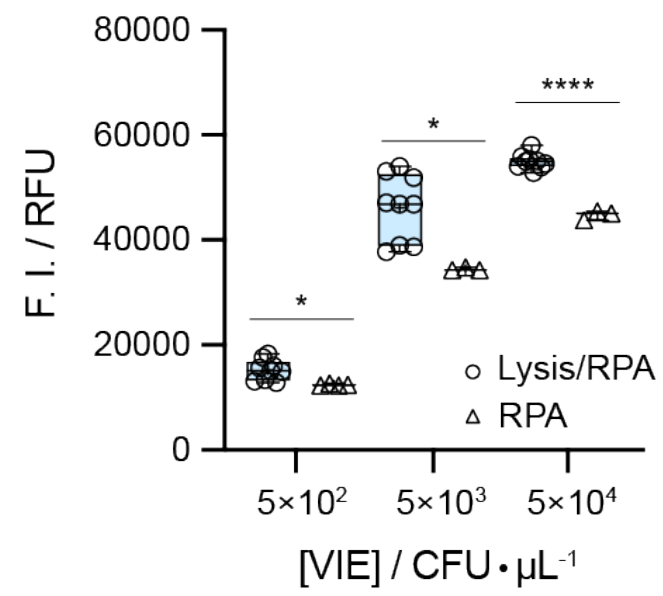

Supplementary Figure S17. Comparison of RPA–CRISPR and Lysis/RPA–CRISPR for VIE detection. Fluorescence intensities were compared across varying concentrations of VIE cells, n = 3–9 independent experiments. Box represents the 25th to 75th percentile and whiskers represent smallest to largest intensities. "+" sign represents mean. ****$p < 0.0001$ and *$p < 0.05$ based on un-paired T-test.

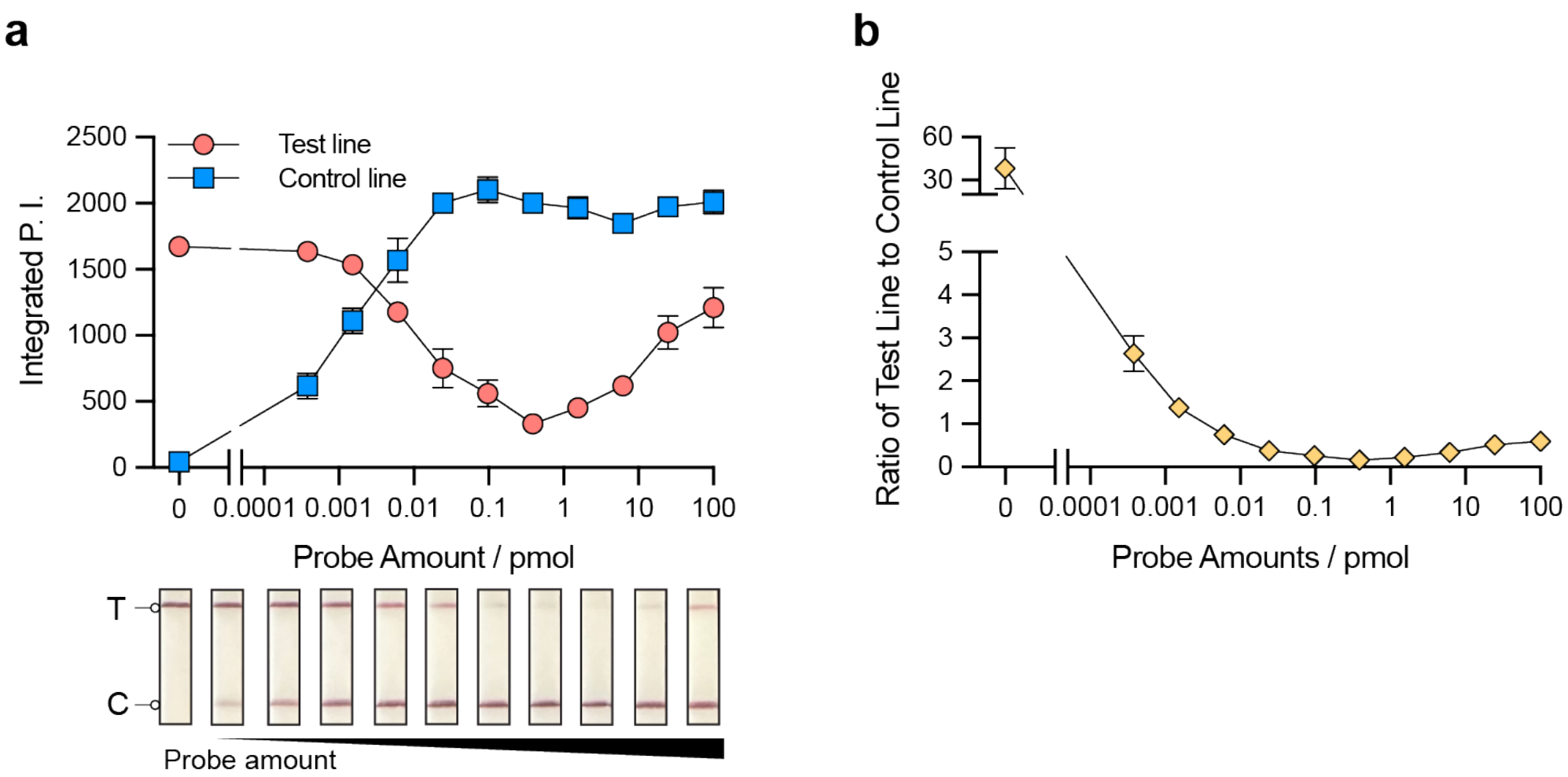

Supplementary Figure S18. Screening of optimal FB probe concentration in a flat format. (a) Test and control line development with varying probe amounts, using a sample volume of 50 µL, *n* = 2 independent experiments. Images were captured using a smartphone and processed as described in Methods. Error bars represent the combined sample standard deviation. (b) Ratio of test-line to control-line mean pixel intensities.

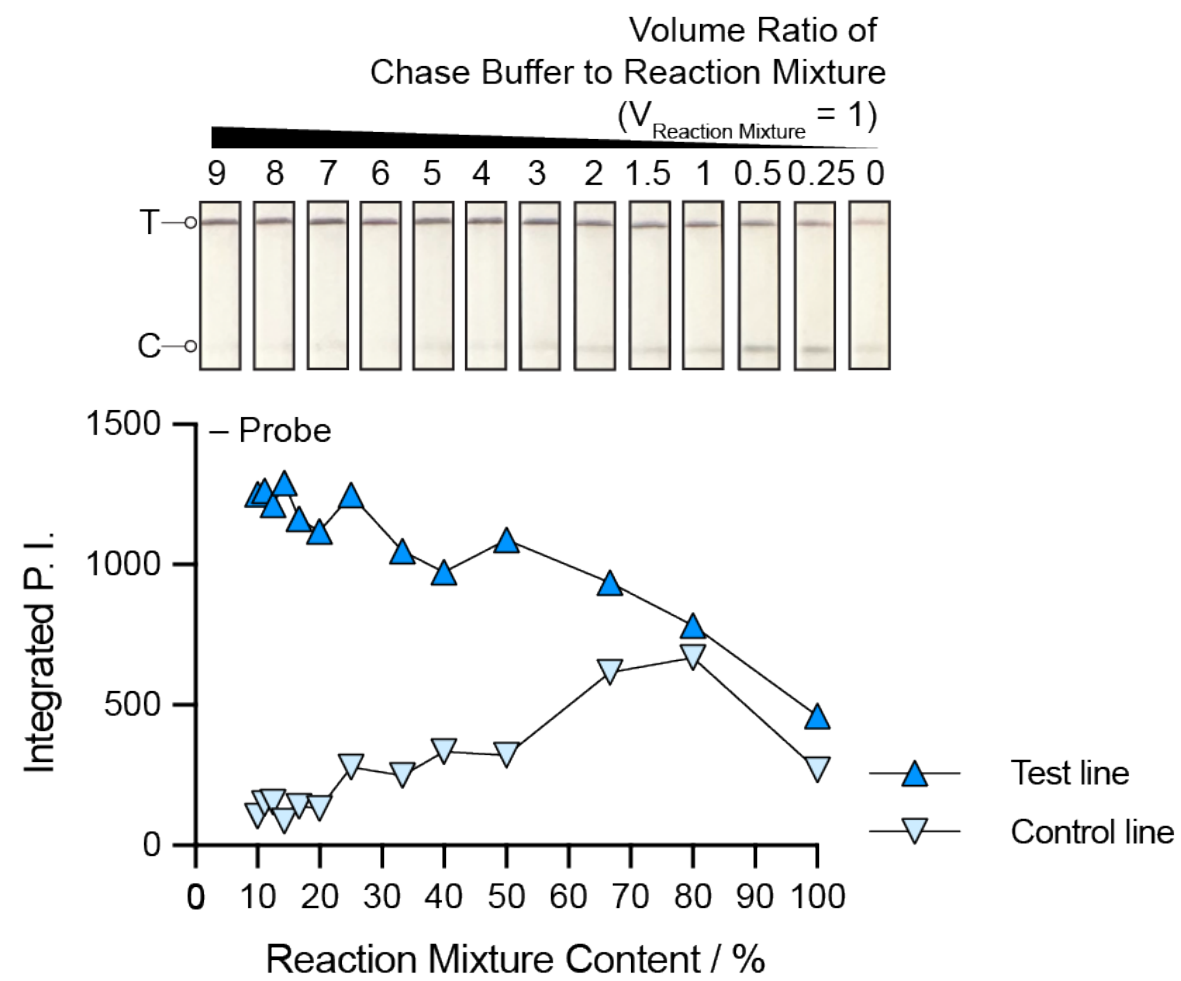

Supplementary Figure S19. Comparison of LFA outputs with varying buffer compositions. The lysis/RPA-CRISPR reaction mixture (Reaction Mixture), without target or FB probe, was diluted at varying volume ratios with Milenia Assay Buffer (Chase Buffer). A volume of 50 µL of diluted reaction mixture was applied to the sample application area of the strip, and LFAs were run in a flat format. Images (top) and pixel intensities (bottom) of test and control lines were captured using a smartphone and processed as described in Methods. Error bars represent the transverse standard deviation of the test- and control-lines. Numbers above images indicate the Chase Buffer to Reaction Mixture volume ratio. High content of reaction mixture (> ~ 30%) resulted in apparent aggregation of gold nanoparticle complexes on the strips.

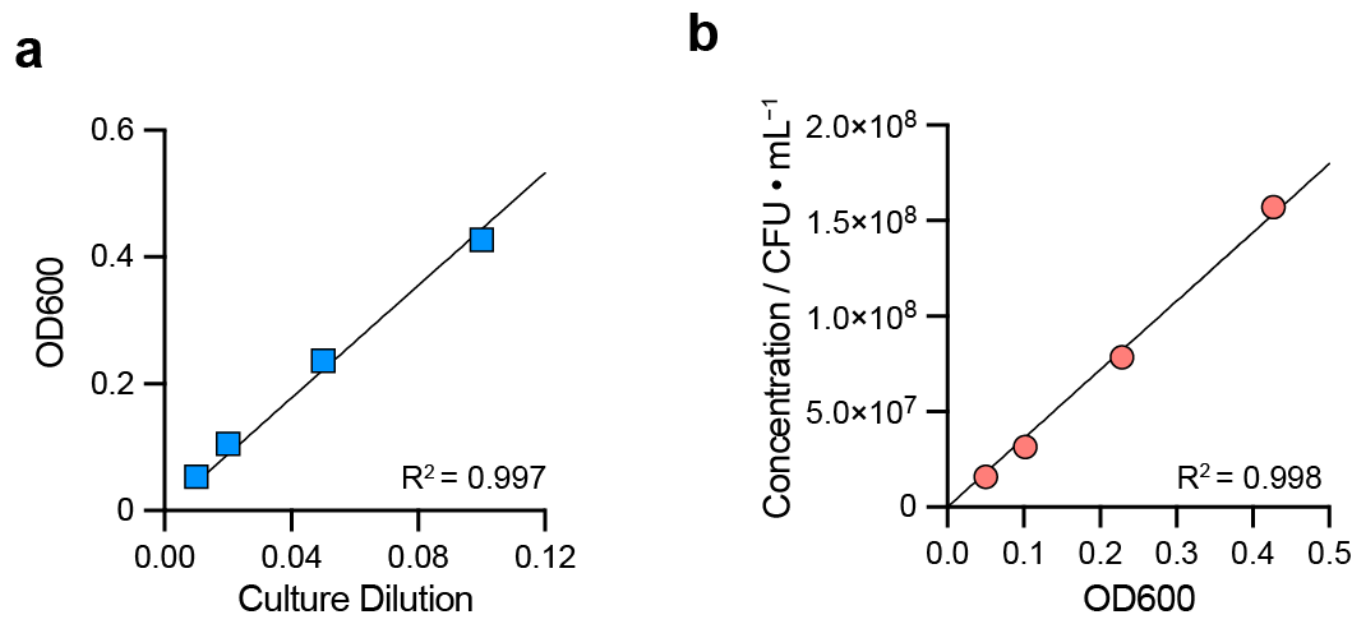

Supplementary Figure S20. Standard curves of authentic V. alg strain XII-53 cell CFUs. (a) OD600 vs. Culture dilution factor, (b) Correlation between *V. alg* XII-53 cell colony-forming units (CFUs) and optical density (OD) at 600 nm. Correlation curves were established and used to quantify CFU concentrations from cultures.

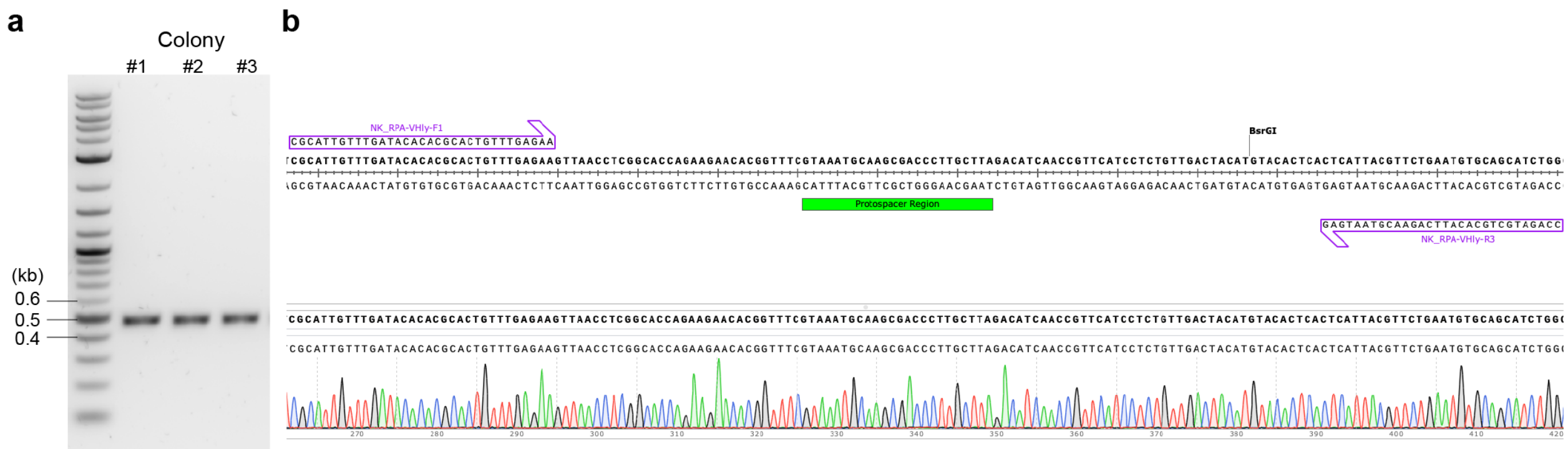

Supplementary Figure S21. Confirmation of the tlh gene in authentic V. alg strain XII-53. (a) Colony PCR of colonies 1–3 targeting the region spanning the forward and reverse RPA primer sites and the protospacer (expected amplicon size: 486 bp). Corresponding bands confirmed successful amplification. (b) Sanger sequencing of PCR amplicons confirmed the presence of the target gene.

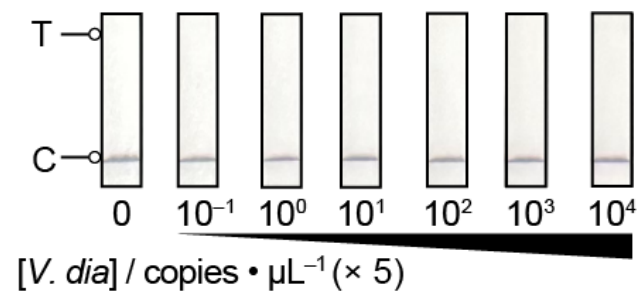

Supplementary Figure S22. Detection of kit-purified gDNA from authentic V. dia strain 122 using the V. alg biosensor (continued from Figure 5j). Images of LFA strips were captured with a smartphone. No significant test-line was observed, demonstrating biosensor specificity.

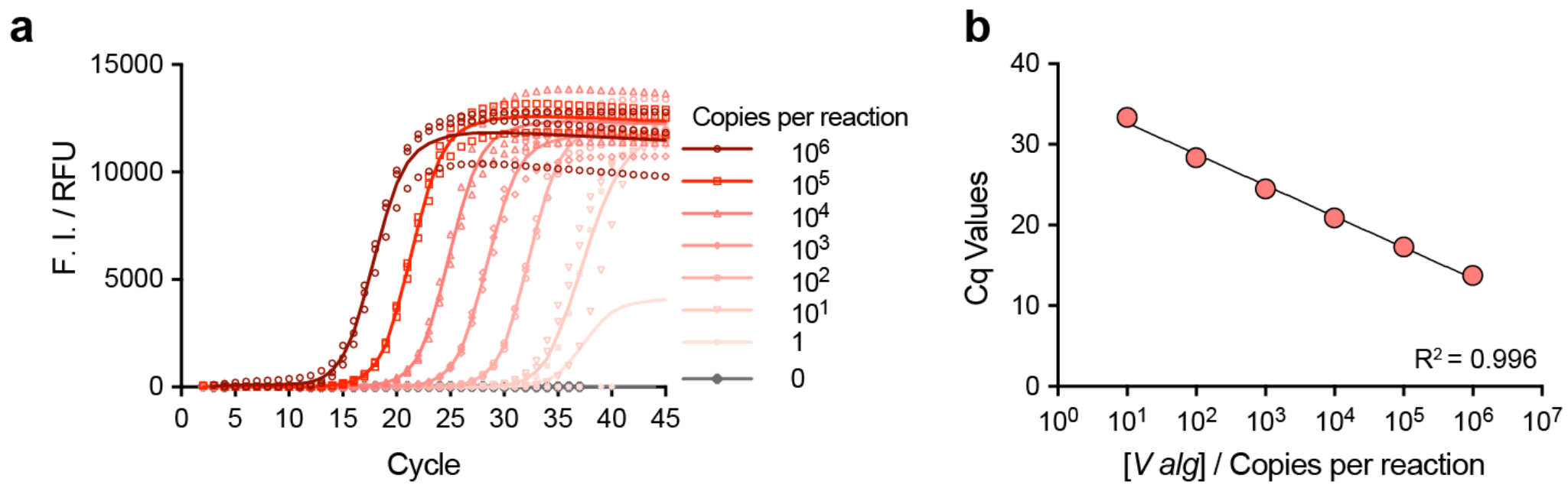

Supplementary Figure S23. Quantitative PCR (qPCR) validation of gDNA from V. alg strain XII-53. qPCR primers were designed to amplify the same region as the RPA amplicons. gDNA copy numbers were inferred from the genome size of *V. alg* strain XII-53 and the measured DNA concentration. (a) Amplification plots of serial dilutions showing real-time fluorescence versus cycle number for gDNA from authentic *V. alg* strain XII-53. (b) Standard curve plotting log-transformed input copy number against Cq, demonstrating assay efficiency. The assay achieved a detection limit of 10 copies per reaction.

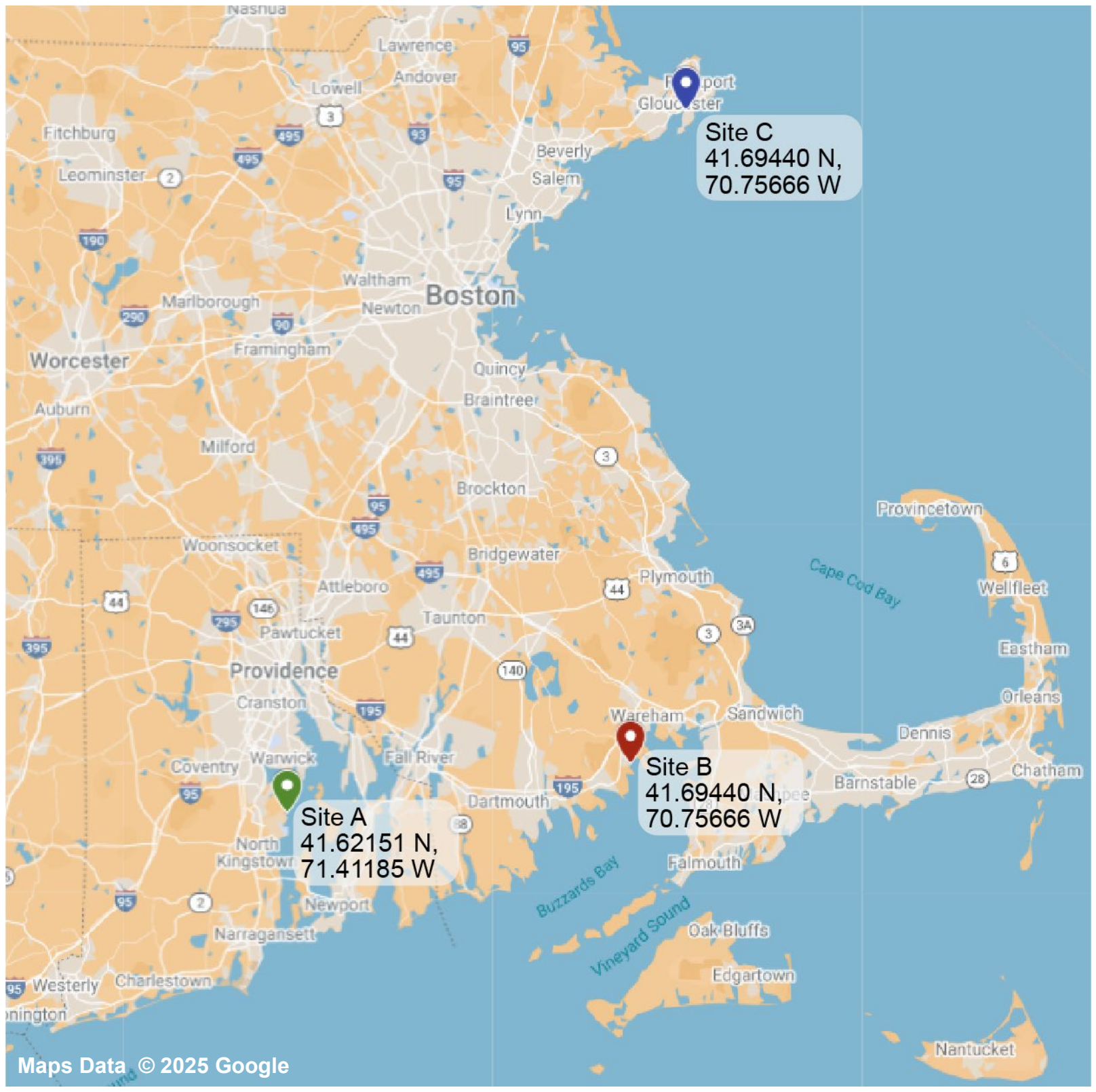

Supplementary Figure S24. Map of natural seawater collection sites. Samples were collected from three geographically distinct public coastal sites to capture environmental variability arising from variable organic and chemical constituents, temperature, currents, and biological activity. Seawater samples were collected and exported in accordance with relevant permits and local laws.

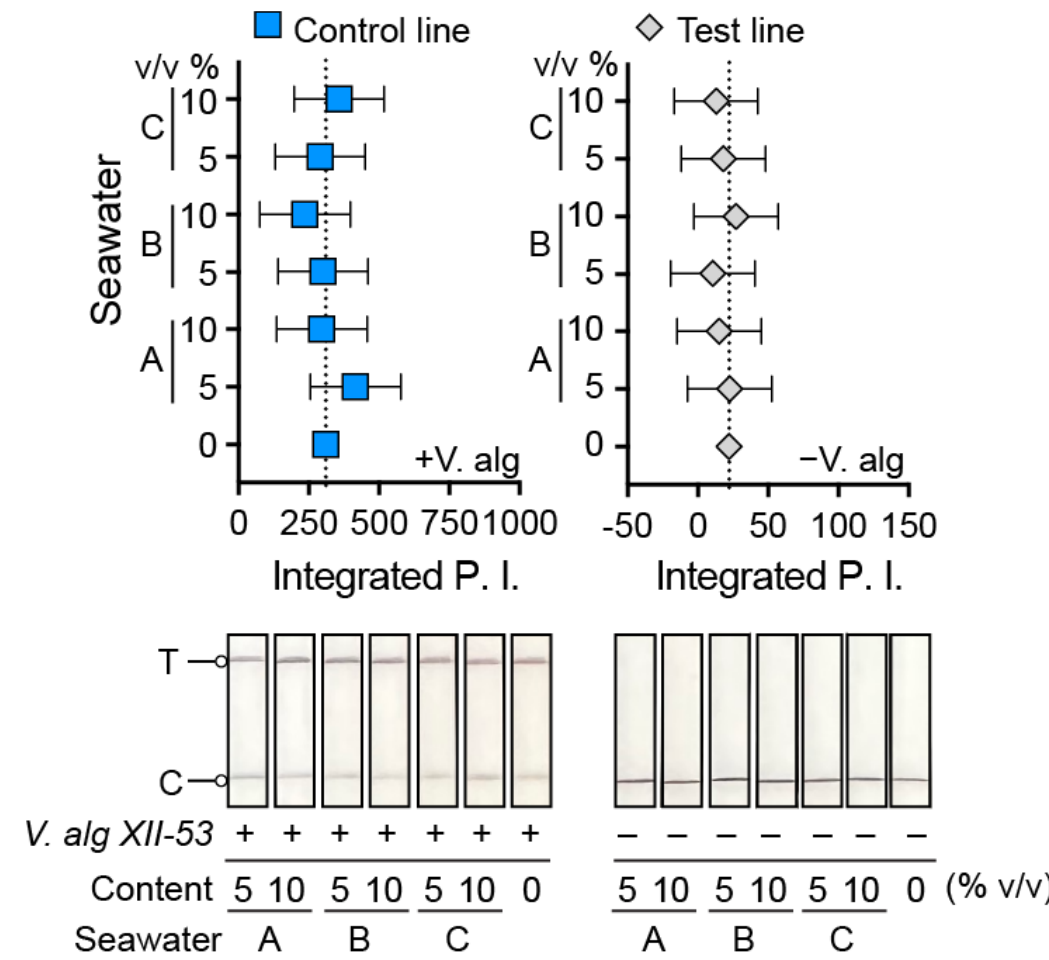

Supplementary Figure S25. Tolerance of LFA strips to natural seawater. Shown are background line intensities, including control lines for positive samples and test lines for negative controls (continued from Fig. 5k). Seawater collected from three geographically distinct sites (Samples A, B, C; for locations see Supplementary Figure S23 and Table S3) was added at 5% or 10% (v/v) to RPA reactions with kit-purified gDNA from *V. alg* strain XII-53 (0 copies/µL negative control; 500 copies/µL positive control, equivalent to 830 aM). Reactions were followed by Cas12a detection with LFA readouts. ANOVA yielded $F(6,14) = 3.10$ and $F(6,14) = 0.680$ for control-line (positive sample) and test-line (negative sample) intensities, corresponding to p-values of 0.0379 and 0.669, respectively. Dunnett's post-hoc test showed no significant differences in both line intensities relative to 0% (v/v) seawater, for both positive and negative target conditions, regardless of sample source or seawater content up to 10% (v/v) (all adjusted $p > 0.5$). Plotted are the mean signals at different concentrations of seawater, with error bars representing 95% confidence intervals, $n = 3$ independent experiments.

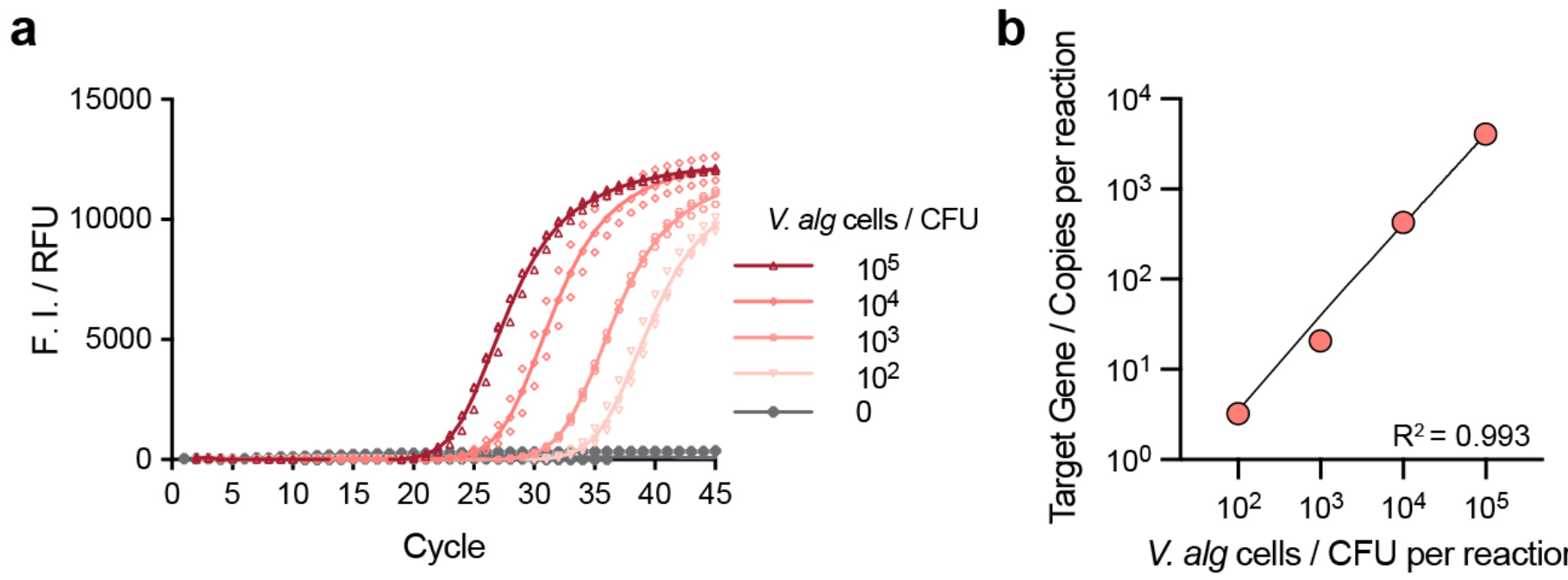

Supplementary Figure S26. qPCR benchmarking of target release from live authentic V. alg strain XII-53 cells. (a) Amplification plots of serial dilutions showing real-time fluorescence versus cycle number for whole *V. alg* strain XII-53 cells (10^2–10^5 CFU), quantified by the established CFU–OD correlation curve (Fig. S20). (b) Using the standard curve generated from gDNA (Fig. S23), qPCR analysis of *V. alg* cultures indicated that ~3.4% of target copies were released relative to CFU counts.

Supplementary Table S1. Summary of nucleic acid sequences used in this study. For target genes, optimal primer binding sites are shown in italics (blue), protospacer regions for optimal crRNAs are in bold, and PAM sequences are underlined. For crRNAs, direct repeats are underlined and protospacer regions are in bold. For crRNAs and RPA primers designed against non-target (control) species, intended mismatches relative to binding sites are indicated in red. In cases where mismatches of crRNAs occur within the PAM sequence, the corresponding crRNAs are marked with asterisks, with the number of asterisks indicating the number of PAM mismatches. crRNAs with PAMs oriented 3′ to 5′ are marked with a superscript R. All sequences are shown in the 5′ to 3′ direction and provided in a ready-to-use orientation.

Sequence Name	Sequence (5′ to 3′)
**Targets**	
Vibrio alginolyticus (*V. alg*)<br>Strain 2010V-1102 (*st1*)<br>(GenBank: CP046813.1)<br><br>Thermolabile hemolysin (*tlh*)	GTGGTTAGCGCGCAAGAAAATCAAACTTATACGTACGTTCGATGCTGGTATCGTA<br>CGAGCCACTCTAAGGACGATGCGGCTACCGATTGGAAATGGGCAAAAAACCAA<br>GATGGTAGTGACTTCACTATCGATGGTTATTGGTGGAGCTCTGTTTCATTTAAAA<br>ACATGTTCTACACCAATACCTCACAAAACGTAATTCGCCAGCGTTGTGAGGAAAC<br>ACTAGATCTAGCGAACGAGAACGCAGACATTACCTTTTTTGCTGCCGACAATCGT<br>TATTCTTATAACCACACGATCTGGAGCAATGATGCTGCAATGCAACCAGATCAAA<br>TCAACAAGGTAGTGGCTTTAGGCGATAGCTTGTCTGATACAGGCAACATCTTTAA<br>CGCGTCCCAATGGCGCTTCCCTAACCCGAATAGCTGGTTCTTGGGTCACTTCTC<br>AAACGGTTTTGTTTGGACGGAATACGTTGCTAAAGCCAAAAACTTACCGCTATAC<br>AACTGGGCAGTTGGTGGCGCAGCAGGTGAAAACCAATACATCGCGCTAACTGG<br>TGTCGGCGAACAAGTTTCTTCTTACTTAACCTACACAAAGCTGGCGAAGAACTAC<br>AACCCTGCTAACACATTGTTTACGTTAGAGTTTGGTTTAAATGATTTCATGAACTA<br>CAACCGTAGCGTGCCGGAAGTAAAAGCGGATTACGCTGAAGCTCTGATTCGTTT<br>AACAGACGCTGGCGCGAAGAACTTCATGTTGATGACACTGCCTGATGCAACCAA<br>AGCACCACAGTTCAAATACTCAACACAGGAAGAAATTGAAACGATTCGTGCGAAA<br>GTATTGAAAATGAACGAGTTCATCAAAGCACAAGCGATGTACTACAAAGCCCAAG<br>GCTACAACAT*CGCATTGTTTGATACACACGCACTGTTTGAGAA*GTTAACCTCGGC<br>ACCAGAAGAACACGGTTTC**GTAAATGCAAGCGACCCTTGCTT**AGACATCAACCG<br>TTCATCCTCTGTTGACTACATGTACACTCA*CTCATTACGTTCTGAATGTGCAGCAT*<br>*CTGG*CGCAGATAAGTTCGTGTTCTGGGATGTGACTCACCCAAC
Vibrio alginolyticus (*V. alg*)<br>Strain VIB283 (*st2*)<br>(GenBank: GQ292538)<br><br>Thermolabile hemolysin (*tlh*)	GTGGTTAGCGCGCAAGAAAATCAAACTTATACGTACGTTCGATGCTGGTATCGTA<br>CGAGCCACTCTAAGGACGATGCGGCTACCGATTGGAAATGGGCAAAAAACCAA<br>GATGGTAGTGACTTCACTATCGATGGTTATTGGTGGAGCTCTGTCTCATTTAAAA<br>ACATGTTCTACACCAATACCTCACAAAACGTAATTCGCCAGCGTTGTGAAGAAAC<br>ACTAGATCTAGCGAACGAGAACGCAGACATTACATTTTTTGCTGCTGACAATCGT<br>TATTCTTATAACCACACGATCTGGAGCAATGATGCTGCAATGCAACCAGATCAAA<br>TCAACAAGGTAGTGGCTTTGGGCGATAGCTTGTCTGATACAGGCAACATCTTTAA<br>CGCGTCCCAATGGCGCTTCCCTAACCCGAATAGCTGGTTCTTGGGTCACTTCTC<br>AAACGGTTTTGTTTGGACGGAATACGTTGCTAAAGCCAAAAACTTACCGCTATAC<br>AACTGGGCAGTTGGTGGCGCAGCAGGTGAAAACCAATACATCGCGCTAACTGG<br>TGTCGGCGAACAAGTTTCTTCTTACTTAACCTACACAAAGCTGGCGAAGAACTAC<br>AACCCTGCTAACACATTGTTTACGTTAGAGTTTGGTTTAAATGATTTCATGAACTA<br>CAACCGTAGCGTGCCGGAAGTAAAAGCGGATTACGCTGAAGCTCTGATTCGTTT<br>AACAGACGCTGGCGCGAAGAACTTCATGTTGATGACACTGCCTGATGCAACCAA<br>AGCACCACAGTTCAAATACTCAACACAGGAAGAAATTGAAACGATTCGTGCGAAA<br>GTATTGAAGATGAACGAGTTCATCAAAGCACAAGCGATGTACTACAAAGCCCAA<br>GGCTACAACAT*CGCATTGTTTGATACACACGCACTGTTTGAGAA*GTTAACCTCGG<br>CACCAGAAGAACACGGTTTC**GTAAATGCAAGCGACCCTTGCTT**AGACATCAACC<br>GTTCATCCTCTGTTGACTACATGTACACTCA*CTCATTACGTTCTGAATGTGCAGC*<br>*ATCTGG*CGCAGATAAGTTCGTGTTCTGGGATGTGACTCACCCAAC
Vibrio alginolyticus (*V. alg*)<br>Strain V05 (*st3*)<br>(GenBank: GQ300939.1)<br><br>Thermolabile hemolysin (*tlh*)	GTGGTTAGCGCGCAAGAAAATCAAACTTATACGTACGTTCGATGCTGGTATCGTA<br>CGAGCCACTCTAAGGACGATGCGGCTACCGATTGGAAATGGGCAAAAAACCAA<br>GATGGTAGTGACTTCACTATCGATGGCTATTGGTGGAGCTCTGTTTCATTTAAAA<br>ACATGTTCTACACCAATACCTCACAAAACGTAATTCGTCAGCGTTGTGAAGAAAC<br>ACTAGATCTAGCGAACGAGAACGCAGACATTACATTTTTTGCTGCTGACAATCGT<br>TATTCTTATAACCACACGATCTGGAGCAACGATGCTGCAATGCAGCCAGATCAG<br>ATAAACAAAGTAGTGGCTTTAGGCGATAGCTTGTCTGATACAGGCAACATCTTTA<br>ACGCGTCCCAATGGCGCTTTCCTAACCCGAATAGCTGGTTCTTGGGTCACTTCT<br>CAAACGGTTTTGTTTGGACGGAATACGTTGCTAAAGCCAAAAACTTACCGCTATA<br>CAACTGGGCAGTTGGTGGCGCAGCAGGTGAAAACCAATACATCGCGCTAACTG<br>GTGTCGGCGAACAAGTTTCTTCTTACTTAACCTACACAAAGCTGGCGAAGAACTA<br>CAACCCTGCTAACACATTGTTTACGTTAGAGTTTGGTTTAAATGATTTCATGAACT<br>ACAACCGTAGCGTGCCGGAAGTAAAAGCGGATTACGCTGAAGCTCTGATTCGTT<br>TAACAGACGCTGGCGCTAAGAACTTCATGTTGATGACACTGCCTGATGCAACCA<br>AAGCACCACAGTTCAAATACTCAACACAGGAAGAAATTGAAACGATTCGTGCGAA<br>AGTATTGAAAATGAACGAGTTCATCAAAGCACAAGCGATGTACTACAAAGCCCAA

	GGCTACAACAT*CGCATTGTTTGATACACACGCACTGTTTGAGAA*GTTAACCTCGG<br>CACCAGAAGAACACGGTTTC**GTAAATGCAAGCGACCCTTGCTT**AGACATCAACC<br>GTTCATCCTCTGTTGACTACATGTACACTCA*CTCATTACGTTCTGAATGTGCAGC*<br>*ATCTGG*CGCAGATAAGTTCGTGTTCTGGGATGTGACTCACCCAAC
Vibrio diabolicus (*V. dia*)<br>Colony 272 (*st1*)<br>(GenBank: CP078739)<br><br>Thermolabile hemolysin (*tlh*)	GTGGTCAGCACGCAAGAAAATCAAACCTATACTTACGTTCGCTGCTGGTATCGTA<br>CGAGCCACTCTAAGGATGATGCGGCTACCGACTGGAAATGGGCAAAAAACCAA<br>GATGGTAGTGACTTCACTATCGACGGTTATTGGTGGAGCTCTGTTTCATTTAAAA<br>ACATGTTCTACACCAACACTTCACAAAACGTGATTCGCCAGCGCTGTGAAGAAAC<br>ACTAGATCTGGCGAACGAGAACGCAGACATTACGTTTTTTGCTGCTGACAATCG<br>CTATTCTTATAACCACACGATCTGGAGCAATGATGCTGCAATGCAACCAGATCAA<br>ATCAACAAAGTAGTGGCTTTGGGCGACAGCTTGTCTGATACAGGCAACATCTTTA<br>ACGCTTCCCAATGGCGCTTCCCTAACCCAAACAGCTGGTTCTTAGGTCACTTCTC<br>GAACGGTTTTGTTTGGACGGAATACGTTGCTAAAGCCAAAAACTTACCGCTTTAT<br>AACTGGGCAGTTGGCGGCGCAGCAGGTGAGAACCAATACATTGCGCTAACAGG<br>TGTCGGCGAACAAGTTTCTTCTTACTTGACCTACACAAAACTGGCGAAAAACTAC<br>AACCCAGCAAACACATTGTTTACGTTGGAGTTTGGTTTAAATGATTTCATGAACTA<br>CAACCGTAGCGTGCCGGAAGTAAAAGCGGATTACGCTGAAGCTCTGATTCGTCT<br>AACAGACGCGGGCGCGAAGAACTTCATATTGATGACACTGCCAGACGCAACCAA<br>AGCACCACAGTTCAAATACTCAACGCAGGAAGAAATTGAAACGATTCGCGCGAA<br>AGTATTGAAAATGAACGAGTTCATCAAAGCACAAGCGATGTACTATAAAGCTCAA<br>GGTTACAACATTTCATTGTTTGATACGCACGCGCTGTTTGAGACGTTAACCTCGG<br>CACCAGAAGAACACGGGTTCGTGAATGCAAGTGATCCTTGTCTGGACATCAACC<br>GCTCATCCTCTGTTGACTACATGTACACTCACTCATTACGTTCTGAATGTGCAGC<br>ATCTGGCGCAGATAAGTTTGTGTTCTGGGATGTGACTCACCCAAC
Vibrio diabolicus (*V. dia*)<br>Strain LMG 3418 (*st2*)<br>(GenBank: CP014133)<br><br>Thermolabile hemolysin (tlh)	GTGGTCAGCACGCAAGAAAATCAAACCTATACTTACGTTCGCTGCTGGTATCGTA<br>CGAGCCACTCTAAGGATGATGCGGCTACCGACTGGAAATGGGCAAAAAACCAA<br>GATGGTAGTGACTTCACTATCGACGGTTATTGGTGGAGCTCTGTTTCATTTAAAA<br>ACATGTTCTACACCAACACTTCACAAAACGTGATTCGCCAGCGCTGTGAAGAAAC<br>ACTAGATCTGGCGAACGAGAACGCAGACATTACGTTTTTTGCTGCTGACAATCG<br>CTATTCTTATAACCACACTATCTGGAGCAATGATGCTGCAATGCAACCAGATCAA<br>ATCAACAAAGTAGTGGCTTTGGGCGACAGCTTATCTGATACAGGCAACATCTTTA<br>ACGCTTCCCAATGGCGCTTCCCTAACCCAAACAGCTGGTTCTTAGGTCACTTCTC<br>GAACGGTTTTGTTTGGACGGAATACGTTGCTAAAGCCAAAAACTTACCGCTTTAT<br>AACTGGGCAGTTGGCGGCGCAGCAGGTGAGAACCAATACATTGCGCTAACAGG<br>TGTCGGCGAACAAGTTTCTTCTTACTTGACCTACACAAAACTGGCGAAAAACTAC<br>AACCCAGCAAACACATTGTTTACGTTGGAGTTTGGTTTAAATGATTTCATGAACTA<br>CAACCGTAGCGTGCCGGAAGTAAAAGCGGATTACGCTGAAGCTCTGATTCGTCT<br>AACAGACGCGGGCGCGAAGAACTTCATGTTGATGACACTGCCAGACGCAACCA<br>AAGCACCACAGTTCAAATACTCAACGCAGGAAGAAATTGAAACGATTCGCGCGA<br>AAGTATTGAAAATGAACGAGTTCATCAAAGCACAAGCGATGTACTATAAAGCTCA<br>AGGTTACAACATTTCATTGTTTGATACGCACGCGCTGTTTGAGACGTTAACCTCT<br>GCACCAGAAGAACACGGGTTCGTGAATGCAAGTGATCCTTGTCTGAACATCAAC<br>CGCTCATCTTCTGTGGATTACATGTACACCCATTCATTACGCTCTGAGTGTGCGG<br>CATCTGGCGCAGATAAGTTTGTGTTCTGGGATGTGACTCACCCAAC
Pseudo-nitzschia australis (*P. aus*)<br>Clone L3.15<br>(GenBank: MH376353.1)<br><br>18S ribosomal RNA gene, internal transcribed spacer (ITS) 1, 5.8S ribosomal RNA gene, and ITS 2	GTAGGTGAACCTGCGGAAGGATCATTACCACACCGATCCAAGATCAGTCTT*CAT*<br>*TGTGAATCTGATTTCAGAGGCGGCTTGC*TTTTAAAGCTCTCCGTCTCTGTCGTCA<br>AACTTTTTTA**GTGATGCGCTAGCTACTAGCGTAT**TACAACCCCCCATTCTTAA*CG*<br>*ATTGGTAACTGGAAAGAACCAAATGACC*TAAAGCTAAAATGCAGTGGTCTGGTGT<br>TGCGCCTCGTGCCAACACCGCCCTGTACATTATACCAATTCATTACAACTTTCAG<br>CGGTGGATGTCTAGGTTCCCACAACGATGAAGAACGCAGCGAAATGCGATACGT<br>AATGCGAATTGCAAGACCTCGTGAATCATTAAGATTTTGAACGCACATTGCGCTT<br>TCGGGATTTTCCCGGTAGCATGCTTGTCTGAGTGTCTGTGGATCCCACTCAGCG<br>CTGGTTTAGGCCAGTCGCTGGTTGTTTTGGCCTTGACAGCTACTAGTAGCTGTC<br>TCTGCTTAAGTTCTACGGTATCGTACGTGCATAGATTTAGAGACCTCAACCTGTC<br>TTGTCAAGACGGCGTTGACATTCTTGTCTATCTCTGGTAGCTTGTATTCATTACAA<br>ATATCTGGAGTTTGTTAAGATTGTCATGTTCAGCTGTTTGAAACTTTTCGATTGAG<br>CGTCACTAGATCTAGCTAGCTATACTTTCTAGTACTAGCTAGCTATCCA
Pseudo-nitzschia seriata (*P. ser*)<br>Strain A3<br>(GenBank: JF974030.1)<br><br>18S ribosomal RNA gene, ITS 1, 5.8S ribosomal RNA gene, and ITS 2	GAACCTGCGGAAGGATCATTACCACACCGATCCAAGATCAGTCTTCATTGTGAAT<br>CTGATTTCA*GAGGCGGCTTGCTTTTAAAGCTCTCCGTCG*CTGTCGTCAAACTATT<br>TTC**AGTGATATGCTATTTACTAGTGTA**TCACAACCCCCCATTCTTAACGATTGGT<br>AACTGGAAAGAACCAAATGACCTAAA*GCTAAAATGCAGTGGTCTGGTGTTGCGC*<br>*CTC*GTGCCAACACCGCCCTGTACATTATATCAATTCATTACAACTTTCAGCGGTG<br>GATGTCTAGGTTCCCACAACGATGAAGAACGCAGCGAAATGCGATACGTAATGC<br>GAATTGCAAGACCTCGTGAATCATTAAGATTTTGAACGCACATTGCGCTTTCGGG<br>ATTTTCCCGGTAGCATGCTTGTCTGAGTGTCTGTGGATCCCACTCAGCGCTGGT<br>TTAGGCCGGTCGCTGGTTGTTTTGGCCTTGACAGCTACTAGTAGCTGTCTCTGC<br>TTAAGTTCTACGGTATCGTACGTGCATAGATTTAGTTACCTCAACCTGTCTTGTCA<br>AGACGGCGTTGACACTCTTGTCTATCTCTGGTAGCTTGTATTTATTACAAATATCT<br>GGAGTTTGTTAAGATTGTCATGTTCAGCTGTTTGAAACTTTTCGATTGAGTGTCA<br>CTAGATCTAGCTAGCTATACTTTCCTGTATTAGCTAGCTATCCAAT

Pseudo-nitzschia americana (*P. ame*)<br>Strain MC3108<br>(GenBank: MT023636.1)<br><br>ITS 1, 5.8S ribosomal RNA gene, and ITS 2	AGATCAGTCTTCATTGTGAATATGATTTCAGATTCTGGTGCCAGCTAGCCTGGTG<br>CTGCTTCTGTCGTCAACCTTTTTCTATGGAGCCGGCGCATTTGCTGTTGCTATTT<br>ATACTGGCAAGGGCGTGTGCGCTAGTTCCAGTGGTGCTTTGTCACCACAACCCC<br>CATTCTTTACGATTGGTAACTGGAAAGAACCAAATGACCTAAAGCAAAAATGCAG<br>CGGTCCGGTGTCGTGCCTCGTGCCGACGCCGCCCTGTACATTATACCTAATAAC<br>ATTACAACTTTCAGCGGTGGATGTCTAGGTTCCCACAACGATGAAGAACGCAGC<br>GAAATGCGATACGTAATGCGAATTGCAAGACCTCGTGAATCATTAAGATTTTGAA<br>CGCACATTGCGCTTTCGGGATTTTCCCGGTAGCATGCTTGTCTGAGTGTCTGTG<br>GATCCCACTCAGCGCTGGTATTGTATTTTACTATACTAATACTAGCTGCTGGTTG<br>TTTTGGCTTTGACTGCGTTTCGCTGTCTTTGCTCAAGTTCTACGGTATCGTACGT<br>GCATAGATGCAGAAAGCTCTACCTGTCTTGCGAAAGACGGCGTTGGCACTCTTG<br>TCTATCTCTGGTAGTAGCAGTAGCTTCTTCCTTGAGTTTATTTGAGTTGTGCCAT<br>GTTCAGCCGTTTGGAAGCTTTTCGGTCGAATTGTCTGTGTCTAGCCTGTGGGGT<br>GCACTTTATTAGTGTGCCACCACTGGCTATCCAATTCCGGATCTCAGATCAA
Pseudo-nitzschia pungens (*P. pun*)<br>Voucher PnPd38<br>(GeneBank: KF040049.1)<br><br>ITS 1, 5.8S ribosomal RNA gene, and ITS 2	TTACCACACCGATCCAAGATCAGTCTTCATTGTGAATCTGATTGCACCGGTCTAG<br>TATTTTTACTAAACCGCTGCCGTCAAACTTAAACTTGCAACGCGATGATTAATTCC<br>GCCTTGCTGCCATTCTTTACGATTGGTAACTGGAAAGAACCAAATGACCTAAAGC<br>AAAAATGATGCAGTGTTTCGGAGCGCTGAGCGGGCGTCTTAAGTATTAGATGCA<br>TACCAAACCGACTCTATTGCTGGCACTGCACATTACACCTAATACATTACAACTTT<br>CAGCGGTGGATGTCTAGGTTCCCACAACGATGAAGAACGCAGCGAAATGCGATA<br>CGTAATGCGAATTGCAAGACCTCGTGAATCATCAAGATTTTGAACGCACATTGCG<br>CTTTCGGGTATTCCCGGTAGCATGCTTGTCTGAGTGTCTGTGGATCCCACTCAG<br>CGCTGGCTTCGTGCTGGCTGCTGGTATTTTGGCCGTGACTGAATTTGTTTAGTCT<br>CGGCTTAAGTTTTACGTTAAGTACGTGCATAGATCTAGAAAGCTCTGCCCGTTTA<br>GTTAAAAACACGGCGTTGACACTCTTGTCTATCTCTGGTAGGTTTATTCAACTGG<br>AGTTTGCTACGAGTTGTCTATAGCTGTTTTGAAACTACTGGAATGAGCGCTTGTA<br>GATCTAACTAGCTGGAAACAGTTAGTTATACAATTCCGGATCTCAGATCAAGCAA<br>GAGG
Porites astreoides<br>Heat stress protein 16 (HSP16)[15-17]	CGAUCCCGCGAAAUUAAUACGACUCACUAUAGGGGAGCUUAAACCGUUUAUCU<br>GUGCCAGUGAGAGAUUUAUAGGAGAAGCUAUGCUGGGAGAGCUAUACCAGUU<br>UGCUGAGGAGUACUUUGAAGUUAAGUCAUUGCAAGUGGCUCCAGGACGUUUA<br>GCGAUAGCAGUGUCUACUGUGAACGAUCACAAGUUCGUGGUUAGACUGAAUC<br>UGCGUGGAUUCAAACCAGAAGAAAUCAAGGUCCAACUUCGUGGACAUGAGCU<br>C*ACGGUCACAGGAAAACACAGAGCGGAAGAG*GACGAUUCAUAUUUG**UCAUAU**<br>**CAUUACAGCCGUCGCGUU**UUGUUGCCUGACGACGCAGAA*CUCAGUUCAGUGA*<br>*AGUCACGCUUGUCCAAAG*AAGGCUUGCUAACAAUAGAAGUGGCACGUGACCC<br>AGCUCUGUUACCAAGUGAGAGAAACGUGGAACUUUCCAUGGAAGUUGAUCAU<br>CAGCCACAUCCUGAAGAAGAAGUGCAACAACAACAAGGAGCAAGCGGUAACAG<br>AAGAAGAUUAGAAUAA
Porites astreoides<br>Eukaryotic initiation factor 3, subunit H (EIF3H)[15-17]	CGAUCCCGCGAAAUUAAUACGACUCACUAUAGGGCGUUCUUCCCAUAAGGAAA<br>AUUCAAGUUCCUCCAGUUCUACAAGUCGAAUUGAUAGCGUCCAAGUUGAUGG<br>ACUGGUUGUGCUGAAAAUUAUUAAACAUUGCCAAGAUGAAGGAUCAGCAGGC<br>GAACUUGUUCAAGGAGUCUUACUUGGAUUGAUUCAAAACCAUAAACUUGAGAU<br>AACAAACUGCUUUGCUUUCCCAUCAGUGCGAGUAGGAGAAGAUGAUGACGAA<br>GACGAUGUAAACUACCAGAUGGAGAUGAUGCGUCGUUUACGAGCUGUCAAUG<br>UAGACCAUCUACAUGUGGGCUGGUAUCAAUCAACUUACCUUGGUUCAUUUGU<br>AAACAAAACAUUACUGGACUCACAAUUCAGUUAUCAGAGUUCUAUUGAGGAAU<br>CAGUGGUACUUAUUUAUGAUCCAUGGAAGACCUCACAAGGAAUGCUGUCUUU<br>GAAAGUGUUCCGUUUGUCUGAUAAAAUGAUGGCAAUGUAUAAAGAGGCAGAA<br>UUUAAUGCAGAAAAGCUUGCCAAA*AGUGGUCUCAGUUUUGAGGCAAUGUUUG*<br>*AAGAG*AUUCCACUUGUUAUAAGGAACUCU**UCACUUGUCAAUACUCUGCUGUG**<br>**U**GAAAUUGAAGU*CUUGUCACCUCAUACCCAAACAGAUGAGUUUCU*CAGUCUCU<br>CCACAGGGUCUUAUUUAGAAAAGAAUGUACGACUGCUCAUGGAGGGUGUUGA<br>UG
**crRNAs**	
V alg crRNA1 (V1)*	UAAUUUCUACUAAGUGUAGAU**ACAGACGCUGGCGCGAAGAACUUC**
V alg crRNA2 (V2)*	UAAUUUCUACUAAGUGUAGAU**GUAAAUGCAAGCGACCCUUGCUUA**
V alg crRNA3 (V3)^{R*}	UAAUUUCUACUAAGUGUAGAU**AGAAGUGACCCAAGAACCAGCUAU**
V. alg crRNA4 (V4)*	UAAUUUCUACUAAGUGUAGAU**UAGUACAUCGCUUGUGCUUUGAUG**
V. alg crRNA5 (V5)^{R**}	UAAUUUCUACUAAGUGUAGAU**CGAAACCGUGUUCUUCUGGUGCCG**
P. aus crRNA1 (A1)	UAAUUUCUACUAAGUGUAGAU**GUGAUGCGCUAGCUACUAGCGUAU**
P. aus crRNA2 (A2)	UAAUUUCUACUAAGUGUAGAU**GAGACCUCAACCUGUCUUGUCAAG**
P. aus crRNA3 (A3)^{R}	UAAUUUCUACUAAGUGUAGAU**UAAUGAAUACAAGCUACCAGAGAU**
P. aus crRNA4 (A4)	UAAUUUCUACUAAGUGUAGAU**UAGUACUAGCUAGCUAUCCA**
P. ser crRNA1 (S1)*	UAAUUUCUACUAAGUGUAGAU**AGUGAUAUGCUAUUUACUAGUGUA**
P. ser crRNA1 (S2)	UAAUUUCUACUAAGUGUAGAU**GUUACCUCAACCUGUCUUGUCAAG**
P. ser crRNA1 (S3)**	UAAUUUCUACUAAGUGUAGAU**CUAGUGUAUCACAACCCCCCAUUC**
P. ser crRNA1 (S4)	UAAUUUCUACUAAGUGUAGAU**CUGUAUUAGCUAGCUAUCCAAU**
HSP16 crRNA 1 (H1)	UAAUUUCUACUAAGUGUAGAU**GCGAUAGCAGUGUCUACUGUGAAC**

HSP16 crRNA 2 (H2)[R]	UAAUUUCUACUAAGUGUAGAU**CUGUGACCGUGAGCUCAUGUCCAC**
HSP16 crRNA 3 (H3)	UAAUUUCUACUAAGUGUAGAU**UCAUAUCAUUACAGCCGUCGCGUU**
HSP16 crRNA 4 (H4)[R]	UAAUUUCUACUAAGUGUAGAU**GACAAGCGUGACUUCACUGAACUG**
HSP16 crRNA 5 (H5)[R]	UAAUUUCUACUAAGUGUAGAU**UCUCACUUGGUAACAGAGCUGGGU**
EIF3H crRNA 1 (E1)	UAAUUUCUACUAAGUGUAGAU**CCAUCAGUGCGAGUAGGAGAAGAU**
EIF3H crRNA 2 (E2)[R]	UAAUUUCUACUAAGUGUAGAU**CAUCGUCUUCGUCAUCAUCUUCUC**
EIF3H crRNA 3 (E3)	UAAUUUCUACUAAGUGUAGAU**CGAGCUGUCAAUGUAGACCAUCUA**
EIF3H crRNA 4 (E4)	UAAUUUCUACUAAGUGUAGAU**UGAUCCAUGGAAGACCUCACAAGG**
EIF3H crRNA 5 (E5)[R]	UAAUUUCUACUAAGUGUAGAU**ACACAGCAGAGUAUUGACAAGUGA**
**RPA primers**	
V. alg F1	CGCATTGTTTGATACACACGCACTGTTTGAGAA
V. alg F2	CTACAACATCGCATTGTTTGATACACACGCA
V. alg F3	CCAAGGCTACAACATCGCATTGTTTGATACA
V. alg F4	CAAAGCCCAAGGCTACAACATCGCATTGTTTG
V. alg F5	ATGTACTACAAAGCCCAAGGCTACAACATCG
V. alg F6	ACACGCACTGTTTGAGAAGTTAACCTCG
V. alg R1	GTGAGTGTACATGTAGTCAACAGAGGATGAA
V. alg R2	GAACGTAATGAGTGAGTGTACATGTAGTCA
V. alg R3	CCAGATGCTGCACATTCAGAACGTAATGAG
V. alg R4	GAACTTATCTGCGCCAGATGCTGCACATTCAGAA
V. alg R5	CAGAACACGAACTTATCTGCGCCAGATGCT
V. alg R6	GTTGGGTGAGTCACATCCCAGAACACG
P. aus F1	CACCGATCCAAGATCAGTCTTCATTGTGAATCTG
P. aus F2	TCAGTCTTCATTGTGAATCTGATTTCAGAGGCG
P. aus F3	CATTGTGAATCTGATTTCAGAGGCGGCTTGC
P. aus F4	GAGGCGGCTTGCTTTTAAAGCTCTCCGTCT
P. aus F5	TTAAAGCTCTCCGTCTCTGTCGTCAAACTT
P. aus F6	AAGCTCTCCGTCTCTGTCGTCAAACTTTTTT
P. aus R1	GGTCATTTGGTTCTTTCCAGTTACCAATCG
P. aus R2	CAACACCAGACCACTGCATTTTAGCTTTAGGTC
P. aus R3	GAGGCGCAACACCAGACCACTGCATTTTAGC
P. aus R4	TTGGCACGAGGCGCAACACCAGACCACTGCATT
P. aus R5	GTATAATGTACAGGGCGGTGTTGGCACGAG
P. aus R6	CATCCACCGCTGAAAGTTGTAATGAATTGG
P. ser F1	CACCGATCCAAGATCAGTCTTCATTGTGAATCTG
P. ser F2	TCAGTCTTCATTGTGAATCTGATTTCAGAGGCG
P. ser F3	CATTGTGAATCTGATTTCAGAGGCGGCTTGC
P. ser F4	GAGGCGGCTTGCTTTTAAAGCTCTCCGTCG
P. ser F5	TTAAAGCTCTCCGTCGCTGTCGTCAAACTA
P. ser F6	AAGCTCTCCGTCGCTGTCGTCAAACTATTTC
P. ser R1	GGTCATTTGGTTCTTTCCAGTTACCAATCG
P. ser R2	CAACACCAGACCACTGCATTTTAGCTTTAGGTC
P. ser R3	GAGGCGCAACACCAGACCACTGCATTTTAGC
P. ser R4	TTGGCACGAGGCGCAACACCAGACCACTGCATT
P. ser R5	ATATAATGTACAGGGCGGTGTTGGCACGAG
P. ser R6	CATCCACCGCTGAAAGTTGTAATGAATTGA
HSP16 F1	GAAGAAATCAAGGTCCAACTTCGTGGACATGAG
HSP16 F2	CAAGGTCCAACTTCGTGGACATGAGCTCAC
HSP16 F3	CAACTTCGTGGACATGAGCTCACGGTCACAG
HSP16 F4	CATGAGCTCACGGTCACAGGAAAACACAGAG
HSP16 F5	ACGGTCACAGGAAAACACAGAGCGGAAGAG
HSP16 R1	CTTTGGACAAGCGTGACTTCACTGAACTGAG
HSP16 R2	GTTAGCAAGCCTTCTTTGGACAAGCGTGACTTC
HSP16 R3	CCACTTCTATTGTTAGCAAGCCTTCTTTGGACA
HSP16 R4	GTCACGTGCCACTTCTATTGTTAGCAAGCCTTC
HSP16 R5	CAGAGCTGGGTCACGTGCCACTTCTATTGTTAG
EIF3H F1	GATGGCAATGTATAAAGAGGCAGAATTTAATGCAG
EIF3H F2	AGAGGCAGAATTTAATGCAGAAAAGCTTGCC
EIF3H F3	AGTGGTCTCAGTTTTGAGGCAATGTTTGAAGAG
EIF3H F4	GTCTCAGTTTTGAGGCAATGTTTGAAGAGATTCC
EIF3H F5	TTGAGGCAATGTTTGAAGAGATTCCACTTGTT
EIF3H R1	AGAAACTCATCTGTTTGGGTATGAGGTGACAAG
EIF3H R2	GAGAGACTGAGAAACTCATCTGTTTGGGTATGAG
EIF3H R3	CTGTGGAGAGACTGAGAAACTCATCTGTTTGG
EIF3H R4	ATAAGACCCTGTGGAGAGACTGAGAAACTCATC
EIF3H R5	TCTAAATAAGACCCTGTGGAGAGACTGAGAAAC

Probe	
FQ Probe	6-FAM (Fluorescein) – TTATT – Iowa Black® FQ
FB Probe	6-FAM (Fluorescein) – TTATT – Biotin
**HiFi cloning of *V. alg tlh* fragment into pOSIP-186**	
pOSIP-KO-tlh-Vec-F	GCAGATAAGTTCGTGTTCTGGGATGTGACTCACCCAACTACTAGTAGCGGCCGC TGCAGG
pOSIP-KO-tlh-Vec-R	GTAAACAATGTGTTAGCAGGGTTGTAGTTCTTCGCCAGCTTGGATCCCCGGGTA CCGAGC
**PCR verification primer for site 1 integration[14]**	
186-O-Primary_P1	CTCATTCGAAACCACCCACCG
186-O-Primary_P2	ACTTAACGGCTGACATGG
186-O-Primary_P3	ACGAGTATCGAGATGGCA
186-O-Primary_P4	GATCATCATGTTTATTGCGTGG
186-Secondary_P1	TCCGGAATGCCTGCATTG
186-Secondary_P2	ACTTAACGGCTGACATGG
186-Secondary_P3	ACGAGTATCGAGATGGCA
186-Secondary_P4	CCCTGGAGCCAAAATATCC
**Colony PCR primers**	
Vtlh-PCR-F	CGAAGAACTACAACCCTGCTAACAC
Vtlh-PCR-R	GAGTCACATCCCAGAACACGAAC
**qPCR primers**	
Vtlh-qPCR-F	CGCATTGTTTGATACACACGCAC
Vtlh-qPCR-R	GAGTCACATCCCAGAACACGAAC

Supplementary Table S2. Summary of the estimated costs per sample using the proposed sensor.

Cost of Consumables (Reagents and Processor Components for 1 reaction)

Component	Cost per Unit	Unit Cost per Sample	Notes
RPA	$4.61 / pellet	$18.44 for 4 pellets	As of 05-19-24; TwistAmp® Basic (96 reactions) for $443
Cas12a	$0.13 / pmol	$3.9 for 30 pmol	As of 05-19-24; NEB EnGen LbaCas12a 2,000 pmol for $263
crRNA	$0.05 / pmol	$2.25 for 45 pmol	As of 11-14-2024; IDT synthesized Cas12a-crRNA, 2 nmol for $96
FB Probe	$0.001 / pmol	$0.03 for 30 pmol	As of 05-20-24; IDT synthesized modified DNA, 250 nmol for $350.86
Buffer	++	++	Negligible
Processor (Acrylic Pieces)	$0.05 / in^2	$0.515 for 10.3 in^2	As of 08-12-24; Acrylic sheet 12" X 12" X 3/32" from McMaster for $7.04
Processor Silicone Pad	$0.05 / cm^3	$0.1 for 2 cm^3	As of 01-31-25; Gallon Ecoflex 00-30 from Smooth-on for $197.91
25mm membrane filter	$1.23 / filter	$1.23 for 1 filter	As of 10-08-24; 25mm 0.8-micron MCE membrane filter, 100/pk for $123.48
Dropper Bottle	$0.61 / dropper	$0.61 for 1 dropper	As of 11-30-24; Nalgene dropper bottle, 2000 ct, from ThermoFisher for $1216.37

Total consumables cost per run	$27.08

Cost of Reusable Incubator (Warmer and Fabrication Materials)

Component	Cost per Unit	Unit Cost per Device	Notes
Rechargeable Hand Warmer	$22.98 / unit	$22.98 for 1 unit	As of 08-21-2024; Unigear Hand Warmer from Amazon for $22.98
Aluminum platform	$0.00005 / mm^2	$0.43 for 9,620 mm^2	As of 07-31-2024; Aluminum Flashing Roll 4in x 10ft 26 Gauge from Amazon for $13.99
Insulating jacket	$0.000007 / mm^2	$0.20 for 28,860 mm^2	As of 07-31-2024; Aluminum Foil Thermal Insulation 24in x 10ft from Amazon for $13.10
Magnets	$0.11 / unit	$0.66 for 6 magnets	As of 11-04-2024; Magnets 4 x 3mm, 80 pcs from Amazon for $8.54
PLA filament	$.015 per gram	$2.48 for 165 grams	As of 11-04-2024; PLA filament, 4kg basic black, from Bambu Labs for $59.96

Total cost for reusable incubator	$26.75

Supplementary Table S3. Metadata for natural seawater samples used in this study. Information includes sample name, date, time, location (description, latitude/longitude, depth), and concurrent environmental conditions. Local weather measurements comprise air temperature, humidity, and water temperature; application (App) measurements comprise temperature, wind, and pressure. Nearest tide data were obtained from buoy measurements.

			Sample Location	Sample Location		Local Weather Measurements			App Measurements			
Sample Name	Date	Time	Description	Latitude, Longitude	Sample Depth	Air Temp	Humidity	Water Temp	Temp	Wind	Pressure	Nearest Tide (Buoy Data)
**A**	19-Jul-25	8:05am	Narragansett Bay, Allen Harbor Marina, North Kingtown, RI	41.62151 N, 71.41185 W	40 cm (center approx)	71.2 F	55%	75.4 F	69 F	4mph N	30.12 inHg	8:22am Low Tide, Quonset Point, RI
**B**	19-Jul-25	4:14pm	Buzzards Bay, Silvershell Beach, Marion, MA	41.69440 N, 70.75666 W	40 cm (center approx)	80.3 F	43%	80.3 F	79 F	11mph SW	30.04 inHg	3:43pm High Tide, Sippican Harbor, MA
**C**	20-Jul-25	7:01am	State Fish Pier, Gloucester, MA	42.61618 N, 70.65105 W	40 cm (center approx)	68.7 F	64%	65.0 F	69 F	10mph S	29.91 inHg	7:39pm High Tide, Gloucester Harbor, MA

Supplementary Table S4. Technology comparison of nucleic acid detection methods for ocean samples.

	Portable CRISPR Ocean Diagnostics (this work)	Standard Fluorescent CRISPR Assay	(RT) qPCR
Implementation	On-site field analysis	Requires sample transport to lab	Requires sample transport to lab
Time to result	< 1 hour	Hours to days*	Hours to days*
Type	Integrated field-deployed devices	Laboratory-based	Laboratory-based
Format	Lyophilized	Fresh/frozen	Fresh/frozen
Sample requirement	Filter-captured samples	Laboratory processing or boiling	Laboratory processing or boiling
Data acquisition	Smartphone analysis of LFA	Plate reader fluorescence output	qPCR amplification plots
Equipment/device requirements	Low-cost 3D printed devices	Fluorescence plate reader	qPCR machine
*Equipment/device cost**	<$30**	>$20,000***	>$40,000****

* Including transportation time.
** For cost break down, please see Supplementary Table 2.
*** Based on Agilent BioTek Synergy HTX Multi-Mode Microplate Reader model S1LF, with a price of $28,357.40.
**** Based on Applied Biosystems QuantStudio 3 Real-Time PCR System, 96-well, with a price of $41,190.00.

References

1 Lesinski, J. M. *et al.* FRETting about CRISPR-Cas Assays: Dual-Channel Reporting Lowers Detection Limits and Times-to-Result. *ACS Sensors* **9**, 3616-3624, doi:10.1021/acssensors.4c00652 (2024).

2 Ramachandran, A. & Santiago, J. G. CRISPR Enzyme Kinetics for Molecular Diagnostics. *Analytical Chemistry* **93**, 7456-7464, doi:10.1021/acs.analchem.1c00525 (2021).

3 Ross, G. M. S., Filippini, D., Nielen, M. W. F. & Salentijn, G. I. J. Unraveling the Hook Effect: A Comprehensive Study of High Antigen Concentration Effects in Sandwich Lateral Flow Immunoassays. *Analytical Chemistry* **92**, 15587-15595, doi:10.1021/acs.analchem.0c03740 (2020).

4 Kaminski, M. M. *et al.* A CRISPR-based assay for the detection of opportunistic infections post-transplantation and for the monitoring of transplant rejection. *Nature Biomedical Engineering* **4**, 601-609, doi:10.1038/s41551-020-0546-5 (2020).

5 Colombo, M. *et al.* Real-time, smartphone-based processing of lateral flow assays for early failure detection and rapid testing workflows. *Sensors & Diagnostics* **2**, 100-110, doi:10.1039/D2SD00197G (2023).

6 Land, K. J., Boeras, D. I., Chen, X.-S., Ramsay, A. R. & Peeling, R. W. REASSURED diagnostics to inform disease control strategies, strengthen health systems and improve patient outcomes. *Nature Microbiology* **4**, 46-54, doi:10.1038/s41564-018-0295-3 (2019).

7 Schang, C. *et al.* A low-cost homogenizing device for in-field and remote DNA and RNA extraction. *HardwareX* **20**, e00592, doi:https://doi.org/10.1016/j.ohx.2024.e00592 (2024).

8 Wu, C., Lillehoj, P. B., Sabet, L., Wang, P. & Ho, C.-M. Ultrasonication on a microfluidic chip to lyse single and multiple Pseudo-nitzschia for marine biotoxin analysis. *Biotechnology Journal* **6**, 150-155, doi:https://doi.org/10.1002/biot.201000224 (2011).

9 Yuan, J., Li, M. & Lin, S. An Improved DNA Extraction Method for Efficient and Quantitative Recovery of Phytoplankton Diversity in Natural Assemblages. *PLOS ONE* **10**, e0133060, doi:10.1371/journal.pone.0133060 (2015).

10 McLachlan, R. H., Dobson, K. L., Schmeltzer, E. R., Vega Thurber, R. & Grottoli, A. G. A review of coral bleaching specimen collection, preservation, and laboratory processing methods. *PeerJ* **9**, e11763, doi:10.7717/peerj.11763 (2021).

11 Holstein, C. A., Griffin, M., Hong, J. & Sampson, P. D. Statistical Method for Determining and Comparing Limits of Detection of Bioassays. *Analytical Chemistry* **87**, 9795-9801, doi:10.1021/acs.analchem.5b02082 (2015).

12 Miller, B. S. *et al.* Sub-picomolar lateral flow antigen detection with two-wavelength imaging of composite nanoparticles. *Biosensors and Bioelectronics* **207**, 114133, doi:https://doi.org/10.1016/j.bios.2022.114133 (2022).

13 Detection Limit Fitting Tool (GitHub, 2022).

14 St-Pierre, F. *et al.* One-Step Cloning and Chromosomal Integration of DNA. *ACS Synthetic Biology* **2**, 537-541, doi:10.1021/sb400021j (2013).

15 Kenkel, C. D. *et al.* Development of Gene Expression Markers of Acute Heat-Light Stress in Reef-Building Corals of the Genus Porites. *PLOS ONE* **6**, e26914, doi:10.1371/journal.pone.0026914 (2011).

16 Kenkel, C. D. *et al.* Diagnostic gene expression biomarkers of coral thermal stress. *Molecular Ecology Resources* **14**, 667-678, doi:https://doi.org/10.1111/1755-0998.12218 (2014).

17 Wong, K. H., & Putnam, H. M. The genome of the mustard hill coral, Porites astreoides. *Gigabyte*, doi:https://doi.org/10.46471/gigabyte.65 (2022).
